# A checklist of the bats of Peninsular Malaysia and progress towards a DNA barcode reference library

**DOI:** 10.1371/journal.pone.0179555

**Published:** 2017-07-25

**Authors:** Voon-Ching Lim, Rosli Ramli, Subha Bhassu, John-James Wilson

**Affiliations:** 1 Institute of Biological Sciences, Faculty of Science, University of Malaya, Kuala Lumpur, Malaysia; 2 International College Beijing, China Agricultural University, Beijing, P. R. China; Brown University, UNITED STATES

## Abstract

Several published checklists of bat species have covered Peninsular Malaysia as part of a broader region and/or in combination with other mammal groups. Other researchers have produced comprehensive checklists for specific localities within the peninsula. To our knowledge, a comprehensive checklist of bats specifically for the entire geopolitical region of Peninsular Malaysia has never been published, yet knowing which species are present in Peninsular Malaysia and their distributions across the region are crucial in developing suitable conservation plans. Our literature search revealed that 110 bat species have been documented in Peninsular Malaysia; 105 species have precise locality records while five species lack recent and/or precise locality records. We retrieved 18 species from records dated before the year 2000 and seven species have only ever been recorded once. Our search of Barcode of Life Datasystems (BOLD) found that 86 (of the 110) species have public records of which 48 species have public DNA barcodes available from bats sampled in Peninsular Malaysia. Based on Neighbour-Joining tree analyses and the allocation of DNA barcodes to Barcode Index Number system (BINs) by BOLD, several DNA barcodes recorded under the same species name are likely to represent distinct taxa. We discuss these cases in detail and highlight the importance of further surveys to determine the occurences and resolve the taxonomy of particular bat species in Peninsular Malaysia, with implications for conservation priorities.

## Introduction

Bats (Order: Chiroptera) are charismatic mammals with ecological importance and comprise about 50% of mammal species in tropical forests and 20% of mammal species worldwide. Davison and Zubaid [1] reported 106 bat species from Peninsular Malaysia but the number is increasing with discoveries of new species. For example, *Kerivoula krauensis* [2] and *Rhinolophus luctoides* [3] were recently recognised on the basis of divergences in mitochondrial DNA sequences and morphology. Francis et al. [4] suggested that the species richness of bats across Southeast Asia may be underestimated by 50%, further intensive surveys may increase the species richness of bats in Peninsular Malaysia specifically [5].

Several published checklists of bat species have covered Peninsular Malaysia as part of a broader region, for example, “Walker’s bats of the world” [6], “Horseshoe bats of the world” [7], and/or in combination with other mammal groups, for example, “A handlist of Malaysian mammals” [8], “The mammals of the Indomalayan region: a systematic review” [9], “Checklist of mammals from Malaysia” [1], and “Red list of mammals for Peninsular Malaysia” [10]. Other researchers have produced comprehensive checklists for particular localities: Krau Wildlife Reserve [11] and Ulu Gombak [5]. To our knowledge, a checklist of bats specifically for the entire geopolitical region of Peninsular Malaysia has never been published. Knowing which species are present in Peninsular Malaysia and their distributions across the region are crucial in developing suitable conservation plans [2, 4].

Bat species are traditionally recognised on the basis of morphological characteristics [12, 13, 14]. However, examination of morphological characters may be of limited service when applied to the identification of sympatric and morphologically similar species [4, 15]. For example, *Hipposideros bicolor* sensu lato is a widespread species complex in Southeast Asia that comprises two species, *H*. *bicolor* and *H*. *atrox*, which are morphologically similar with subtle differences but are acoustically distinct [16, 17]. Molecular techniques such as DNA barcoding could help to resolve problems in species recognition [2] and validate findings from echolocation studies [16, 18]. DNA barcoding can identify individuals to their species by matching a short, standardised DNA sequence, obtained from the unknown individual, to reference sequences from taxonomically verified specimens in the Barcode of Life Datasystems—BOLD [19]. Cryptic species (in the sense of Bickford et al. [20]) are often first detected when their (supposedly conspecific) DNA barcodes fail to match closely and display high divergence with reference sequences on BOLD; demonstrating the potential of DNA barcoding as a species discovery tool [2, 5, 15]. Furthermore, DNA can be extracted from hair, tail membrane and wing punch samples; the collection of which has minimal adverse impacts on live bats [21, 22].

The objectives of this review are (1) to create a checklist of bat species reported from Peninsular Malaysia, and (2) to chart the progress towards a comprehensive DNA barcode reference library for the bat species of this region.

## Materials and methods

### Literature review

A preliminary checklist for Peninsular Malaysia was compiled from published checklists [1, 5, 9, 10, 11, 23]. A search for additional published records of bat species reported from Peninsular Malaysia was conducted through Google Scholar (https://scholar.google.com), Web of Science (https://www.webofknowledge.com), PubMed (http://www.ncbi.nlm.nih.gov/pubmed), Cab Direct (http://www.cabdirect.org) and Biodiversity Heritage Library (http://www.biodiversitylibrary.org) using keywords “Chiroptera”, “bats”, “bat species”, “Peninsular Malaysia”, and “DNA barcoding”. We also requested for data from bat surveys conducted in Peninsular Malaysia directly from government agencies (Department of Wildlife and National Parks and Forest Research Institute Malaysia) and researchers known to be active in this region (Dr. Charles M. Francis and Prof. Dr. Zubaid Akbar Mukhtar Ahmad).

Museum collection numbers of type specimens were obtained from the literature. We used the following abbreviations for museum collections: Natural History Museum, London, UK, (BM(NH)); Centre for Thai National Reference Collections, Bangkok, THAILAND (TNRC); National Museum of Malaysia, Kuala Lumpur, MALAYSIA (MNM); National Museum of Natural History, Washington D.C., USA (USNM); Forschungsinstitut und Natur-Museum Senckenberg, Frankfurt am Main, GERMANY (SMF); Hungarian Natural History Museum, Budapest, HUNGARY (HNHM); National Science Museum, Tokyo, JAPAN (NSMT); Museum National d'Histoire Naturelle, Paris, FRANCE (MNHN), Museum für Naturkunde, Berlin, GERMANY (MNB), National Museum of Natural History Naturalis, Leiden, NETHERLANDS (NMNL), Field Museum of Natural History, Chicago, Illinois, USA (FMNH), and Department of Wildlife and National Parks, MALAYSIA (DWNP). Scientific names were checked against usage in the Mammals of the World list maintained by Dr. Nancy Simmons of the American Museum of Natural History whereas common English (vernacular) names followed the “Field Guide to the Mammals of Southeast Asia” [14]. The current conservation status for each species were obtained from IUCN [24].

### DNA barcoding progress

Based on the checklist obtained as above, we searched the BOLD Taxonomy Browser [19] for the availability of DNA barcodes (the standard COI mtDNA region for animals) on BOLD representing each species. The localities and associated Barcode Index Numbers (BINs) [25] of all public DNA barcodes for the listed species were recorded. A BIN is a molecular operational taxonomic unit with high correspondence to “traditional” species boundaries and also a unique alphanumeric code associated with the DNA barcodes (>500bp) it comprises on BOLD. In several cases detailed below, DNA barcodes are likely to represent certain species based on their placement on taxon identification (taxon ID) trees produced by BOLD v.4 [19] but are not presently recorded as those species (i.e. are unnamed or are recorded under different names). For certain taxa, MEGA 7 [26] was used to construct Neighbour-Joining (NJ) trees of the public DNA barcodes using the Kimura 2-parameter model [27] and bootstrapping with 500 replicates [28].

## Results and discussion

Our literature review produced a checklist of 110 bat species for Peninsular Malaysia. In comparison, Kingston et al. [11] reported 69 species, whereas Davison and Zubaid [1] reported 106 species. Of the 110 bat species in our checklist, 105 species have precise locality records whereas the remaining five lack recent and/or precise locality records for Peninsular Malaysia.Our checklist includes records of bats previously reported under informal names, also known as “dark taxa” [29] (i.e. *Cynopterus* cf. *brachyotis* SUNDA, *C*. cf. *brachyotis* FOREST, *Hipposideros* bicolor131, *H*. bicolor142, *Myotis muricola* “Eastern”). Our review of the available DNA barcodes uncovered ten cases of taxonomic uncertainty (i.e. *Hipposideros larvatus*, *H*. *cervinus*, *H*. *galeritus*, *H*. *armiger*, *Rhinolophus lepidus*, *R*. *stheno*, *Kerivoula hardwickii*, *K*. *minuta*, *Philetor brachypterus* and *Miniopterus medius*), for which we highlight the importance of further research and analyses. The checklist is available on BOLD v.4 [19] in the Checklist section as “A checklist of the bats of Peninsular Malaysia and progress towards a DNA barcode reference library” (CL-PMBAT).

Of the eight families included in the checklist, Vespertilionidae has the highest number of recorded species (n = 44, 36%), followed by Hipposideridae (n = 20, 18%), and Pteropodidae (n = 18, 16%). Nycteridae has the lowest number of recorded species with only one species (0.9%). According to IUCN [24], ten species in our checklist: *Megaerops wetmorei*, *Chaerephon johorensis*, *Coelops robinsoni*, *Hipposideros halophyllus*, *H*. *orbiculus*, *H*. *ridleyi*, *Murina aenea*, *M*. *rozendaali*, *Arielulus societatis* and *Hesperoptenus tomesi* are listed as “Vulnerable”, 15 species are listed as “Near Threatened”, and 71 species are listed as “Least Concern”. Six species: *Hipposideros nequam*, *Rhinolophus convexus*, *Kerivoula krauensis*, *Hesperoptenus doriae*, *Myotis hermani* and *Hypsugo macrotis* are listed as “Data Deficient” whereas eight species have yet to be assessed.

Our search of BOLD [18] revealed that 86 species (78%) have public records on BOLD of which 48 of the 86 (44%) have DNA barcodes from Peninsular Malaysia. This means 62 species (56%) did not have DNA barcodes from Peninsular Malaysia, leaving their taxonomic status and presence in Peninsular Malaysia somewhat unresolved. *Nyctalus noctula* did not have DNA barcodes from any locality in Southeast Asia. Eighty species (73%) of the 86 species with public DNA barcodes were associated with BINs.

**Fig 1:**
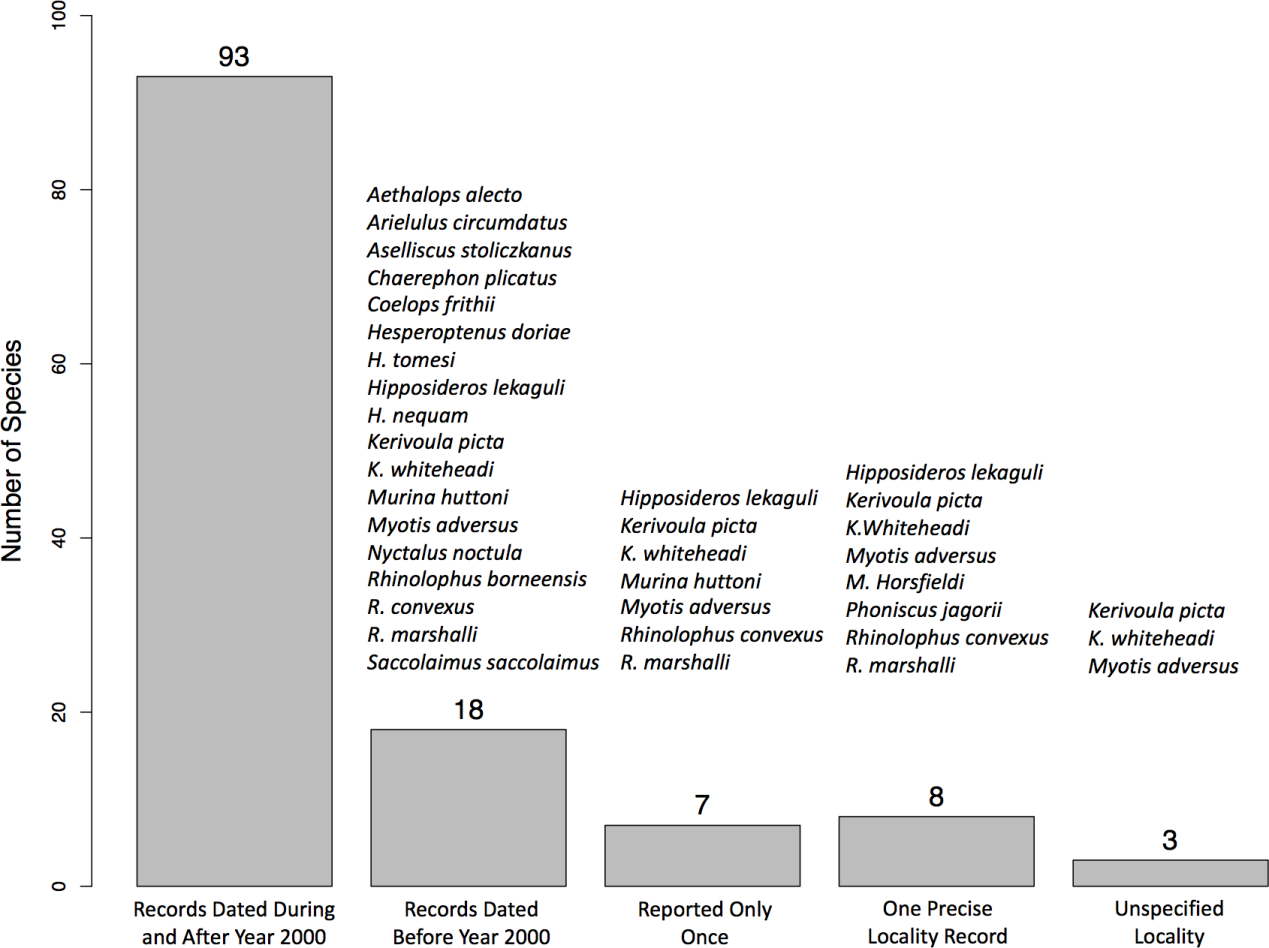
Bat species with recent (dated during or after the year 2000) and old (dated before year 2000) records from Peninsular Malaysia.

We retrieved 18 species (16% of the total species for Peninsular Malaysia) from old records dated before the year 2000 and seven of these species have been recorded in Peninsular Malaysia only once. The lack of recent records for species may be due to sampling biases as some of these species appeared to be restricted to certain localities often with specialised habitat structures [30, 31]. Three species (2.8%) have been reported in Peninsular Malaysia but without any precise localities (Fig 1).

Our literature search revealed that several subspecies have been recently elevated to full species: *Balionycteris seimundi* [32], *Murina peninsularis* [33, 34], *Myotis federatus* [35] and *Rhinolophus morio* [3]. We also discussed the taxonomic status of several other groups (i.e. *Macroglossus sobrinus*, *Hipposideros pomona*, *Rhinolophus lepidus*) based on our NJ analyses of DNA barcodes. We found several cases where specimens recorded under the same species name were assigned to different BINs by BOLD suggesting a need for further examination of these taxa.

The main threat to bats in Peninsular Malaysia is habitat loss, particularly due to expansion of agricultural land and urbanisation [14]. Based on our review, many species appear to be exclusively dependent on certain habitat structures (e.g. *Hipposideros halophyllus* in limestone areas, *Aethalops alecto* in hill and montane forests, and *Pteropus hypomelanus* on islands) and restricted to certain localities (e.g. *Phoniscus jagorii* in Krau Wildlife Reserve, and *Myotis hermani* in Temenggor Forest Reserve). Several species (i.e. *Hipposideros lekaguli*, *H*. *halophyllus*, *H*. *pomona*, *Rhinolophus acuminatus*, *R*. *marshalli*, *R*. *malayanus*) are seemingly restricted to northern Peninsular Malaysia. Currently only two species (i.e. *Pteropus vampyrus* and *P*. *hypomelanus*) are receiving conservation protection from the federal government of Malaysia under the Wildlife Conservation Act 2010. Many species (e.g. *Aselliscus stoliczkanus*, *Chaerephon plicatus*, *R*. *marshalli*, *Kerivoula picta*, *Arielulus circumdatus*) were listed as “Least Concern” by IUCN [24], but the lack of recent records for these species suggests the need for reconsideration of their conservation status in Peninsular Malaysia. Therefore, our literature review highlights (i) the importance of further surveys to determine the presence of particular bat species in Peninsular Malaysia and (ii) areas for further taxonomic work, with implications for the conservation approaches needed for bats in this region.

## Checklist of bat species in Peninsular Malaysia

### Family: Pteropodidae

***Aethalops alecto*** [Thomas, 1923]

*Aethalodes alecto* Thomas, 1923: 251. Indrapura Peak, Sumatra, INDONESIA (Collector unknown; BM(NH) 1923.1.2.1) [36].

*Aethalops alecto* [37].

**Common English name:** Grey Fruit Bat

**Barcode Index Number:** DNA barcodes recorded as *A*. *alecto* are associated with the BIN, BOLD:AAB6984, but there are no DNA barcodes from Peninsular Malaysia.

**Remarks:** Jayaraj et al. [38] commented that “unpublished genetic data suggests the Javan and Borneon forms are distinct”. We could not evaluate the relationship between these two forms and the bats in Peninsular Malaysia due the lack of DNA barcodes from the Peninsular Malaysia and Java.

**IUCN status:** Least Concern

**Recorded at**: **Perak**: Maxwell Hill [23]; **Pahang**: Gunong Benom and Cameron Highlands [23]. *A*. *alecto* is not common and confined to hill and montane forests, normally above 1000 m [14, 23].

***Balionycteris seimundi*** Kloss, 1921

*Balionycteris maculata seimundi* Kloss, 1921: 229. Junction of Tahan and Teku rivers, foot of Gunung Tahan, Pahang, MALAYSIA (E. Seimund, collector; MNM 1/21) [39].

*Balionycteris maculata* [8].

**Common English name:** Spotted-winged Fruit Bat

**Fig 2:**
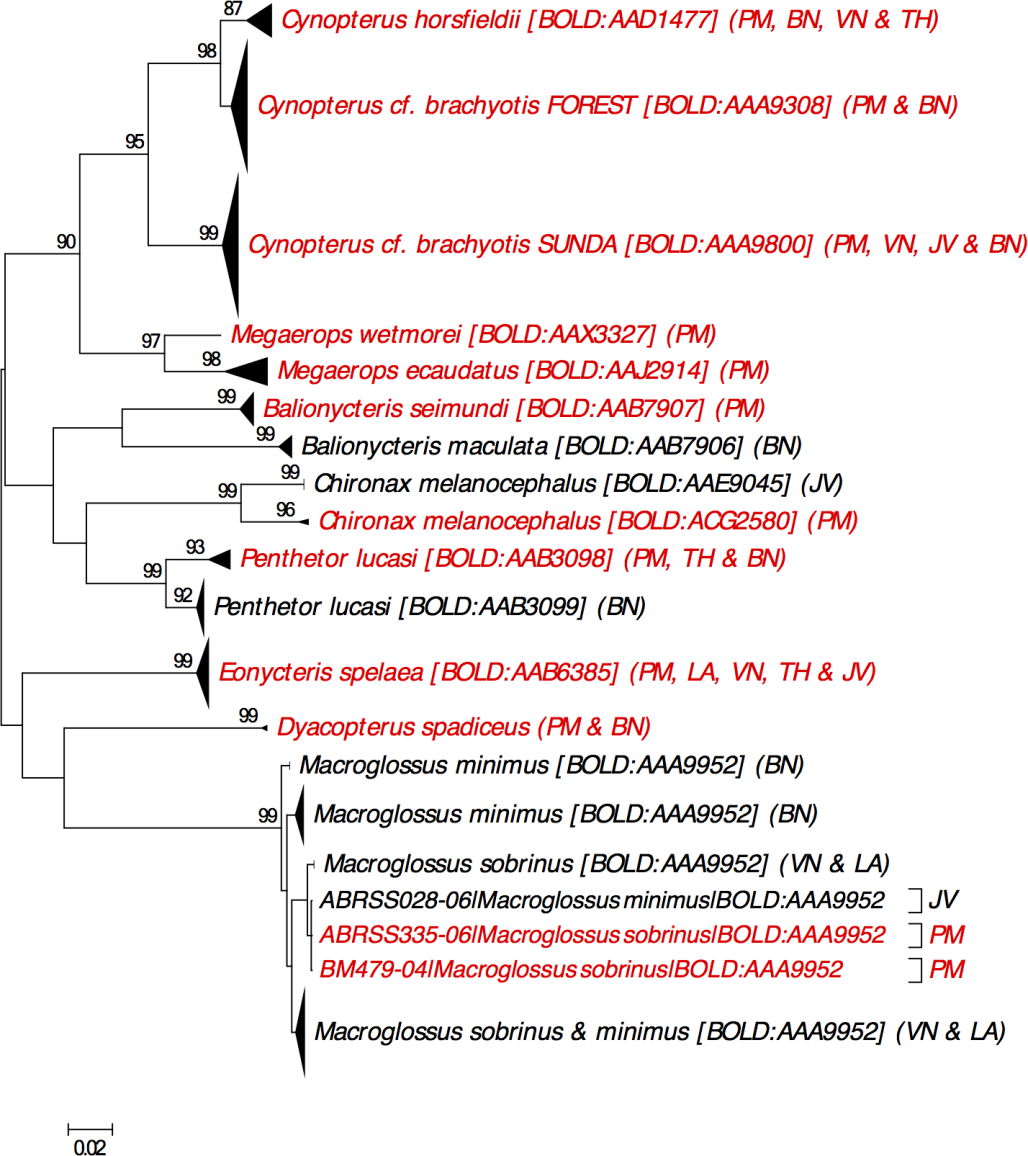
Neighbour-joining tree showing all available DNA barcodes for species in family Pteropodidae reported from Peninsular Malaysia. The percentage of pseudoreplicate trees (≥70%) in which the DNA barcodes clustered together in the bootstrap test (500 pseudoreplicates) are shown above the branches. Abbreviation as follows: PM = Peninsular Malaysia, VN = Vietnam, JV = Java, Indonesia, BN = Borneo (including Sabah, Sarawak, Brunei and Kalimantan), TH = Thailand, LA = Laos.

**Barcode Index Number:** BOLD:AAB7907 (14 DNA barcodes from Peninsular Malaysia; Fig 2).

**Remarks:** Originally described as a subspecies of *B*. *maculata* [9]. Khan et al. [32] reported a high genetic distance (12%) in cytochrome *b* mtDNA between *B*. *maculata* sensu lato populations in Peninsular Malaysia and Borneo and consequently raised *B*. *seimundi* as a distinct species. The same pattern was also observed in COI mtDNA (Fig 2; also see Fig 2 in [4]). Following Khan et al. [32], the name of the taxon in Peninsular Malaysia should be updated to *B*. *seimundi*.

**IUCN status:** Least Concern

**Recorded at:** As *B*. *maculata*: **Pahang**: Gunung Tahan [39], Merapoh [40], Krau Wildlife Reserve [11, 41, 42], Tasik Chini [43], Kuala Atok National Park [44]; **Negeri Sembilan**: Pasoh Forest Reserve [45]; **Perak**: Temengor Forest Reserve [46, 47], Royal Belum State Park [48], Bayor River-Rantau Panjang [49]; **Perlis**: Wang Kelian State Park [50]; **Selangor**: Bukit Lanjan [40], Bangi Forest Reserve [41], Bukit Kutu Wildlife Reserve [51], Ulu Gombak [52–54], Air Hitam Forest Reserve [55], Sungai Dusun Forest Reserve and Lata Bujang Forest Reserve [56]; **Johor:** Endau-Kota Tinggi Forest Reserve [56]; **Kedah**: Ulu Muda Forest Reserve [57].

*B*. *seimundi* tends to roost in small harem groups in sites with bell-shaped cavities and smooth surfaces [11]. Individuals have also been found roosting in crowns of palms, clumps of epiphytic ferns, arboreal ant nests, hollowed arboreal termite nests and hollowed detached large branches [11, 14, 23].

***Chironax melanocephalus*** [Temminck, 1825]

*Pteropus melanocephalus* Temminck, 1825: 190; Gunung Karang, Bantam, west Java, INDONESIA (Collector unknown; Type unknown) [58].

*Chironax melanocephalus* [9].

**Common English name:** Black-capped Fruit Bat

**Barcode Index Number:** BOLD:ACG2580 (1 DNA barcode from Peninsular Malaysia; Fig 2)

**Remarks:** Sing et al. [5] first reported that a DNA barcode collected at Ulu Gombak, shared 95.8% similarity with DNA barcodes of *Chironax melanocephalus* from Java, Indonesia (Fig 2). The DNA barcodes from Java are likely to represent *C*. *melanocephalus* sensu stricto as they were collected from type locality and are assigned to a different BIN (BOLD:AAE9045). Whether several forms of *Chironax* occur in Peninsular Malaysia remains to be determined. Two distinct morphotypes of *C*. *melanocephalus* sensu lato were recently described from Sumatra, Indonesia, neither matching with either of the currently recognised subspecies: *C*. *m*. *melanocephalus* and *C*. *m*. *tumulus* [59]. No DNA barcodes were provided for these specimens but it remains possible that the taxon in Peninsular Malaysia is one of these putative species.

**IUCN status:** Least Concern

**Recorded at: Selangor**: Ulu Gombak [5, 52, 54], Bukit Kutu Wildlife Reserve [51], Sungai Dusun Forest Reserve [56]; **Pahang**: Cameron Highland [23, 60], Krau Wildlife Reserve [11], Fraser Hill Forest Reserve [56]; **Perak**: Royal Belum State Park [48], Bayor River-Rantau Panjang [49]; **Johor:** Endau Kluang Forest Reserve [56]; **Kedah**: Ulu Muda Forest Reserve [57]; **Kelantan**: Air Panas-Gua Musang [61], Gunung Chamah [62].

*C*. *melanocephalus* is common in lowland, hill and montane forests where the species roosts in large colonies in caves and rock shelters but in smaller groups in tree ferns [11, 14, 23].

#### *Cynopterus* cf. *brachyotis* SUNDA

*Pachysoma brachyotis* Müller, 1838: 146. Dewei River, central Kalimantan, INDONESIA (Collector unknown; Type unknown) [63].

*Cynopterus brachyotis* [23].

*Cynopterus* cf. *brachyotis* SUNDA [64]

**Common English name:** Sunda Short-nosed Fruit Bat

**Barcode Index Number:** BOLD:AAA9800 (20 DNA barcodes from Peninsular Malaysia; Fig 2)

**Remarks:** Campbell et al. [64] reported two distinct species under *C*. *brachyotis* sensu lato with a mean divergence of 8.3% in mtDNA (combined control region and cytochrome *b*) between them. The two species are commonly annotated as *C*. cf. *brachyotis* SUNDA and *C*. cf. *brachyotis* FOREST (Fig 2). The SUNDA species is larger than the FOREST species with a longer forearm (>64 mm) and is abundant in highly disturbed habitat (e.g. agricultural and suburban areas) but is absent in mature forests [11, 14, 64, 65].

It is unclear which species represents *C*. *brachyotis* sensu stricto despite the cryptic taxa being widely acknowledged (N Simmons, personal communication). Medway [23] recognised three subspecies of *C*. *brachyotis* in Peninsular Malaysia: (i) *C*. *b*. *brachyotis* found in lowlands and islands in the northern part of Peninsular Malaysia, including Perak, and with a forearm length: 57–68 mm and an ear length: 14.5–18.5 mm; (ii) *C*. *b*. *angulatus* which intergrades with the nominal subspecies at the northern range and has a forearm length: 68–72 mm and an ear length: 18–22 mm; and (iii) *C*. *b*. *altitudinus* foundin the central highlands above 3, 000 ft from Gunung Brinchang, Pahang to Gunung Bunga Buah, Selangor, and with a forearm length: 60–68 mm and an ear length: 18–21 mm. A thorough examination of all relevant types in this genus is required in order to correctly attribute currently existing Linnaean names.

**IUCN status:** As *C*. *brachyotis*: Least Concern

**Recorded at:** These records refer to *C*. *brachyotis* sensu lato, so may represent “SUNDA” or “FOREST”. **Pahang**: Krau Wildlife Reserve [11, 41, 42], Pulau Tioman [23, 64], Merapoh [40], Tasik Chini [43], Kuala Atok National Park [44], Gunung Brinchang [52], Lata Bujang Forest Reserve and Fraser Hill Forest Reserve [56], Cameron Highland [60], Kuala Lipis and Cherating [64]; **Kedah**: Pulau Langkawi [23], Ulu Muda Forest Reserve [57]; **Pulau Pinang**: Pulau Pinang [23]; **Perak**: Pulau Pangkor [23, 64], Temengor Forest Reserve [46, 47], Royal Belum State Park [48, 66], Bayor River-Rantau Panjang and Selama [49], Taping [64]; **Terengganu**: Pulau Redang [23], Pulau Perhentian [64]; **Negeri Sembilan**: Pasoh Forest Reserve [45]; **Kelantan:** Air Panas-Gua Musang [61], Gunung Reng, Gua Musang, and Lojing Highlands [62], Gunung Stong State Park [67]; **Selangor**: Ulu Gombak [5, 40, 52–54], Bukit Kemandul and Bukit Lanjan [40], Bangi Forest Reserve [41], Bukit Kutu Wildlife Reserve [51], Gunung Bunga Buah [52], Air Hitam Forest Reserve [55], Sungai Dusun [64]; **Perlis**: Wang Kelian State Park [50], Perlis State Park and Kangar [64]; **Johor:** Endau-Kluang Forest Reserve and Endau-Kota Tinggi Forest Reserve [56]; **Melaka:** Melaka town [64].

#### *Cynopterus* cf. *brachyotis* FOREST

*Cynopterus brachyotis* FOREST [64].

*Cynopterus* JLE sp. A Francis *et al*. [4] (and as in BOLD).

**Common English name:** Forest Short-nosed Fruit Bat

**Barcode Index Number:** BOLD:AAA9308 (19 DNA barcodes from Peninsular Malaysia; Fig 2)

**IUCN status:** As *C*. *brachyotis*: Least Concern

**Remarks:** The FOREST form is smaller than the SUNDA form with an average forearm length of less than 63 mm and is confined to primary and mature secondary forests [11, 14, 64]. See the remarks on *C*. cf. *brachyotis* SUNDA.

**Recorded at:** Confirmed records of “FOREST” (see above): **Selangor**: Ulu Gombak [5]; **Pahang**: Krau Wildlife Reserve [64]; **Johor**: Endau Rompin [64]; **Perlis**: Perlis State Park and Kuala Perlis [64]; **Kelantan**: Gua Musang [64], **Perak**: Taiping [64], Gunung Stong State Park [67]; **Melaka:** Unspecified [68]; **Terengganu**: Tasik Kenyir and Temenggor Lake [69].

*C*. cf. *brachyotis* FOREST is generally restricted to primary and mature secondary forests [11, 14, 64] and has not been reported from disturbed habitats [65].

***Cynopterus horsfieldii*** Gray, 1843

*Cynopterus horsfieldii* Gray, 1843: 38; Java, INDONESIA (Collector unknown; Type unknown) [70].

**Common English name:** Horsfield’s Fruit Bat

**Barcode Index Number:** BOLD:AAD1477 (3 DNA barcodes from Peninsular Malaysia; Fig 2)

**IUCN status:** Least Concern

**Recorded at: Pahang**: Krau Wildlife Reserve [11, 41], Merapoh [40], Tasik Chini [43], Kuala Atok National Park [44], Cameron Highland, [60, 64], Cherating [64]; **Pulau Pinang**: Pulau Pinang [23], Fraser Hill Forest Reserve [56]; **Selangor**: Bukit Lanjan [40], Bangi Forest Reserve [41], Bukit Kutu Wildlife Reserve [51],Ulu Gombak [52–54]; **Perak**: Bayor River-Rantau Panjang and Selama [49], Taiping [64], Temenggor Lake [69], Temengor Forest Reserve [71]; **Perlis:** Wang Kelian State Park [50], Perlis State Park [64]; **Johor:** Endau Kluang Forest Reserve [56]; **Kedah**: Ulu Muda Forest Reserve [57]; **Kelantan**: Gua Musang [61, 64], Gunung Reng [62], Gunung Stong State Park [67]; **Terengganu**: Tasik Kenyir [69].

*C*. *horsfieldii* has a wide range of habitats (e.g. lowland, hill, and montane forests, mangroves, orchards and plantations) [11] and has been reported roosting gregariously in caves, cavities in limestone caves and rock shelters [11, 14, 23].

***Cynopterus sphinx*** [Vahl, 1797]

*Vespertilio sphinx* Vahl, 1797: 123; Tranquebar, Madras, INDIA (Collector unknown; Type unknown) [72].

*Cynopterus sphinx* [73].

**Common English name:** Greater Short-nosed Fruit Bat

**Barcode Index Number:** DNA barcodes recorded as *C*. *sphinx* are associated with BIN, BOLD:AAA3386, but there are no DNA barcodes from Peninsular Malaysia. Another BIN (BOLD:AAD9139) contains a single DNA barcode of *C*. *sphinx* from India and DNA barcodes recorded as *Pteropus vampyrus*, *P*. *lylei*, and *Rousettus leschenaultii*, which we suspect may represent erroneous records or contamination.

**Remarks:** *C*. *sphinx* resembles *C*. cf. *brachyotis* closely in morphology and both taxa overlap in forearm measurements [14]. However, examination of specimens from Peninsular Malaysia identified as *C*. *sphinx*, *C*. cf. *brachyotis* SUNDA and *C*. cf. *brachyotis* FOREST revealed that *C*. *sphinx* is 8.9% from *C*. cf. *brachyotis* SUNDA and 7.5% divergent from *C*. cf. *brachyotis* FOREST in mtDNA (combined control region and cytochrome *b*) [64]. Our examination of DWNP specimens labelled as *C*. *sphinx* and *C*. cf. *brachyotis* from Peninsular Malayia revealed that the taxa can be differentiatied by a distinctive lower last molar. Specimens labelled as *C*. *sphinx* have lower teeth which are almost uniform in size whereas specimens of *C*. cf. *brachyotis* have non-uniformed lower teeth with extremely small lower last molars [74].

**IUCN status:** Least Concern

**Recorded at: Perak**: Selama [49], Taiping [64]; **Kelantan**: Gunung Reng and Lojing Highlands [62], Gunung Stong State Park [67]; **Perlis**: Wang Kelian State Park [50], Kuala Perlis, Perlis State Park and Kangar [64]; **Pahang**: Cameron Highland [64].

*C*. *sphinx* is commonly found in disturbed habitats and ecotones but not in the forest interior [14, 65].

***Dyacopterus spadiceus*** [Thomas, 1890]

*Cynopterus spadiceus* Thomas, 1890: 235; Baram, Sarawak, MALAYSIA (Charles Hose, collector; BM(NH) 1890.1.28.4) [75].

*Dyacopterus spadiceus* [76].

**Common English name:** Dayak Fruit Bat

**Barcode Index Number:** There are two public DNA barcodes of *D*. *spadiceus* on BOLD, but neither are associated with any BIN due to the short sequence length (<500 bp). One DNA barcode (BM447-04) is from Peninsular Malaysia [4] and our NJ analysis revealed that this DNA barcode exhibited little divergence with the DNA barcode from Kalimantan, Indonesia (BM265-04) (Fig 2).

**IUCN status:** Near Threatened

**Recorded at: Pahang**: Krau Wildlife Reserve [11, 42]; **Perak**: Temengor Forest Reserve [46, 47]; **Selangor**: Bukit Kutu Wildlife Reserve [51], Sungai Dusun Forest Reserve [56]; **Kedah**: Ulu Muda Forest Reserve [57].

*D*. *spadiceus* roosts in tree cavities and ferns and has been recorded in lowland, hill and montane forests, and nearby limetone caves [11, 14. 22].

***Eonycteris spelaea*** [Dobson, 1871]

*Macroglossus spelaeus* Dobson, 1871: 105, 106; Farm Caves, Moulmein, Tenasserim, MYANMAR (Collector unknown; Type unknown) [77].

*Eonycteris spelaea* [78].

**Common English name:** Cave Nectar Bat

**Barcode Index Number:** BOLD:AAB6385 (1 DNA barcode from Peninsular Malaysia; Fig 2)

**IUCN status:** Least Concern

**Recorded at: Pahang**: Krau Wildlife Reserve [11, 41], Pulau Tioman [23, 79], Tasik Chini [43]; **Selangor**: Batu Caves [23], Bukit Kutu Wildlife Reserve [51], Ulu Gombak [52–54]; **Perak**: Temengor Forest Reserve [46, 47], Bayor River-Rantau Panjang and Selama [49]; **Kedah**: Ulu Muda Forest Reserve [57]; **Kelantan:** Air Panas-Gua Musang [61], Gunung Reng, Gua Musang and Lojing Highlands [62], Gunung Stong State Park [67]; **Melaka** [68].

*E*. *spelaea* is a cave dweller and roosts in large colonies with thousands of individuals [11, 14, 23].

***Macroglossus minimus*** [Geoffroy, 1810]

*Pteropus minimus* Geoffroy, 1810: 97; Java, INDONESIA (Leschnault de la Tour, collector; Type unknown) [80].

*Macroglossus minimus* [73].

**Common English name:** Lesser Long-tongued Nectar Bat

**Barcode Index Number:** DNA barcodes recorded as *M*. *minimus* are associated with BIN, BOLD:AAA9952, but there are no DNA barcodes from Peninsular Malaysia.

**IUCN status:** Least Concern

**Remarks:** *M*. *minimus* resembles *M*. *sobrinus* closely in morphology but has a shorter rostrum (26 – 28mm) and muzzle [74], and a deep median groove on the upper lip which is absence in *M*. *sobrinus* [14, 74]. Our examination of DWNP specimens from Peninsular Malaysia labelled as *M*. *minimus* and *M*. *sobrinus* revealed specimens that fit the description of M. Minimus and specimens that fit the description of M. sobrinus supporting the presence of both taxa in Peninsular Malaysia. However, the taxa showed very shallow divergence in COI mtDNA in our NJ analysis with DNA barcodes from both type localities (i.e. Java and Peninsular Malaysia) being grouped together (Fig 2; also see Fig 6 in [4]). It remains unclear whether *M*. *minimus* and *M*. *sobrinus* are actually the same species or whether they represent two taxa that diverged recently. Further analysis of nuclear DNA would be required to determine this and we tentatively retain the taxa as distinct species in our checklist.

**Recorded at: Pahang**: Pulau Tioman [23], Krau Wildlife Reserve [41]; **Selangor**: Kuala Selangor [40], Bangi Forest Reserve [41], Ulu Gombak [52]; **Perak**: Temengor Forest Reserve [47]; Bayor River-Rantau Panjang [49]; **Kelantan**: Air Panas-Gua Musang [61], Gunung Chamah, Gunung Reng, Gua Musang and Lojing Highlands [62], Gunung Stong State Park [67].

*M*. *minimus* has been recorded in mangroves, coastal areas and disturbed areas [14, 23].

***Macroglossus sobrinus*** Andersen, 1911

*Macroglossus minimus sobrinus* Andersen, 1911: 641, 642; Mount Igari, Perak, MALAYSIA (A.L. Butler, presenter; BM(NH) 1898.11.29.1) [81].

*Macroglossus sobrinus* [8].

**Common English name:** Greater Long-tongued Nectar Bat

**Barcode Index Number:** BOLD:AAA9952 (2 DNA barcodes from Peninsular Malaysia; Fig 2).

**IUCN status:** Least Concern

**Remarks:** *Macroglossus sobrinus* was first described as a subspecies of *M*. *minimus* but was later considered as a distinct species [11, 14, 74] (see remarks on *M*. *minimus*).

**Recorded at: Pahang**: Krau Wildlife Reserve [11, 41], Tasik Chini [43], Cameron Highland [60]; **Selangor**: Bangi Forest Reserve [41], Bukit Kutu Wildlife Reserve [51], Ulu Gombak [53, 54]; **Perak**: Bayor River-Rantau Panjang [49]; **Perlis:** Wang Kelian State Park [50]; **Kedah:** Gunung Jerai [50], Ulu Muda Forest Reserve [57]; **Kelantan:** Air Panas-Gua Musang [61], Gunung Chamah, Gunung Reng, Gua Musang and Lojing Highlands [62], Gunung Stong State Park [67].

*M*. *sobrinus* has been recorded in dipterocarp and montane forests, and disturbed areas [14], and has been reported roosting in rolled young banana leaves and pollinating wild banana plants [11].

***Megaerops ecaudatus*** [Temminck, 1837]

*Pachysoma ecaudatum* Temminck, 1837: 94; Padang, West Sumatra, INDONESIA (Collector unknown; Type unknown) [82].

*Megaerops ecaudatus* [82].

**Common English name:** SundaTailless Fruit Bat

**Barcode Index Number:** BOLD:AAJ2914 (7 DNA barcodes from Peninsular Malaysia; Fig 2)

**IUCN status:** Least Concern

**Recorded at: Pahang**: Krau Wildlife Reserve [11, 42], Fraser Hill, Gunung Brinchang, and Cameron Highland [23], Tasik Chini [43]; **Perak**: Temengor Forest Reserve [46, 47], Bidor [76]; **Perlis:** Wang Kelian State Park [50]; **Selangor**: Bukit Kutu Wildlife Reserve [51], Lata Bujang Forest Reserve [56]; **Johor:** Endau-Kota Tinggi Forest Reserve [56]; **Kedah**: Ulu Muda Forest Reserve [57]; **Kelantan:** Gua Musang [62].

*M*. *ecaudatus* predominantly inhabits pristine forest but has been recorded in disturbed forests [11, 14, 23].

***Megaerops wetmorei*** Taylor, 1934

*Megaerops wetmorei* Taylor, 1934: 191; near Tatayan, Cotobato, Mindanao Island, PHILIPPINES (E. H. Taylor, collector; Described based on specimen No. 770 in E.H. Taylor’s collection with unknown current location) [83].

**Common English name:** White-collared Fruit Bat

**Barcode Index Number:** BOLD:AAX3327 (1 DNA barcode from Peninsular Malaysia; Fig 2)

**IUCN status:** Vulnerable

**Remarks:** The species was first recorded in Peninsular Malaysia as a new subspecies, *M*. *w*. *albicollis* in Pasoh Forest Reserve [84] with distinctive white tufts on the shoulders and neck [11, 84]. The type of *M*. *wetmorei* [83] lacked the white neck tufts (which was followed in the description by Corbet and Hill [9]) and has a short tail of 1.5 mm [83]. Specimens of *M*. *w*. *albicollis* from Pasoh Forest Reserve have a short tail of ~4 mm [84] whereas specimens from Krau Wildlife Reserve [11] are tailless. Further analysis, and more DNA barcordes, would be required to determine whether *M*. *w*. *albicollis* deserves to be recognised as a species distinct from *M*. *w*. *wetmorei*.

**Recorded at: Pahang**: Krau Wildlife Reserve [11]; **Negeri Sembilan**: Pasoh Forest Reserve [45, 84]. *M*. *wetmorei* has only been recorded in mature forests [11, 14].

***Penthetor lucasi*** [Dobson, 1880]

*Cynopterus (Ptenochirus) lucasi* Dobson, 1880: 163; Sarawak, MALAYSIA (Frederic A. Lucas, presenter; Described based on a male specimen from collection of Ward’s Museum, Rochester, New York with unknown current location) [85].

*Penthetor lucasi* [76].

**Common English name:** Dusky Fruit Bat

**Barcode Index Number:** BOLD:AAB3098 (1 DNA barcode from Peninsular Malaysia; Fig 2)

**Remarks:** High divergences in cytochrome *b* mtDNA were reported within a population of *P*. *lucasi* in Miri, Sarawak, Borneo (4.9%) and within a population in Kuching, Sarawak (4.7%) [86]. This is congruent with Khan et al. [32] who reported “~5%” divergence in cytochrome *b* mtDNA among specimens from Sarawak. Khan et al. [32] did not include specimens from Peninsular Malaysia whereas Mohd Ridwan and Abdullah [86] included specimens from Kelantan, Peninsular Malaysia. The DNA sequences from Kelantan were clustered with sequences from Kuching, Miri and Sri Aman (Borneo) and demonstrated 3.88% divergence in cytochrome *b* mtDNA from another cluster from Borneo which consists of DNA sequences from Miri and Kuching. DNA barcodes recorded as *P*. *lucasi* are associated with two BINs, BOLD:AAB3098 and BOLD:AAB3099 (Fig 2). Currently no subspecies have been described for *P*. *lucasi* but considering two DNA clusters could occur within a population [86], further analyses including nuclear DNA, morphology and specimens from several localities are required for a taxonomic revision.

**IUCN status:** Least Concern

**Recorded at: Terengganu**: Kenyir Dam [87]; **Pahang**: Gunung Brinchang [11, 23], Cameron Highlands [23], Krau Wildlife Reserve [11, 41], Tasik Chini [43], Fraser Hill Forest Reserve [56], National Park [87]; **Selangor**: Bangi Forest Reserve [41], Bukit Kutu Wildlife Reserve [51], Ulu Gombak [52, 54], Ulu Langat Forest Reserve and Sungai Dusun Game Reserve [88]; **Negeri Sembilan**: Pasoh Forest Reserve [45]; **Perak**: Temengor Forest Reserve [47]; **Kelantan:** Air Panas-Gua Musang [61], Gunung Stong State Park [67]; **Kedah**: Ulu Muda Forest Reserve [57].

*P*. *lucasi* roosts gregariously in caves, rock shelters and rock crevices and occasionally under palm trees in forests [11, 14, 23].

***Pteropus hypomelanus*** Temminck, 1853

*Pteropus hypomelanus* Temminck, 1853: 61; Ternate Island, North Molucca islands, INDONESIA (Collector unknown; Type unknown) [89].

**Common English name:** Island Flying-Fox

**Barcode Index Number:** DNA barcodes recorded as *P*. *hypomelanus* are associated with the BIN, BOLD:AAZ4957, but there are no DNA barcodes from Peninsular Malaysia.

**IUCN status:** Least Concern

**Recorded at: Johor**: Pulau Pemanggil [23]; **Terengganu**: Pulau Redang [23], Pulau Perhentian [23]; **Kedah**: Pulau Paya [23]; **Pahang**: Pulau Tioman [23, 78; 90].

*P*. *hypomelanus* roosts close to shores on islands, under the fronds of coconut palms and branches of trees, and flies to mainland to feed [14, 23].

***Pteropus vampyrus*** [Linnaeus, 1758]

*Vespertilio vampyrus* Linnaeus, 1758: 31; Java, INDONESIA (Collector unknown; Type unknown) [91].

*Pteropus vampyrus* [8].

**Common English name:** Large Flying-Fox

**Barcode Index Number:** A DNA barcode recorded as *P*. *vampyrus* is associated with the controversial BIN, BOLD: AAD9139 (see remarks on *C*. *sphinx*) but there are no DNA barcodes from Peninsular Malaysia.

**IUCN status:** Near Threatened

**Recorded at: Pahang**: Sungai Tembeling [23], Taman Negara [87], Gunung Tahan [92], Tanjung Agas [93]; **Perak**: Temengor Forest Reserve [47], Lenggong,Teluk Memali and Tambun [93]; **Selangor**: Bukit Kutu Wildlife Reserve [51], Ulu Gombak [52]; **Terengganu**: Kenyir Dam [87], Kampung Gong Tengah, Permaisuri and Kampung Kepah [93]; **Johor:** Benut [93].

*P*. *vampyrus* travels a long distance to feed, and often roosts in mangroves, on nipah palms and on open branches of trees [14, 23].

***Rousettus amplexicaudatus*** [Geoffroy, 1810]

*Pteropus amplexicaudatus* Geoffroy, 1810: 96, pl. 4; Timor Island, Lesser Sunda Islands, INDONESIA (Collector unknown; Type unknown) [80].

*Rousettus amplexicaudatus* [8].

**Common English name:** Geoffroy’s Rousette

**Barcode Index Number:** DNA barcodes recorded as *R*. *amplexicaudatus* are associated with the BIN, BOLD:AAC4982, but there are no DNA barcodes from Peninsular Malaysia.

**IUCN status:** Least Concern

**Recorded at: Pahang**: Krau Wildlife Reserve [11], Gunung Brinchang, [23]; **Selangor**: Batu Caves [23], Bukit Kutu Wildlife Reserve [51], Ulu Gombak [53, 54]: **Kedah**: Pulau Langkawi [23], Ulu Muda Forest Reserve [57]; **Perak**: Temengor Forest Reserve [47], Selama [49]; **Melaka**: Unspecified [68].

*R*. *amplexicaudatus* is a cave dweller and sometimes roosts in crevices of large rock boulders in complete darkness [11, 14, 23]

***Rousettus leschenaultii*** [Desmarest, 1820]

*Pteropus leschenaultii* Desmarest, 1820: 110; Pondicherry, INDIA (Collector unknown; Type unknown) [94].

*Rousettus leschenaultii* [8].

**Common English name:** Leschenault’s Rousette

**Barcode Index Number:** DNA barcodes recorded as *R*. *leschenaultii* are associated with the BIN, BOLD:AAB5823, but there are no DNA barcodes from Peninsular Malaysia.

**IUCN status:** Least Concern

**Recorded at**: **Perlis**: Wang Kelian State Park [50]; **Selangor**: Batu Caves based on skeletal remains [95]. *R*. *leschenaultii* roosts primarily in caves and sometimes in wells, mines and cave-like structures [14].

### Family: Emballonuridae

***Emballonura monticola*** Temminck, 1838

*Emballonura monticola* Temminck, 1838: 25, pl. 2; Mountain Munara, Java, INDONESIA (Collector unknown; Type unknown) [82].

**Common English name:** Lesser Sheath-tailed Bat

**Fig 3:**
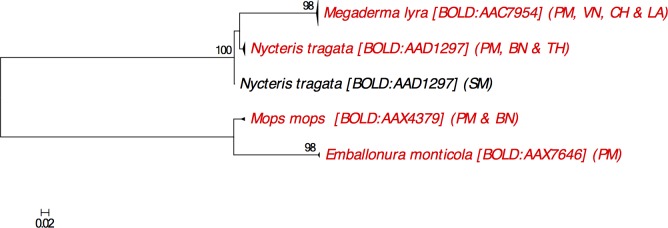
Neighbour-joining tree showing all available DNA barcodes for species in families Emballonuridae, Megadermatidae, Molossidae and Nycteridae reported from Peninsular Malaysia. The percentage of pseudoreplicate trees (≥70%) in which the DNA barcodes clustered together in the bootstrap test (500 pseudoreplicates) are shown above the branches. Abbreviation as follows: PM = Peninsular Malaysia, VN = Vietnam, BN = Borneo (including Sabah & Sarawak of East Malaysia, Brunei and Kalimantan Indonesia), TH = Thailand, LA = Laos, SM = Sumatera Indonesia, CH = China.

**Barcode Index Number:** BOLD:AAX7646 (2 DNA barcodes from Peninsular Malaysia; Fig 3)

**IUCN status:** Least Concern

**Recorded at: Pahang**: Krau Wildlife Reserve [11, 41], Pulau Tioman [23], Tasik Chini [43]; **Terengganu**: Pulau Redang [23]; **Johor**: Pulau Aur [23], Endau-Kota Tinggi Forest Reserve [56]; **Kedah**: Pulau Langkawi [23], Ulu Muda Forest Reserve [57]; **Selangor**: Bukit Lanjan [40], Bukit Kutu Wildlife Reserve [51], Ulu Gombak [52–54]; **Negeri Sembilan**: Pasoh Forest Reserve [45]; **Perak**: Temengor Forest Reserve [46, 47]; **Kelantan:** Gua Musang [62].

*E*. *monticola* is confined to forest and roosts in small groups of two to 20 individuals, normally in shallow caves, rock crevices, hollowed logs, the buttresses of fallen trunks and overhanging earth banks [11, 14, 23].

***Taphozous longimanus*** Hardwicke, 1825

*Taphozous longimanus* Hardwicke, 1825: 525; Calcutta, Bengal, INDIA (Collector unknown; Type unknown) [96].

**Common English name:** Long-winged Tomb Bat

**Barcode Index Number:** DNA barcodes recorded as *T*. *longimanus* are associated with the BIN, BOLD:AAH9837, but there are no DNA barcodes from Peninsular Malaysia.

**IUCN status:** Least Concern

**Recorded at:** Unspecified locations in **Selangor**, **Perak**, and **Pahang** [23]; **Kedah**: Ulu Muda Forest Reserve [57]; **Johor**: Endau-Rompin [23]; **Pahang**: Krau Wildlife Reserve (2 DWNP specimens caught in year 2017).

*T*. *longimanus* roosts in buildings, caves, tree hollows, crowns of palm trees, and among rocks [14, 23]. The latest DWNP specimens were caught in crowns of coconut tree, approximately 3 meters tall (VC Lim, personal observation).

***Taphozous melanopogon*** Temminck, 1841

*Taphozous melanopogon* Temminck, 1841: 287; Bantam, West Java, INDONESIA (Collector unknown; Type unknown) [82].

**Common English name:** Black-bearded Tomb Bat

**Barcode Index Number:** DNA barcodes recorded as *T*. *melanopogon* are associated with the BIN, BOLD:AAD2120, but there are no DNA barcodes from Peninsular Malaysia.

**IUCN status:** Least Concern

**Recorded at: Pahang**: Krau Wildlife Reserve [11, 41]; **Johor**: Pulau Pisang [23]; **Pulau Pinang**: Pulau Pinang [23]; **Selangor**: Pulau Angsa and Batu Caves [23], Bukit Kutu Wildlife Reserve [51], Ulu Gombak [52]; **Kedah**: Pulau Langkawi [23], Ulu Muda Forest Reserve [57]; **Perak**: Bukit Jerneh Cave and Tumang Lembing Cave [30]; **Terengganu**: Bukit Dendong [97].

*T*. *melanopogon* is primarily a cave dweller but has been recorded in lowland and hill forests, plantations and buildings. Individuals have been reported roosting at the entrance of caves, in rock crevices and hollowed dead trees [11, 14, 23].

***Saccolaimus saccolaimus*** [Temminck 1838]

*Taphozous saccolaimus* Temminck, 1838: 14; Java, INDONESIA (Collector unknown; Syntype: BM(NH) 1874.10.26.2) [82].

*Saccolaimus saccolaimus* [98].

**Common English name:** Pouched Tomb Bat

**Barcode Index Number:** The only DNA barcode recorded as *S*. *saccolaimus* is from Vietnam and is not associated with any BIN due to its short sequence length (<500bp).

**IUCN status:** Least Concern

**Recorded at: Pulau Pinang**: Pulau Pinang [23]; **Melaka**: Masjid Tanah [23]; **Selangor**: Ulu Gombak [54].

*S*. *saccolaimus* roosts in the eaves of buildings, hollowed trees and rock crevices [23] with colony size varying from a few to hundreds of individuals [14].

### Family: Megadermatidae

***Megaderma lyra*** Geoffroy, 1810

*Megaderma lyra* Geoffroy, 1810: 190; INDIA (Collector unknown; Type unknown) [99].

**Common English name:** Greater False-Vampire

**Barcode Index Number:** DNA barcodes recorded as *M*. *lyra* are associated with the BIN, BOLD:AAC7954. Two DNA barcodes from Peninsular Malaysia (RONP005-14 and RONP020-14) are not associated with any BIN due to their short sequence length (<500bp) but showed little divergence with other *M*. *lyra* DNA barcodes (Fig 3).

**IUCN status:** Least Concern

**Recorded at: Selangor**: Ulu Gombak [23], Bukit Kutu Wildlife Reserve [51]; **Perak**: Selama [49].

*M*. *lyra* has been reported roosting in shallow caves, buildings and tunnels [14, 23].

***Megaderma spasma*** [Linnaeus, 1758]

*Vespertilio spasma* Linnaeus, 1758: 32; Ternate Island, Moluccas, INDONESIA (Collector unknown; Type unknown) [91].

*Megarderma spasma* [8].

**Common English name:** Lesser False-Vampire

**Barcode Index Number:** DNA barcodes recorded as *M*. *spasma* are associated with the BIN, BOLD:AAC8422, but there are no DNA barcodes from Peninsular Malaysia.

**IUCN status:** Least Concern

**Recorded at: Pulau Pinang**: Unspecified [23]; **Johor**: Pulau Pisang and Pulau Aur [23]; **Kedah**: Pulau Langkawi [23], Ulu Muda Forest Reserve [57]; **Pahang:** Krau Wildlife Reserve [11, 40], Merapoh [40], Tasik Chini [43], Pulau Tioman [79], National Park [87], Kemasul [100]; **Perak**: Bukit Jerneh Cave and Tumang Lembing Cave [30], Temengor Forest Reserve [47]; **Selangor**: Bangi Forest Reserve [41], Bukit Kutu Wildlife Reserve [51], Ulu Gombak [52, 54], Sungai Dusun Game Reserve [88]; **Negeri Sembilan**: Pasoh Forest Reserve [45], Berembun Forest Reserve [101]; **Perlis**: Wang Kelian State Park [50].

*M*. *spasma* has been reported roosting in caves, tunnels, culverts, large tree hollows, rock crevices and abandoned buildings [11, 14, 23]

### Family: Molossidae

***Cheiromeles torquatus*** Horsfield, 1824

*Cheiromeles torquatus* Horsfield, 1824: pt 8; Pulau Pinang, MALAYSIA (John Crawfurd, Esq., collector; Type unknown) [102].

**Common English name:** Naked Bat

**Barcode Index Number:** There are no DNA barcodes recorded under this name on BOLD.

**IUCN status:** Least Concern

**Recorded at: Pulau Pinang**: Unspecified [102]; **Pahang**: Pulau Tioman [9], Krau Wildlife Reserve [11, 41]; **Selangor**: Batu Cave [23], Bukit Kutu Wildlife Reserve [51], Ulu Gombak [52]; **Perak**: Temengor Forest Reserve [47].

*C*. *torquatus* has been reported roosting in caves, tree hollows and abandoned buildings, often with *Mops mops* [11, 14, 23].

***Chaerephon johorensis*** [Dobson, 1873]

*Molossus (Nyctinomus) johorensis* Dobson, 1873: 22; Johor, MALAYSIA (Collector unknown; Type unknown) [103].

*Chaerephon johorensis* [8].

**Common English name:** Johore Wrinkle-lipped Bat

**Barcode Index Number:** There are no DNA barcodes recorded under this name on BOLD.

**IUCN status:** Vulnerable

**Recorded at: Pahang**: Krau Wildlife Reserve [11, 41]; **Johor**: Unspecified [22; 103]; **Selangor**: Unspecified [23]; **Kedah**: Gunung Jerai [104]; **Terengganu:** Belukar Bukit [105].

*C*. *johorensis* has been reported foraging high over the canopy and large rivers in forest [11, 14].

***Chaerephon plicatus*** [Buchannan, 1800]

*Vespertilio plicatus* Buchannan, 1800: 261, pl. 13; Bengal, INDIA (Collector unknown; Type unknown) [106].

*Tadarida plicata* [23].

*Chaerephon plicata* [6].

*Chaerephon plicatus* [98].

**Common English name:** Asian Wrinkle-lipped Bat

**Barcode Index Number:** DNA barcodes recorded as *C*. *plicatus* are associated with the BIN, BOLD:AAK0536, but there are no DNA barcodes from Peninsular Malaysia.

**IUCN status:** Least Concern

**Remarks:** *C*. *plicatus* is considered to be widespread across Peninsular Malaysia [23, 107] despite only a few published locality records [107]. There are no specimens deposited in the DWNP collection. The identity of specimens in the Institute of Medical Research, Malaysia, collection labelled as *C*. *plicatus* could not be confirmed due to the damaged band above head which distinguishes *C*. *plicatus* from *C*. *johorensis*. *C*. *plicatus* closely resembles *Mops mops* but is distinguishable by having five teeth in each upper jaw including extra small anterior upper premolars whereas *M*. *mops* has only four teeth in the upper jaw [14, 23, 74]. Such subtle differences may be difficult to use as identification characteristics in the field for live specimens and consequently may explain the lack of recent records for *C*. *plicatus* in Peninsular Malaysia. There are abundant records for the species in Thailand and Myanmar [107].

**Recorded at: Kedah**: Ulu Muda Forest Reserve [57].

*C*. *plicatus* roosts in large, densely packed colonies and has been reported roosting in buildings [14, 23].

***Mops mops*** [Blainville, 1840]

*Dysopes mops* Blainville, 1840: 101; Sumatra, INDONESIA (Collector unknown; Type unknown) [108].

*Mops mops* [8].

**Common English name:** Sunda Free-tailed Bat

**Barcode Index Number:** BOLD:AAX4379 (1 DNA barcode from Peninsular Malaysia; Fig 3)

**IUCN status:** Near Threatened

**Recorded at: Pahang**: Krau Wildlife Reserve [4, 11, 41]; **Selangor**: Bukit Kutu Wildlife Reserve [51]; **Kedah**: Ulu Muda Forest Reserve [57].

*M*. *mops* is a forest inhabitant and roosts in dead or hollowed trees, often with *Cheiromeles torquatus* [11, 14, 23].

### Family: Nycteridae

***Nycteris tragata*** [Andersen, 1912]

*Petalia tragata* Andersen, 1912: 546; Bidi Caves, Sarawak, Borneo, MALAYSIA (Cecil J. Brooks, Esq., presenter; BM(NH) 1903.3.31.1) [109].

*Nycteris tragata* [8].

**Common English name:** Malayan Slit-faced Bat

**Barcode Index Number:** BOLD:AAD1297 (5 DNA barcodes from Peninsular Malaysia; Fig 3)

**Remarks:** Two names for bats in the genus *Nycteris*, *N*. *javanica* and *N*. *tragata*, have been used in Peninsular Malaysia. All records of *N*. *javanica* in Peninsular Malaysia are from old reports dated before the year 2000 [23, 41, 42, 43, 45, 57]. *N*. *tragata* was considered a synonym of *N*. *javanica* by Medway [23]. The taxa were later considered to be distinct with *N*. *javanica* being confined to Java and some of the surrounding islands [110] whereas *N*. *tragata* occurs in Peninsular Malaysia and Borneo [11, 110]. In this checklist, we treat previous reports of *N*. *javanica* as reports of *N*. *tragata* and recognise only one species, *N*. *tragata*, occurring in Peninsular Malaysia.

**IUCN status:** Near Threatened

**Recorded at: Pahang**: Krau Wildlife Reserve [11], Kuala Atok, National Park [44], Lata Bujang Forest Reserve [56], Jengka [100]; **Perlis**: Wang Kelian State Park [50]; **Johor:** Endau-Kota Tinggi Forest Reserve [56]; **Kelantan**: Air Panas-Gua Musang [61]; **Melaka**: Unspecified [68]; **Negeri Sembilan**: Gunung Angsi Forest Reserve [100, 101]; **Selangor**: Semangkok Forest Reserve [101]; **Perak**: Temengor Forest Reserve [111]; **Kedah**: Bukit Hijau [100].

*N*. *tragata* is confined to mature primary forests and roosts in small groups in hollowed trees, caves, crevices of large boulders and man-made hollows such as culverts [11, 14].

### Family: Hipposideridae

***Aselliscus stoliczkanus*** [Dobson, 1871]

*Asellia stoliczkana* Dobson, 1871: 106; Pulau Pinang, MALAYSIA (Dr. Stoliczka; Type unknown) [77].

*Aselliscus stoliczkanus* [23].

**Common English name:** Trident Roundleaf Bat

**Barcode Index Number:** DNA barcodes recorded as *A*. *stolickanus* are associated with ten BINs, BOLD:AAA6446, BOLD:AAA6447, BOLD:AAA6448, BOLD:AAA6449, BOLD:AAA6450, BOLD:AAA6451, BOLD:ABY9671, BOLD:ABY9672, BOLD:ACF3013, and BOLD:ACF3014. These DNA barcodes are from Vietnam, Laos, China and Myanmar (S1 Fig). Whether any of these DNA barcodes represent the valid *A*. *stolickanus* remains to be determined as none of the DNA barcodes are from bats collected near the type locality (Pulau Pinang, Peninsular Malaysia). There are no DNA barcodes from Peninsular Malaysia.

**IUCN status:** Least Concern

**Recorded at: Pulau Pinang**: Pulau Pinang [77]; **Pahang**: Pulau Tioman [112].

*A*. *stolickanus* roosts in limestone caves and forages in forested and disturbed areas [14, 74]. Both records from Peninsular Malaysia are from islands in northern Peninsular Malaysia. In Thailand, *A*. *stolickanus* is uncommon but widespread [74]. Its rarity in sampling could be due to its ability to detect and avoid mist nets [112].

***Coelops frithii*** Blyth, 1848

*Coelops frithi* Blyth, 1848: 251; Sunderbans, BANGLADESH (R. W. G. Frith, Esq., presenter; Type unknown) [113].

**Common English name:** Asian Tailless Roundleaf Bat

**Barcode Index Number:** DNA barcodes recorded as *C*. *frithii* are associated with two BINs, BOLD:AAF3920 and BOLD:AAF3921 (S2 Fig). One DNA barcode (ABBM313-05) is not associated with any BIN due to its short sequence length (<500bp). There are no DNA barcodes from Peninsular Malaysia.

**IUCN status:** Least Concern

**Recorded at: Selangor**: Ulu Gombak [23], Bukit Kutu Wildlife Reserve [51]; **Kedah**: Ulu Muda Forest Reserve [57].

*C*. *frithii* has been reported foraging in forests and roosting in small groups in caves and hollowed trees [14].

***Coelops robinsoni*** Bonhote, 1908

*Coelops robinsoni* Bonhote, 1908: 4; foot of Mountain Tahan, Pahang, MALAYSIA (Mr Robinson, collector; BM(NH) 1906.10.4.9) [92].

**Common English name:** Malaysian Tailless Roundleaf Bat

**Barcode Index Number:** There are no DNA barcodes recorded under this name on BOLD.

**IUCN status:** Vulnerable

**Recorded at: Pahang**: Krau Wildlife Reserve [11], Gunung Tahan [23, 92]; **Selangor**: Port Swettenham [23].

The type specimen of *C*. *robinsoni* was caught in a young, rolled-up leaf of a wild banana plant [92] and individuals have been reported roosting in hollowed buttresses of large trees and in caves in primary lowland forest [11, 14].

***Hipposideros armiger*** [Hodgson, 1835]

*Rhinolophus armiger* Hodgson, 1835: 699; NEPAL (Collector unknown; Type unknown) [114].

*Hipposideros armiger* [8].

**Common English name:** Greater Roundleaf Bat

**Fig 4:**
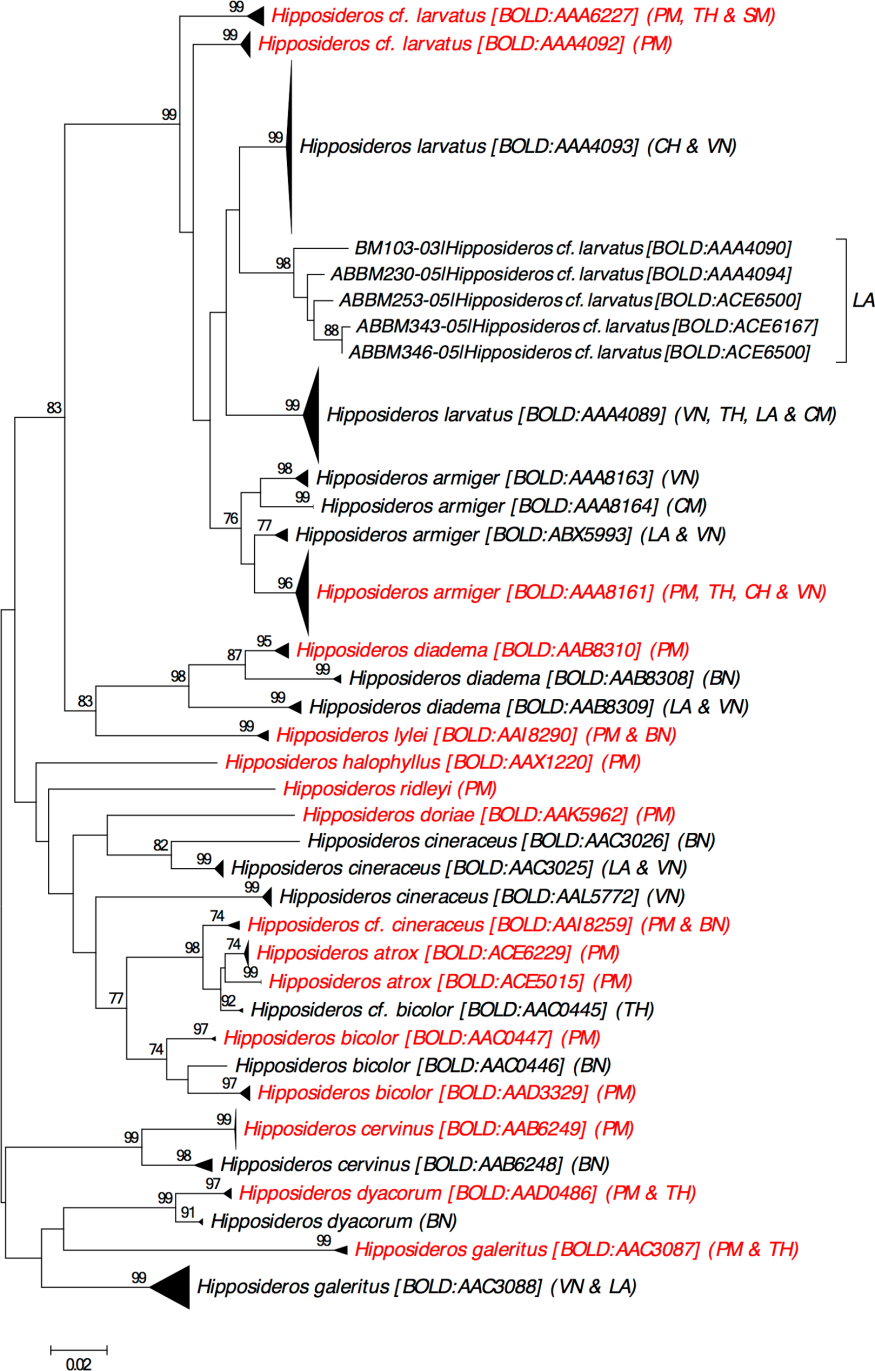
Neighbour-joining tree showing all available DNA barcodes for species in family Hipposideridae reported from Peninsular Malaysia. The percentage of pseudoreplicate trees (≥70%) in which the DNA barcodes clustered together in the bootstrap test (500 pseudoreplicates) are shown above the branches. Abbreviation as follows: PM = Peninsular Malaysia, VN = Vietnam, BN = Borneo (including Sabah & Sarawak of East Malaysia, Brunei and Kalimantan Indonesia), TH = Thailand, LA = Laos, SM = Sumatera Indonesia, CH = China, CM = Cambodia.

**Barcode Index Number:** BOLD:AAA8161 (2 DNA barcodes are from Peninsular Malaysia; Fig 4).

**Remarks:** DNA barcodes recorded as *H*. *armiger* are associated with four BINs, BOLD:ABX5993, BOLD:AAA8161, BOLD:AAA8163, and BOLD:AAA8164. The BIN, BOLD:AAA8161 contains DNA barcodes from across Southeast Asia including Peninsular Malaysia (ABBSI001-04 and ABBSI002-04) whereas the remaining BINs appear to be more geographically restricted (Fig 4). Four subspecies were recognised by Simmons [98]: *H*. *a*. *armiger* (type locality: Nepal), *H*. *a*. *tranninhensis* (type locality: Vietnam), *H*. *a*. *terasensis* (type locality: Taiwan), and *H*. *a*. *fujianensis* (type locality: China). Whether each BIN represents a subspecies or a distinct species and whether BOLD:AAA8161 represents *H*. *armiger* sensu stricto remains to be determined.

**IUCN status:** Least Concern

**Recorded at: Pahang**: Krau Wildlife Reserve [11], Tasik Chini [43], Kenong [100]; **Kedah**: Pulau Langkawi [23], Ulu Muda Forest Reserve [57]; **Perak**: Bukit Jerneh Cave and Tumang Lembing Cave [30], Bayor River-Rantau Panjang [49]; **Negeri Sembilan**: Pasoh Forest Reserve [45]; **Perlis**: Wang Kelian State Park [50]; **Kelantan:** Gunung Reng [62]; **Pulau Pinang**: Bukit Panchor [100].

*H*. *armiger* roosts in large chambers in caves, sometimes in mixed colonies with other species [11, 23]. Individuals have been reported roosting solitarily on *bertam* plants and in crevices of large boulders in forest [11, 14].

***Hipposideros halophyllus*** Hill and Yenbutra, 1984

*Hipposideros halophyllus* Hill and Yenbutra, 1984: 77; Khao Sa Moa Khon (= Khao Sa Moa Khon), Tha Woong (= Ta Woong), Lop Buri, THAILAND (Kitti Thonglongya, collector; TNRC 54–3694) [115].

**Common English name:** Thai Roundleaf Bat

***Barcode Index Number*:** BOLD:AAX1220 (1 DNA barcode from Peninsular Malaysia; Fig 4)

**Remarks:** The BIN also contains a DNA barcode labelled as *H*. *ater* from India which was originally mined from Genbank. The DNA barcode of *“H*. *ater”* is likely to be a case of misidentification as *H*. *halophyllus* and *H*. *ater* are distinct species. *H*. *halophyllus* has a kidney-shaped internarial septum whereas *H*. *ater* has a slightly inflated and triangular internarial septum [30]. It is unlikely that *H*. *ater* occurs in Peninsular Malaysia due to the lack of any records, however, Peninsular Malaysia is included in the distribution range of *H*. *ater* in some literature [1, 9].

**IUCN status:** Vulnerable

**Recorded at: Perak**: Bukit Jerneh Cave and Tumang Lembing Cave [30].

*H*. *halophyllus* has been recorded in and nearby limestone caves in Peninsular Malaysia and Thailand [14, 30]. It is unknown whether *H*. *halophyllus* is strictly confined to limestone areas or this association is an effect of limited sampling but it is likely that the species requires specialised roosting habitat [30].

#### *Hipposideros bicolor* species complex

*Hipposideros bicolor* was first recognised as a cryptic species complex by Kingston et al. [16] who discovered two phonic types under *H*. *bicolor* sensu lato with individuals echolocating at 131 kHz (= *H*. bicolor131) or at 142 kHz (= *H*. bicolor142). The two phonic types are 6.5–6.8% divergent in mtDNA [16] yet morphologically similar and overlap in size [16]. Although the phonic types have been widely recognised as distinct species, some recent reports still use *H*. *bicolor* to represent both phonic types [50, 61, 67, 111] which leads to ambiguity regarding the occurrence of the species. The two phonic types were recently formalised under Latin names: *H*. *bicolor* (= bicolor131) and *H*. *atrox* (= bicolor142) [17]. However, our search of DNA barcodes on BOLD coupled with recent DNA barcoding suggested that the *H*. *bicolor* complex is even more complicated (Fig 4).

***Hipposideros bicolor*** [Temminck, 1834]

*Rhinolophus bicolor* Temminck, 1834: 19. pl. 1; Anjer Coast, Northwestern Java, INDONESIA (Collector unknown; Type unknown) [116].

*Hipposideros bicolor* [8].

*Hipposideros* bicolor131 [16].

**Common English name:** Bicolored Roundleaf Bat

**Barcode Index Number:** BOLD:AAC0447 (2 DNA barcodes from Peninsular Malaysia) and BOLD:AAD3329 (6 DNA barcodes from Peninsular Malaysia). The two BINs showed more than 3% of divergence in COI mtDNA (Fig 4).

**IUCN status:** Least concern

**Recorded at:** The species has been recorded under several names. As *H*. bicolor131: **Pahang**: Krau Wildlife Reserve [11], Bukit Ibam, Kemasul, Jengka, Klau Besar, Kenong and Gunung Aais [100]; **Perak**: Royal Belum State Park [66], Kledang Saiong Forest Reserve [100, 101]; **Melaka:** Unspecified [68]; **Selangor**: Semangkok Forest Reserve [101], Ulu Gombak [5, 101]; **Terengganu**: Gunung Tebu Forest Reserve [101], Pasir Raja, Dungun [15]; **Negeri Sembilan**: Pasoh Forest Reserve (BM454-04 and BM455-04 [4], Berembun Forest Reserve [101], Gunung Angsi Forest Reserve, [100, 101]; **Johor**: Endau Rompin National Park (ABRSS332-06, ABRSS333-06, ABRSS379-06, and BM423-04 [4]), Gunung Panti and Labis Forest Reserve [100]; **Kelantan**: Gunung Stong State Park [100]; **Pulau Pinang**: Bukit Panchor [100]; **Kedah**: Bukit Hijau and Ulu Muda Forest Reserve [100].

As *H*. *bicolor* (could be either *H*. *bicolor* or *H*. *atrox*): **Perak**: Temengor Forest Reserve [111]; **Kelantan**: Air Panas-Gua Musang [61], Lojing Highlands [62]; **Perlis**: Wang Kelian State Park [50]; **Pahang:** Lata Bujang Forest Reserve and Fraser Hill Forest Reserve [56]; **Johor:** Endau-Kota Tinggi Forest Reserve [56].

The *H*. *bicolor* species complex has been recorded in a wide range of habitats (i.e. primary and secondary lowland forests, cultivated areas including rubber plantations, and near limestone areas) [11, 14, 17]. Individuals have been reported roosting in caves, tunnels and rock crevices with other *Hipposideros* species [23, 17].

***Hipposideros atrox*** Andersen, 1918

*Hipposideros gentilis atrox* Andersen, 1918: 381; Semangko Gap, Selangor, MALAYSIA, 2800 ft (A. L. Butler, Esq., presenter; BM(NH) 1901.3.9.4) [117].

*Hipposideros bicolor atrox* [118].

*Hipposideros* bicolor142 [16].

*Hipposideros atrox* [17].

**Common English name:** Lesser Bicoloured Roundleaf Bat

**Barcode Index Number:** BOLD:ACE5015 (2 DNA barcodes from Peninsular Malaysia) and BOLD:ACE6229 (11 DNA barcodes from Peninsular Malaysia). The two BINs showed less than 2% of divergence in COI mtDNA (Fig 4).

**IUCN status:** Not Evaluated but Least Concern as *H*. *bicolor*:

**Recorded at:** As *H*. cf. *bicolor*: **Perlis**: Perlis State Park (ABBSI006-04 and ABBSI007-04 [4]); **Pahang**: Krau Wildlife Reserve (ABBSI011-04, ABBSI015-04 [4]), Kuala Lompat (BM452-04 [4]), Kuala Lipis (ABBSI012-04 [4]), and Bukit Sagu-Kuantan (ABBSI009-04 [4]); **Kelantan**: Dabong (ABBSI010-04 [4]); **Selangor**: Ampang (ABBSI013-04 [4]); **Perak**: Gunung Gajah-Ipoh (ABBSI014-04 [4]); **Negeri Sembilan**: Pasoh Forest Reserve (BM453-04 [4]).

As *H*. bicolor142: **Pahang**: Krau Wildlife Reserve [11], Bukit Ibam, Jengka, Klau Besar, and Kenong [100]; **Selangor**: Semangkok Forest Reserve [101], Ulu Gombak [5, 101]; **Terengganu**: Pasir Raja, Dungun [15], Tasik Kenyir [69], Gunung Tebu Forest Reserve [101]; **Negeri Sembilan**: Berembun Forest Reserve [101], Gunung Angsi Forest Reserve [100, 101]; **Perak**: Temenggor Lake [69], Kledang Saiong Forest Reserve [100]; **Kelantan**: Gunung Stong State Park [100]; **Pulau Pinang**: Bukit Panchor [100]; **Kedah**: Bukit Hijau [100]. Also see records of *H*. *bicolor* sensu lato above.

Roosting colonies of *H*. *atrox* vary from a few to hundreds of individuals [17]. The species has a wide range of habitat: limestone caves, hill and lowland primary forests, secondary forests, and even highly disturbed areas including plantations and human residences [11, 14, 23, 17]. Individuals have been reported roosting with other *Hipposideros* species [17].

***Hipposideros cervinus*** [Gould, 1854]

*Rhinolophus cervinus* Gould, 1854: pl. 34; Cape York and Albany Island, Queensland, AUSTRALIA (Collector unknown, Type unknown) [119].

*Phyllorhina labuanensis* Tomes, 1859: 537; Labuan Island, Borneo, MALAYSIA (Mr. James Motley, collector; BM(NH) 7.1.1.305) [120]

*Hipposideros schneidersi* (misprint = *schneideri*) Thomas, 1904: 722; Upper Langkat, Sumatera, INDONESIA (Collector unknown; BM(NH) 7.1.9.4) [121].

*Hipposideros galeritus schneidersi* [122].

*Hipposideros cervinus labuanensis (schneidersi)* [123].

**Common English name:** Fawn Roundleaf Bat

**Barcode Index Number:** BOLD:AAB6249 (19 DNA barcodes from Peninsular Malaysia; Fig 4)

**Remarks:** Jenkins and Hill [123] described several subspecies under *H*. *cervinus* based on morphometric analyses. They concluded that *H*. *c*. *labuanensis* is the only taxon occuring in Peninsular Malaysia and Borneo and treated *H*. *c*. *schneidersi* as a synonym of *H*. *c*. *labuanensis*. Bates et al. [124] later commented that although both have the typical “*cervinus”* noseleaf and rostrum, *H*. *c*. *schneidersi* and *H*. *c*. *labuanensis* are morphologically different with *H*. *c*. *schneidersi* having a broader zygomatum compared to *H*. *c*. *labuanensis*. This finding was consistent with an earlier taxonomic treatment of *H*. *c*. *labuanensis* and *H*. *c*. *schneidersi* as distinct species [122].

Murray et al. [125] reported that specimens of *H*. *cervinus* sensu lato from Peninsular Malaysia and Sabah (East Malaysia) were 5.5–6.1% divergent in NADH dehydrogenase subunit 2 (ND2) mtDNA. DNA barcodes named as *H*. *cervinus* are associated with two BINs which show a considerably large divergence (Fig 4). Further analyses are required to determine whether specimens from Peninsular Malaysia and Sabah represent the same species (i.e. *H*. *c*. *labuanensis*) or two different species.

**IUCN status:** Least Concern

**Recorded at: Pahang**: Krau Wildlife Reserve [11, 41, 42], Tasik Chini [43], Kuala Atok, National Park [44], Bukit Ibam, Kemasul, Jengka, Klau Besar, Kenong and Gunung Aais [100]; **Terengganu**: Pasir Raja, Dungun [15], Tasik Kenyir [69]; **Negeri Sembilan**: Pasoh Forest Reserve [45], Gunung Angsi Forest Reserve [100, 101], Berembun Forest Reserve [101]; **Perlis:** Wang Kelian State Park [50]; **Selangor**: Ulu Gombak [54, 101], Air Hitam Forest Reserve [55], Semangkok Forest Reserve [101]; **Melaka**: Lata Bujang Forest Reserve [56], Unspecified [68]; **Johor**: Endau- Kluang Forest Reserve and Endau-Kota Tinggi Forest Reserve [56], Gunung Panti and Labis Forest Reserve [100]; **Kedah:** Ulu Muda Forest Reserve [57], Bukit Hijau [100]; **Kelantan**: Air Panas-Gua Musang, [61]; **Perak**: Temenggor Lake [69], Kledang Saiong Forest Reserve, [100]; **Pulau Pinang**: Bukit Panchor [100].

*H*. *cervinus* forages in forest understory and roosts in limestone caves and crevices amongst boulders in very large colonies of up to 100,000 individuals [11, 14].

***Hipposideros cineraceus*** Blyth, 1853

*Hipposideros cineraceus* Blyth, 1853: 410; near Pind Dadan Khan, Salt Range, Punjab, PAKISTAN (W. Theobold, Esq., collector; Type unknown) [126].

**Common English name:** Ashy Roundleaf Bat

**Barcode Index Number:** A DNA barcode (BM460-04) recorded as *H*. cf. *cineraceus* was collected in Pahang, Peninsular Malaysia and associated with the BIN, BOLD:AAI8259 (Fig 4).

**Remarks:** Murray et al. [125] reported two forms of *H*. *cineraceus* sensu lato from Peninsular Malaysia. A specimen from Perak, Peninsular Malaysia had a large forearm (42.9 mm), echolocated at 152 kHz and showed a high divergence (9.2–15.1%) in ND2 mtDNA from other specimens; while a smaller specimen from Pahang, Peninsular Malaysia (forearm = 39.3 mm) echolocated at 144 kHz and showed 10.4–12.2% divergence in ND2 mtDNA. This is congruent with Khan et al. [32] who discovered an average divergence of 8.7% in cytochrome *b* mtDNA among specimens of *H*. *cineraceus* sensu lato from Krau Wildlife Reserve.

Four BINs are associated with DNA barcodes named as *H*. *cineraceus* on BOLD (Fig 4). Our NJ analysis (Fig 4) did not cluster the DNA barcodes from Peninsular Malaysia and Borneo (BOLD:AAI8259) with other DNA barcodes of *H*. *cineraceus* from Vietnam, Laos and Borneo but clustered the barcodes more closely to *H*. *atrox* (BOLD:ACE5015 and BOLD:ACE6229) from Peninsular Malaysia instead. According to Kingston et al. [11], *H*. *cineraceus* resembles *H*. *bicolor/atrox* closely but is distinct with a smaller body size and a slightly raised bump at the internarial septum. In addition, the average echolocation frequency for *H*. *cineraceus* is 144 kHz and for *H*. *atrox* is 142 kHz. Further analyses including more specimens from across the region are required to examine the status of the bats recorded as *H*. *cineraceus* in Peninsular Malaysia. Consequently, we tentatively retained the name in this checklist.

**IUCN status:** Least Concern

**Recorded at: Pahang**: Krau Wildlife Reserve [11], Fraser Hill Forest Reserve [56], Jengka [100]; **Kedah**: Pulau Langkawi [23], Ulu Muda Forest Reserve [57]; **Selangor**: Ampang [23], Bukit Kutu Wildlife Reserve [51], Ulu Gombak [52]; **Johor**: Pulau Pisang [23], Labis Forest Reserve [100]; **Perak**: Temengor Forest Reserve [46–48], Royal Belum State Park [48]; **Kelantan**: Gunung Reng and Gua Musang [62], Gunung Stong State Park [100]; **Melaka:** Unspecified [68]; **Terengganu**: Bukit Dendong [97].

*H*. *cineraceus* roosts in caves or similar structures such as culverts, often with other *Hipposideros* species [11, 14, 23].

***Hipposideros diadema*** [Geoffroy, 1813]

*Rhinolophus diadema* Geoffroy, 1813: 263, pls. 5, 6; Timor Island, INDONESIA (Péron and Lesueur, collector; MNHN 918) [127].

*Hipposideros diadema* [8].

**Common English name:** Diadem Roundleaf Bat

**Barcode Index Number:** BOLD:AAB8310 (7 DNA barcodes from Peninsular Malaysia; Fig 4)

**Remarks:** Murray et al. [125] compared specimens of *H*. *diadema* from Peninsular Malaysia and *H*. *pelingensis* from Kabaena Island, Southeast Sulawesi, and reported that the species have similar body size and were 2.7% divergent in ND2 mtDNA, although they did not observe *H*. *diadema*’s distinctive white spots on *H*. *pelingensis*. In contrast, they reported that specimens of *H*. *diadema* from Peninsular Malaysia and the smaller *H*. *diadema* from Sulawesi are 8.5% divergent in ND2 mtDNA.

DNA barcodes recorded as *H*. *diadema* are associated with three BINs, BOLD:AAB8308, BOLD:AAB8309, and BOLD:AAB8310. Congruent with Murray et al. [125], the three BINs appear to correspond to geographical regions (Fig 4). Four subspecies are recognised under *H*. *diadema* on the basis of morphological characters [128]: *H*. *d*. *diadema* (type locality: Timor Island, Indonesia), *H*. *d*. *nobilis* (type locality: Java, Indonesia), *H*. *d*. *griseus* (type locality: Luzon, Phillippine), *H*. *d*. *masoni* (type locality: Moulmein, Burma = Myanmar). It is likely that the taxon occurring in Peninsular Malaysia represents *H*. *d*. *nobilis* or *H*. *d*. *masoni*. However, further examination of specimens from several localities and examination of the type specimens is required to determine if the taxon in Peninsular Malaysia should be recognised as a distinct species and to assign a valid name. Consequently, we tentatively retained the name *H*. *diadema* in this checklist.

**IUCN status:** Least Concern

**Recorded at: Pahang**: Krau Wildlife Reserve [11, 41, 42], Merapoh [40], Tasik Chini [43], Lata Bujang Forest Reserve and Fraser Hill Forest Reserve [56], Kemasul, Jengka, Kenong and Gunung Aais [100]; **Selangor**: Batu Caves [23], Bangi Forest Reserve [41], Bukit Kutu Wildlife Reserve [51], Ulu Gombak [52–54]; **Pulau Pinang**: Bukit Panchor [23, 100]; **Kedah**: Pulau Langkawi [23], Ulu Muda Forest Reserve [57]; Bukit Hijau [100]; **Negeri Sembilan**: Pasoh Forest Reserve [45]; **Perak**: Temengor Forest Reserve [46–48], Royal Belum State Park [48, 66], Bayor River-Rantau Panjang [49], Temenggor Lake [69]; **Perlis**: Wang Kelian State Park [50]; **Johor**: Endau-Kluang Forest Reserve [56], Gunung Panti and Labis Forest Reserve [100]; **Kelantan**: Air Panas-Gua Musang [61], Gua Musang [62], Gunung Stong State Park [100]; **Melaka**: Unspecified location [68]; **Terengganu**: Tasik Kenyir [69].

*H*. *diadema* has been reported roosting in limestone caves, in crevices of boulders, tree hollows and solitarily under the fronds of palms, in both primary and secondary forests [11, 14, 23].

***Hipposideros doriae*** [Peters, 1871]

*Phyllorhina doriae* Peters, 1871: 326; Sarawak, Borneo, MALAYSIA (Collector unknown; Type unknown) [129].

*Hipposideros sabanus* Thomas, 1898a: 243; Lawas, Northeast Sarawak, Borneo, MALAYSIA (A. H. Everett, collector; Type unknown) [130].

*Hipposideros doriae* [8].

**Common English name:** Least Roundleaf Bat

**Barcode Index Number:** BOLD:AAK5962 (1 DNA barcode from Peninsular Malaysia; Fig 4)

**Remarks:** *H*. *sabanus* is considered a junior synonym of *H*. *doriae* [11, 32, 125].

**IUCN status:** Near Threatened

**Recorded at: Pahang**: Krau Wildlife Reserve, [11, 32], Genung Benom and Tasik Bera [32], Kemasul and Gunung Aais [100]; **Perak**: Maxwell Hill [32], Temenggor Lake [69], Kledang Saiong Forest Reserve [101]; **Selangor**: Ulu Gombak [32], Semangkok Forest Reserve [101]; **Perlis**: Wang Kelian State Park [50]; **Kelantan:** Air Panas-Gua Musang [61]; **Terengganu**: Tasik Kenyir [69]; **Johor**: Gunung Panti and Labis Forest Reserve [100]; **Kedah**: Bukit Hijau [100].

Recorded as *H*. *sabanus* at: **Perak**: Maxwell Hill [23], Temengor Forest Reserve [111]; **Pahang**: Krau Wildlife Reserve [41]; **Negeri Sembilan**: Pasoh Forest Reserve [45]; **Kedah**: Ulu Muda Forest Reserve [57]; **Terengganu:** Bukit Dendong [97].

*H*. *doriae* has been recorded in lowland and submontane forests up to 1500 m [11, 14].

***Hipposideros dyacorum*** [Thomas, 1902]

*Hipposideros dyacorum* Thomas, 1902: 271; Mountain Mulu, Baram, Sarawak, MALAYSIA (Charles Hose, collector; BM(NH) 1894.9.29.10) [131].

**Common English name:** Dayak Roundleaf Bat

**Barcode Index Number:** BOLD:AAD0486 (1 DNA barcode is from Peninsular Malaysia; Fig 4)

**Remarks:** Murray et al. [125] found little divergence in ND2 and RAG1 mtDNA (<1%) between “*dyacorum”* in Peninsular Malaysia and East Malaysia, Borneo. However, our NJ analysis showed >2% divergence between DNA barcodes from Peninsular Malaysia and Sabah, Borneo (Fig 4). We tentatively retained the name *H*. *dyacorum* in our checklist pending further research into these taxa.

**IUCN status:** Least Concern

**Recorded at: Pahang**: Krau Wildlife Reserve [32], Kenong [100]; **Perlis**: Wang Kelian State Park [50], Gua Tekong Siam [132]; **Perak**: Temenggor Lake [69]; **Terengganu**: Tasik Kenyir [69]; **Kelantan**: Gunung Stong State Park [100], Gua Musang (ABBSI020-04).

*H*. *dyacorum* has been reported roosting in caves, under rocks and in tree hollows [14].

***Hipposideros galeritus*** Cantor, 1846

*Hipposideros galeritus* Cantor, 1846: 183; Pulau Pinang, MALAYSIA (Collector unknown; Type unknown) [133].

**Common English name:** Cantor's Roundleaf Bat

**Barcode Index Number:** BOLD:AAC3087 (2 DNA barcodes from Peninsular Malaysia; Fig 4)

**Remarks:** DNA barcodes recorded as *H*. *galeritus* are associated with two BINs, BOLD:AAC3086 and BOLD:AAC3087. The BIN, BOLD:AAC3086 contains DNA barcodes from Peninsular Malaysia and Thailand whereas BOLD:AAC3087 contains DNA barcodes from Vietnam and Laos (Fig 4). DNA barcodes from Peninsular Malaysia and Thailand are likely to represent *H*. *galeritus* sensu stricto as they cover the type locality.

**IUCN status:** Least Concern

**Recorded at: Pulau Pinang:** Unspecified [133]; **Pahang**: Krau Wildlife Reserve [11, 32], Kuala Atok, National Park [44], Cameron Highland [60], Kenong and Gunung Aais [100]; **Selangor**: Batu Caves [23], Ulu Gombak [32, 52, 53], Bukit Kutu Wildlife Reserve [51], Semangkok Forest Reserve [101]; **Negeri Sembilan**: Broga [32], Pasoh Forest Reserve [45], Gunung Angsi Forest Reserve and Berembun Forest Reserve [101]; **Perak**: Maxwell Hill [32], Temengor Forest Reserve [46, 47], Kledang Saiong Forest Reserve [100]; **Perlis**: Wang Kelian State Park [50]; **Johor**: Endau-Kota Tinggi Forest Reserve [56], Gunung Panti and Labis Forest Reserve [100]; **Kedah**: Ulu Muda Forest Reserve [57]; **Kelantan**: Air Panas-Gua Musang [61], Gunung Reng [62].

*H*. *galeritus* has been reported roosting in limestone caves and sighted near large rock boulders in mature lowland forest [11, 14, 23].

***Hipposideros larvatus*** [Horsfield, 1823]

*Rhinolophus larvatus* Horsfield, 1823: 6; Java, INDONESIA (Collector unknown; Type unknown) [102].

*Hipposideros larvatus* [8].

**Common English name:** Intermediate Roundleaf Bat

**Barcode Index Number:** BOLD:AAA4092 (11 DNA barcodes from Peninsular Malaysia) and BOLD:AAA6227 (1 DNA barcode from Peninsular Malaysia; Fig 4)

**Remarks:** Thabah et al. [18] reported that specimens of *H*. *larvatus* sensu lato from the Indo-Malayan region (India, Myanmar, Malaysia, China) have variable echolocation frequencies (~82 kHz to ~100 kHz) and those from Peninsular Malaysia emitted the highest frequency (100–102 kHz). They also reported size variation with female specimens from Peninsular Malaysia having the lightest body mass and shortest forearm. DNA barcodes recorded as *H*. *larvatus* formed five clusters, consistent with geograpahical origin of the sequences (see Fig 5 in [18]). The variations in echolocation, morphology and mtDNA suggest that *H*. *larvatus* is a species complex [18, 32, 125].

DNA barcodes on BOLD recoded as *H*. *larvatus* are associated with eleven BINs. DNA barcodes from Peninsular Malaysia fell into two BINs (Fig 4; see Fig 5 in [18]). One BIN comprises DNA barcodes from Perlis, northern Peninsular Malaysia, and Thailand, while the other contains barcodes from across Peninsular Malaysia. Lim et al. [134] identified the specimens on an island in Peninsular Malaysia (Pulau Tioman) as *H*. *l*. *barbensis* (type locality: Sainte Barbe Island = Pulau Penjantan), however, Thabah et al. [18] stated that *H*. *larvatus* in Malaysia represents *H*. *larvatus* sensu stricto on the basis of their shorter forearms and type locality. Our NJ analysis suggested at least two distinct forms of *H*. *larvatus* are occurring in Peninsular Malaysia (Fig 4) and clustered DNA barcodes of BIN, BOLD:AAA4092 with ABBSI021-04 which shares the same locality with specimens examined by Thabah et al. [18]. We tentatively retained a single name, *H*. *larvatus* for this species complex in this checklist pending further research.

**IUCN status:** Least Concern

**Recorded at: Pahang**: Krau Wildlife Reserve [11, 42], Pulau Tioman [23, 79], Kuala Atok, National Park [44], Fraser Gill Forest Reserve [56], Kemasul, Klau Besar, Kenong and Gunung Aais [100]; **Terengganu**: Pasir Raja, Dunggun [15], Tasik Kenyir [69], Bukit Dendong [97], Gunung Tebu Forest Reserve [101]; **Kedah**: Pulau Langkawi [23], Ulu Muda Forest Reserve [57], Bukit Hijau [100], Gunung Angsi Forest Reserve [100, 101]; **Johor**: Pulau Aur [23], Endau-Kota Tinggi Forest Reserve [56], Gunung Panti and Labis Forest Reserve [100]; **Perak**: Bukit Jerneh Cave and Tumang Lembing Cave [30], Temenggor Lake [69], Kledang Saiong Forest Reserve [100]; **Negeri Sembilan**: Pasoh Forest Reserve [45]; **Perlis**: Wang Kelian State Park [50]; **Selangor**: Bukit Kutu Wildlife Reserve [51], Ulu Gombak [52], Semangkok Forest Reserve,[101]; **Kelantan**: Air Panas-Gua Musang [61], Gunung Reng and Gua Musang [62], Gunung Stong State Park [100]; **Melaka**: Unspecified [68]; **Pulau Pinang**: Bukit Panchor [100].

*H*. *larvatus* has been reported roosting in limestone caves, buildings, old mines rock and crevices in primary and secondary forests [11, 14].

***Hipposideros lekaguli*** Thonglongya and Hill, 1974

*Hipposideros lekaguli* Thonglongya and Hill, 1974: 285; Phu Nam Tok Tap Kwang, Kaeng Khoi, Suraburi, THAILAND, c. 14°34’N, 101°9’E (Dr. Boonsong Lekagul, collector; TNRC 54–2200) [135].

**Common English name:** Boonsoong’s Roundleaf Bat

**Barcode Index Number:** There are no DNA barcodes recorded under this name on BOLD.

**IUCN status:** Near Threatened

**Recorded at: Kedah**: Gunung Keriang and Kodiang [136]. *H*. *lekaguli* roosts in caves and forages in both forested and disturbed areas [14].

***Hipposideros lylei*** Thomas, 1913

***Hipposideros lylei*** Thomas, 1913: 88; Chiendao Cave, 50 miles north of Chiang Mai, THAILAND, 350 meter (Th. H. Lyle, Esq., presenter; BM(NH) 1913.4.18.3) [137].

**Common English name:** Shield-faced Roundleaf Bat

**Barcode Index Number:** BOLD:AAI8290 (1 DNA barcode from Peninsular Malaysia). Another two DNA barcodes from Peninsular Malaysia were not associated with any BINs (Fig 4).

**Remarks:** *H*. *lylei* was once considered to be conspecific with *H*. *pratti* [138]. Although Tate [139] commented that *H*. *pratti* is known from mountainous parts of lower Peninsular Malaysia, we could not find any other records of the species in Peninsular Malaysia. Consequently, we did not include *H*. *pratti* in this checklist.

**IUCN status:** Least Concern

**Recorded at: Perak**: Gunung Tempurung (ABBSI053-04 –ABBSI055-04 [4]); **Pahang**: Krau Wildlife Reserve [11], Bukit Chintamani [23]; **Kedah:** Unspecified caves [23]; **Perlis**: Wang Tangga, Kaki Bukit [140].

*H*. *lylei* roosts primarily in limestone caves and has been recorded in lowland forests [11, 14, 23].

***Hipposideros nequam*** Andersen, 1918 (?)

*Hipposideros nequam* Andersen, 1918: 380, 381; Klang, Selangor, MALAYSIA (W. Davison, collector; BM(NH) 1885.8.1.369) [117].

***Common English name*:** Malay Roundleaf Bat

**Barcode Index Number:** There are no DNA barcodes recorded under this name on BOLD.

**IUCN status:** Data Deficient

**Remarks:** If valid, the species is extremely rare [42, 125] with only two reports from Peninsular Malaysia: Klang (the holotype) and Krau Wildlife Reserve [42]. However, the record from Krau Wildlife Reserve [42] is questionable due to the lack of information regarding the species identification and the absence of any specimens in the DWNP collection. Moreover, Kingston et al. [11] did not report this species in Krau Wildlife Reserve. The fact that the holotype is damaged remains another challenge to resolve the status of *H*. *nequam* [14, 23]. Tate [122] noted that *H*. *nequam* resembles *H*. *bicolor* in forearm length but differs by having greatly reduced anterior lower premolar. Hill [141] also noted the similarities in cranial structure between *H*. *nequam* and *H*. *(bicolor) atrox*. He further commented that *H*. *nequam* has a similar but slightly different cranial structure with “more inflated rostral eminences, shorter, broader premaxillae, blade-like vomer and greatly reduced anterior lower premolar” and larger than *H*. *bicolor* [141]. It is likely that *H*. *nequam* is a synonym of either *H*. *bicolor* or *H*. *atrox* (CM Francis, personal communication) but based on the slight differences between the types of *H*. *nequam* and *H*. *bicolor* as reported by Hill [141] and the locality of the holotype, we tentatively retained the species in our checklist.

**Recorded at: Pahang**: Krau Wildlife Reserve [42](?); **Selangor**: Klang [117].

***Hipposideros orbiculus*** Francis, Kock and Habersetzer, 1999

***Hipposideros orbiculus*** Francis, Kock and Habersetzer, 1999: 259; Abai Siat, southeast Kota Baru, 01°02’ S 101°43’ E, Sumatera Barat, Sumatra, INDONESIA (H. Stephan, collector; SMF 570902) [142].

**Common English name:** Small Disc Roundleaf Bat

**Barcode Index Number:** There are no DNA barcodes recorded under this name on BOLD.

**IUCN status:** Vulnerable

**Remarks:** *H*. *orbiculus* is extremely rare and possibly has a limited distribution with only three known locations: Kota Baru in Sumatra Barat, Rawang-Kuala Selangor and Sungkai Wildlife Forest Reserve in Peninsular Malaysia [125, 142].

**Recorded at: Selangor**: 16+ km from Rawang, on road between Rawang and Kuala Selangor, northwest Kuala Lumpur [142]; **Perak:** recorded at Sungkai Wildlife Reserve in the year 2007 [143].

*H*. *orbiculus* has been reported roosting in drainage pipes and recorded in peat-swamp forest [14].

***Hipposideros pomona*** Andersen, 1918

Hipposideros pomona Andersen, 1918: 380, 381; Haleri, North Coorg, INDIA (A few miles north of Mercara, Coorg District, Karnataka) (G. C. Shortridge; BM(NH) 1918.8.3.4) [117].

*Hipposideros pomona gentilis* [118].

**Common English name:** Large-eared Roundleaf Bat

**Barcode Index Number:** DNA barcodes recorded as *H*. *pomona* are associated with eight BINs, BOLD:AAA4932, BOLD:AAA4933, BOLD: AAA4934, BOLD:AAA4935, BOLD:AAA4936, BOLD:AAA4937, BOLD:AAA4938 and BOLD:AAA4939, but there are no DNA barcodes from Peninsular Malaysia (S3 Fig).

**Remarks:** Andersen [117] first separated *H*. *pomona* and *H*. *gentilis* on the basis of the noseleaf of *H*. *pomona* sensu stricto being broader than the noseleaf of *H*. *gentilis*. Similarly, Corbet and Hill [9] examined ethanol-preserved specimens and commented that *H*. *pomona* sensu stricto lacked of lateral supplementary leaflets. Likewise, Douangboubpha et al. [17] suggested that *H*. *pomona* sensu stricto [9] may represents at least two species: *H*. *pomona* sensu stricto (restricted to Peninsular India) and *H*. *gentilis* (distributed from north-east India into Southeast Asia). DNA sequences of *H*. *pomono* sensu lato from two mitochondrial genes: ND2 and RAG1 fell into two distinct clades in a phylogenetic tree (see Fig 4 in [125]): (i) *H*. *pomona*, *H*. *rotalis* and *H*. *khaokhouayensis* from Laos, and (ii) *H*. *pomona* from Laos, China, Myanmar and Peninsular Malaysia. Specimens of *H*. *pomona* from both groups are morphologically similar [125]. Three subspecies of *H*. *pomona* have been reported from China: *H*. *p*. *sinesis* (Min-Guang coastal region), *H*. *p*. *gentilis* (South Yunnan region) and an undescribed subspecies (Hainan Island), showing 6.0–8.5% divergences in cytochrome *b* mtDNA and 5.2–8.0% divergences in COI mtDNA [144]. Due to the lack of DNA barcodes from Peninsular Malaysia and unresolved taxonomy across the whole Southeast Asia region, we tentatively retained the name *H*. *pomona* in this checklist pending further research.

**IUCN status:** Least Concern

**Recorded at: Perlis**: Bukit Jerneh Cave and Tumang Lembing Cave [30], Bukit Lagi [145]. *H*. *pomona* is a cave dweller and has been recorded from various forest types and disturbed areas [14].

***Hipposideros ridleyi*** Robinson and Kloss, 1911

*Hipposideros ridleyi* Robinson and Kloss, 1911: 241; Botanic Gardens, SINGAPORE (H. N. Ridley, Esq., collector; MNM 2068/11) [146].

**Common English name:** Ridley's Roundleaf Bat

**Barcode Index Number:** Two DNA barcodes recorded as *H*. *ridleyi* (BM470-04 and BM471-04) are not associated with any BIN due to the short sequence length (<500 bp) but are from Peninsular Malaysia (Fig 4).

**IUCN status:** Vulnerable

**Recorded at: Pahang**: Krau Wildlife Reserve [11, 42], Kuala Atok, National Park [44], Tasik Bera Forest Reserve [56], Bukit Ibam, Kemasul and Gunung Aais [100]; **Kedah:** Ulu Muda Forest Reserve [57]; **Johor**: Gunung Panti [100]; **Kelantan**: Gunung Stong State Park [100].

*H*. *ridleyi* has been reported roosting in small groups in fallen tree hollows, culverts, and drainage pipes [11, 14].

### Family: Rhinolophidae

***Rhinolophus acuminatus*** Peters, 1871

Rhinolophus acuminatus Peters, 1871: 308; Gadok, Java, INDONESIA (Collector unknown; MNB 2548/1) [129].

**Common English name:** Acuminate Horseshoe Bat

**Barcode Index Number:** DNA barcodes recorded as *R*. *acuminatus* are associated with two BINs, BOLD:AAB9238 and BOLD:ABY9249. We did not include the only DNA barcode recorded as *R*. *acuminatus* (RONP046-14) from Peninsular Malaysia in our NJ analysis due to its short length (S4 Fig).

**Remarks:** Five subspecies are recognised by Simmons [98]: *R*. *a*. *acuminatus* in Java, *R*. *a*. *sumatranus* in Sumatra and Borneo, *R*. *a*. *circe* in Nias Island, *R*. *a*. *calypso* in Enggano Island, and *R*. *a*. *audax* in Bali and Lombok. Corbet and Hill [9] commented that specimens from the mainland of Southeast Asia (i.e. Thailand, Laos, Cambodia and Peninsular Malaysia) resemble those from Java or Lombok.

**IUCN status:** Least Concern

**Recorded at: Pahang**: Krau Wildlife Reserve [11], Fraser Hill Forest Reserve [56], Gunung Aais [100]; **Kedah**: Ulu Muda Forest Reserve [57]; **Kelantan:** Gunung Reng [62]; **Perak**: Royal Belum State Park [66], Temenggor Lake [69], Temengor Forest Reserve [111]; **Terengganu**: Tasik Kenyir [69].

*R*. *acuminatus* has been reported roosting in caves, tree hollows, and sometimes roosts solitarily or in pairs under palm leaves in mature lowland forests and hills [11, 14].

***Rhinolophus affinis*** Horsfield, 1823

***Rhinolophus affinis*** Horsfield, 1823: 6; Java, INDONESIA (Collector unknown; BM(NH) 79.11.21.70, lectotype) [102].

*Rhinolophus affinis superans* Andersen, 1905: 104; Pahang, MALAYSIA (MNM, presenter; BM(NH) 1900.7.3.2) [147].

**Common English name:** Intermediate Horseshoe Bat

**Fig 5:**
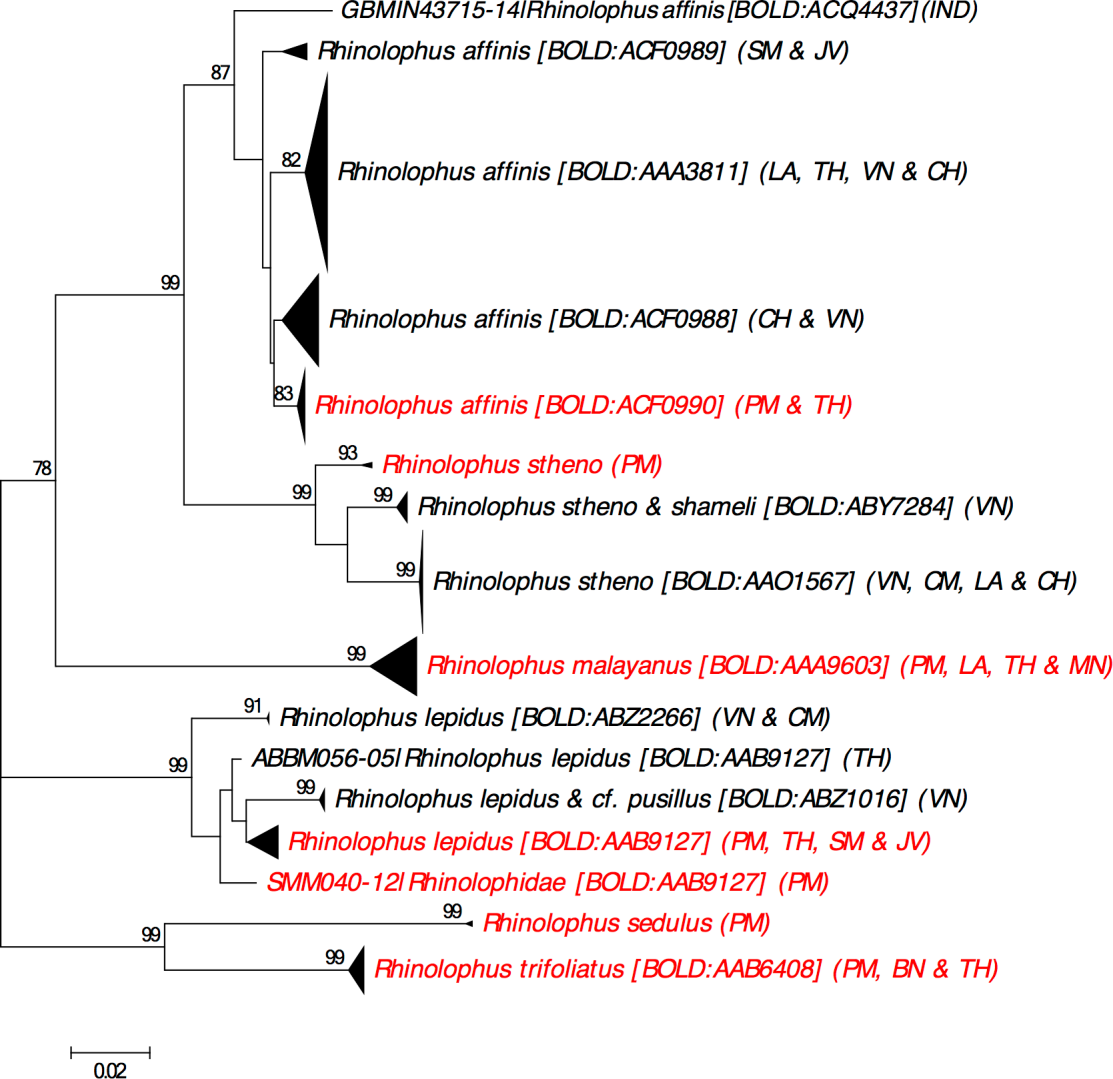
Neighbour-joining tree showing all available DNA barcodes for species in family Rhinolophidae reported from Peninsular Malaysia. The percentage of pseudoreplicate trees (≥70%) in which the DNA barcodes clustered together in the bootstrap test (500 pseudoreplicates) are shown above the branches. Abbreviation as follows: PM = Peninsular Malaysia, VN = Vietnam, BN = Borneo (including Sabah & Sarawak of East Malaysia, Brunei and Kalimantan Indonesia), TH = Thailand, LA = Laos, SM = Sumatera Indonesia, JV = Java Indonesia, IND = India, CH = China, CM = Cambodia, MN = Myanmar.

**Barcode Index Number:** BOLD:ACF0990 (8 DNA barcodes from Peninsular Malaysia; Fig 5).

**Remarks:** DNA barcodes recorded as *R*. *affinis* are associated with five BINs, BOLD:AAA3811, BOLD:ACF0988, BOLD:ACF0989, BOLD:ACF0990, and BOLD:ACQ4437. DNA barcodes from Peninsular Malaysia, Songkhla and Hala Bala (southern Thailand) comprise one BIN, BOLD:ACF0990 (Fig 5).

Nine subspecies are recognised by Simmons [98]: *R*. *a*. *affinis* (type locality: Java), *R*. *a*. *andamanensis* (type locality: South Andaman island), *R*. *a*. *himalayanus* (type locality: Mussoorie, Kumaon Division, northern India), *R*. *a*. *tener* (type locality: Pegu Division = Bago, Myanmar), *R*. *a*. *macrurus* (type locality: Taho, Karennee, Kyah State, Myanmar), *R*. *a*. *nesite* (type locality: Bunguran Island, north Natunas, Indonesia), *R*. *a*. *princeps* (type locality: Lombok, Lesser Sunda Island), *R*. *a*. *hainanus* (type locality: Pouten, Hainan Island), and *R*. *a*. *superans* (type locality: Pahang, Peninsular Malaysia). Morphology (i.e. craniodental and baculum) and molecular (i.e. COI and D-loop regions mtDNA) characteristics provide support that the taxon occurring in Peninsular Malaysia is *R*. *a*. *superans* [148].

**IUCN status:** Least Concern

**Recorded at: Pahang**: Krau Wildlife Reserve [11, 41, 42], Pulau Tioman [23, 79], Merapoh [40], Tasik Chini [43], National Park [44], Tasik Bera Forest Reserve, Fraser Hill Forest Reserve and Lata Bujang Forest Reserve [56], Cameron Highland [60], Kuala Atok, Bukit Ibam, Kemasul, Jengka, Klau Besar, Kenong and Gunung Aais [100]; **Terengganu**: Pasir Raja, Dungun [15], Pulau Redang [23], Tasik Kenyir [69], Bukit Dendong [97], Gunung Tebu Forest Reserve [101]; **Perak**: Lenggong [23], Bukit Jerneh Cave and Tumang Lembing Cave [30], Temengor Forest Reserve [46–48], Royal Belum State Park [66], Temenggor Lake [69], Kledang Saiong Forest Reserve [100, 101]; **Selangor**: Batu Caves [23], Bukit Kutu Wildlife Reserve [51], Ulu Gombak [120]; **Negeri Sembilan**: Pasoh Forest Reserve [45], Gunung Angsi Forest Reserve [100, 101], Berembun Forest Reserve [101]; **Perlis**: Wang Kelian State Park, [50]; **Johor**: Endau-Kota Tinggi Forest Reserve [56], Gunung Panti [100]; **Kedah**: Ulu Muda Forest Reserve [57, 100], Bukit Hijau [100]; **Kelantan**: Air Panas-Gua Musang [61], Gunung Reng and Gua Musang [62], Gunung Stong State Park [67], Gunung Stong State Park [100]; **Melaka**: Unspecified [68]; **Pulau Pinang**: Bukit Panchor [100].

*R*. *affinis* inhabits both primary and secondary forests, and roosts in limestone caves [11, 14, 23].

***Rhinolophus borneensis*** Peters, 1861

*Rhinolophus borneensis* Peters, 1861: 709; Labuan island, north Borneo, MALAYSIA (Collector unknown; Type unknown) [149].

*Rhinolophus chaseni* Sanborn, 1939: 38; Pulo Condore = Con Son Island, south VIETNAM (C. B. Kloss, collector; BM(NH) 21.10.8.3) [150].

*Rhinolophus borneensis chaseni* [9].

**Common English name:** Bornean Horseshoe Bat

**Barcode Index Number:** DNA barcodes of *R*. *borneensis* are associated with a BIN, BOLD:AAC3741, but there are no barcodes from Peninsular Malaysia. DNA barcodes recorded as *R*. *chaseni* are not from Peninsular Malaysia and are associated with a BIN, BOLD:AAB4878, which also contains a single DNA barcode of *R*. *shameli* (ABRVN329-06).

**Remarks:** *R*. *chaseni* was previously recognised as a subspecies of *R*. *borneensis (R*. *b*. *chaseni)* [9] occurring in Peninsular Malaysia while the the nominal subspecies *R*. *b*. *borneensis* occurred in Borneo [151]. However, Francis et al. [4] reported that DNA barcodes (COI mtDNA) of *R*. *borneensis* and *R*. *chaseni* did not cluster together as conspecific (see Fig 3 in [4]). Likewise, Kruskop [152] reported “about” 13% divergence in COI mtDNA between *R*. *chaseni* from Vietnam and *R*. *borneensis* from Borneo. However, due to the lack of any DNA barcodes from Peninsular Malaysia we could not further clarify the status of “*R*. *borneensis”* in Peninsular Malaysia. Consequently, we tentatively retained the name *R*. *borneensis* in this checklist pending further research.

**IUCN status:** Least Concern

**Recorded at: Pahang**: Pulau Tioman [79]; **Perlis**: Wang Pinang [153]. According to Khan et al. [32], *R*. *borneensis* is likely to be very rare in Peninsular Malaysia.

***Rhinolophus chiewkweeae*** Yoshiyuki and Lim, 2005

*Rhinolophus chiewkweeae* Yoshiyuki and Lim, 2005: 29; Gunung Ledang, Tangkak, Muar, Johor, MALAYSIA, 1276 m (Boo-Liat Lim, collector; NSMT-M 33472) [13].

**Common English name:** Chiewkwee's Horseshoe Bat

**Barcode Index Number:** There are no DNA barcodes recorded under this name on BOLD.

**Remarks:** *R*. *pearsonii* is reported to occur in Peninsular Malaysia [9] although we could not find any precise locality reports. It is likely that the records of *R*. *pearsonii* from Peninsular Malaysia, if valid, may actually represent *R*. *chiewkweeae* [154]. However, our NJ analysis revealed that the DNA barcodes under these names (but not including Peninsular Malaysia specimens) were 12% divergent in COI mtDNA (see Fig 3 in [154]).

**IUCN status:** Not Evaluated

**Recorded at: Melaka**: Asahan Forest Reserve [13]; **Johor**: Gunung Ledang and Labis Forest Reserve [13]; **Kedah**: Lubok Semilan, Ulu Melaka in Pulau Langkawi and Weng Subcatchment Area in Ulu Muda Forest Reserve Forest Reserve [13]; **Perlis**: Wang Kelian State Park [50]; **Perak**: Temenggor Lake [69]; **Terengganu**: Tasik Kenyir [69], Sungai Buweh [154].

*R*. *chiewkweeae* has been reported from lowland, hill and submontane dipterocarp forests, and an island [13, 154]. In Peninsular Malaysia, all reported individuals were caught in mature and secondary dipterocarp forests [154]. The low capture rate of *R*. *chiewkweeae* suggested that the population density of the species in Peninsular Malaysia is likely to be very low [50, 154]

***Rhinolophus coelophyllus*** Peters, 1867

Rhinolophus coelophyllus Peters, 1867: 426, pl. 35; Salween River = Thanlwin River, Burma = MYANMAR (Collector unknown; MNB 3143) [155].

**Common English name:** Croslet Horseshoe bat

**Barcode Index Number:** DNA barcodes recorded as *R*. *coelophyllus* are associated with the BIN, BOLD:ACE9393, but there are no DNA barcodes from Peninsular Malaysia.

**Remarks:** *R*. *shameli* was previously considered a subspecies of *R*. *coelophyllus* [156] but the examination of specimens from Thailand and Cambodia suggested that they are distinct species on the basis of the smaller size of *R*. *coelophyllus* and a differently shaped rostral part of the skull [157]. Our search of BOLD found that the two names are associated with different BINs. DNA barcodes recorded as *R*. *shameli* are associated with three BINs, BOLD:AAB4877, BOLD:AAB4878 and BOLD:ABY7284 (The BIN, BOLD:ABY7284 also contained DNA barcodes of *R*. *stheno* and therefore, may be erroneous) whereas DNA barcodes recorded as *R*. *coelophyllus* are associated with one BIN, BOLD:ACE9393. Specimens labelled as *R*. *shameli* from Kedah (BM(NH) 1898.10.1.1) and Pulau Langkawi (BM(NH) 1968.821 and BM(NH) 1968.822) are smaller and represent *R*. *coelophyllus* [157].

**IUCN status:** Least Concern

**Recorded at: Kedah**: Pulau Langkawi and mainland Kedah [23]; **Perlis**: mainland Perlis [23], Wang Kelian State Park, [50]; **Selangor**: Bukit Kutu Wildlife Reserve [51].

*R*. *coelophyllus* has been recorded in forests near limestone hills and once in a house, and roosts in limestone caves in large colonies with hundreds of individuals [14, 23].

***Rhinolophus convexus*** Csorba, 1997

Rhinolophus convexus Csorba, 1997: 343; Gunung Jasar, Tanah Rata, Cameron Highlands, Pahang State, MALAYSIA, 4°28’ N, 101° 22’ E, 1600m (G. Csorba and F. Zilahy, collector; HNHM 95.55.14) [158].

**Common English name:** Convex Horseshoe Bat

**Barcode Index Number:** There are no DNA barcodes recorded under this name on BOLD.

**IUCN status:** Data Deficient; the uncertain status could be due to the rarity of this species with only three or four known specimens [159].

**Recorded at: Pahang**: Gunung Jasar at Tanah Rata in Cameron Highlands [158]. *R*. *convexus* is known only from upper montane rainforest with elevations of 1600 m and above in Peninsular Malaysia [14] and is possibly endemic to Malaysia [159].

***Rhinolophus lepidus*** Blyth, 1844

Rhinolophus lepidus Blyth, 1844: 486; Calcutta, INDIA (Collector unknown; Type unknown) [160].

*Rhinolophus refulgens* Andersen, 1905: 124, pl. 4; Gunung Igari, Perak, MALAYSIA, 2000 ft. (A. L. Butlerm Esq., presenter; BM(NH) 1898.11.29.2) [161].

*Rhinolophus lepidus refulgens* [9].

**Common English name:** Blyth’s Horseshoe Bat

**Barcode Index Number:** BOLD:AAB9127 (5 DNA barcodes from Peninsular Malaysia; Fig 5)

**Remarks:** DNA barcodes of *R*. *lepidus* are associated with three BINs (BOLD:AAB9127, BOLD:ABZ1016 and BOLD:ABZ2266; Fig 5). The BIN, BOLD:ABZ1016 contains DNA barcodes recorded as *R*. *lepidus* and *R*. *pusillus*.

Some authors considered *R*. *refulgens* as a subspecies of *R*. *lepidus* [9, 11, 12] while some considered them to be distinct [50, 67]. Our NJ analysis (Fig 5) suggested that the DNA barcodes recorded as *R*. *lepidus* from Peninsular Malaysia may be distinct from DNA barcodes recorded as *R*. *lepidus* from Indochina. Similarly, Bumrungsri et al. [162] commented that *R*. *lepidus* from Peninsular Malaysia may represent a distinct taxon and the appropriate name would be *R*. *refulgens* based on the type locality. Due to the lack of DNA barcodes from the type locality of *R*. *lepidus*, India for comparison, we could not determine if DNA barcodes from Peninsular Malaysia represent the nominate *R*. *lepidus* or *R*. *refulgens*. Consequently, we tentatively retained only the name *R*. *lepidus* in this checklist (Fig 5).

**IUCN status:** Least Concern

**Recorded at: Pahang**: Krau Wildlife Reserve [11, 42], Kuala Atok, National Park [44], Lata Bujang Forest Reserve [56], Cameron Highland [60], Pulau Tioman [79], Bukit Ibam, Kemasul, Jengka, Klau Besar, Kenong and Gunung Aais [100]; **Terengganu**: Pasir Raja, Dungun [15], Tasik Kenyir [69], Gunung Tebu Forest Reserve [101]; **Perak**: Temengor Forest Reserve [46, 47, 111], Temenggor Lake [69]; **Perlis**: Wang Kelian State Park, [50], Gunung Stong State Park [100]; **Selangor**: Bukit Kutu Wildlife Reserve [51], Semangkok Forest Reserve [101]; **Johor**: Endau-Kota Tinggi Forest Reserve [56], Labis Forest Reserve [100]; **Kedah**: Ulu Muda Forest Reserve [57, 100], Bukit Hijau [100]; **Kelantan**: Air Panas-Gua Musang [61], Gunung Reng and Gua Musang [62]; **Melaka**: Unspecified [68]; **Pulau Pinang**: Bukit Panchor [100]; **Negeri Sembilan**: Gunung Angsi Forest Reserve [100, 101], Berembun Forest Reserve [101].

As *R*. *refulgens*: **Perak**: Maxwell Hill [23]; **Pahang**: Pulau Tioman [23], Krau Wildlife Reserve [41]; **Johor**: Pulau Pemanggil and Pulau Aur [23]; **Negeri Sembilan**: Pasoh Forest Reserve [45]; **Kelantan**: Gunung Stong State Park [67].

*R*. *lepidus* inhabits mature lowland and hill forests and has been reported roosting in caves and rock crevices, often with *R*. *stheno* [11, 14]

***Rhinolophus morio*** Gray, 1842

*Rhinolophus morio* Gray, 1842: 257; SINGAPORE (Collector unknown; BM(NH) 1840.5.14.36) [163].

*Rhinolophus luctus morio* [134].

**Barcode Index Number:** There are no DNA barcodes recorded under this name on BOLD. However, DNA barcodes recorded as *R*. *luctus* are associated with the BIN, BOLD: AAD0380. There are no DNA barcodes from Peninsular Malaysia.

**Remarks:** *R*. *morio* was recently recognised as distinct from *R*. *luctus* based on the ratio of zygomatic width to mandible length in cranial measurements and the unique Y-autosomal translocation in karyotype [3].

**IUCN status:** Not Evaluated but Least Concern as *R*. *luctus*

**Recorded at: Kuala Lumpur**: Gombak Setia [3]; **Selangor**: Templer Park-Rawang, [3]; **Pahang**: Pulau Tioman [134]; **Melaka**: Unspecified [163]. Specimens of *R*. *morio* were collected in lowland dipterocarp forest [3].

Recorded as *R*. *luctus* at: **Pahang**: Krau Wildlife Reserve [11, 41, 42], Bukit Renggit [40], Tasik Chini [43], Cameron Highland [60], Gunung Aais [100]; **Selangor**: Bangi Forest Reserve [41], Bukit Kutu Wildlife Reserve [51], Ulu Gombak [52, 54, 101], Sungai Dusun Forest Reserve [56]; **Kedah**: Ulu Muda Forest Reserve [57]; **Kelantan**: Air Panas-Gua Musang [61]; **Perak**: Royal Belum State Park [66], Temenggor Lake [69], Temengor Forest Reserve [111]; **Melaka**: Unspecified [68]; **Terengganu**: Tasik Kenyir [69]; **Negeri Sembilan**: Gunung Angsi Forest Reserve [100, 101], Berembun Forest Reserve [101].

Unlike other *Rhinolophus* species, *R*. *luctus* sensu lato roosts either solitarily or in pairs often in caves, rock crevices, tree hollows and among tree roots, and has been recorded in primary and secondary forests [11, 14, 23].

***Rhinolophus luctoides*** Volleth, Loidl, Mayer, Yong, Müller and Heller, 2015

*Rhinolophus luctoides* Volleth, Loidl, Mayer, Yong, Müller and Heller, 2015: 4; Ulu Gombak, Selangor, MALAYSIA, 600 m (K. -G. Heller and M. Volleth, collector; SMF 87483) [3].

**Barcode Index Number:** There are no DNA barcodes recorded under this name on BOLD.

**Remarks:** *R*. *luctoides* and *R*. *morio* were previously synonymised under *R*. *luctus* but are distinct from *R*. *luctus* on the basis of molecular and morphological characters. *R*. *luctoides* has a larger ratio of lower toothrow length to mandible length and larger baculum length compared to *R*. *morio* [3].

**IUCN status:** Not Evaluated but Least Concern as *R*. *luctus*.

**Recorded at: Selangor**: 5 km north-east of the Ulu Gombak [3]; **Pahang**: Cameron Highland and Genting Highland [3]. Individuals were captured in selectively logged dipterocarp forest at elevations higher than 600 m and in montane forest [3]. See *R*. *morio* for records of *R*. *luctus*.

***Rhinolophus macrotis*** Blyth, 1844

Rhinolophus macrotis Blyth, 1844: 485; NEPAL (Brian Houghton Hodgson, presenter; BM(NH) 45.1.8.416) [160].

**Common English name:** Big-eared Horseshoe Bat

**Barcode Index Number:** DNA barcodes recorded as *R*. *macrotis* are associated with two BINs, BOLD:AAC2064 and BOLD:ACU9422, but there are no DNA barcodes from Peninsular Malaysia.

**Remarks:** The BIN, BOLD:AAC2064 includes DNA barcodes recorded as *R*. *macrotis* and *R*. *siamensis*, and both demonstrated very shallow genetic divergences [4] (S5 Fig). The BIN, BOLD:ACU9422 contains two DNA barcodes which are originally from GenBank and may be erroneous.

**IUCN status:** Least Concern

**Recorded at: Pahang**: Krau Wildlife Reserve [11, 41, 42], Gunung Benom [23], Pulau Tioman [79], Klau Besar [100]; **Perlis**: Wang Kelian State Park [50].

*R*. *macrotis* has been recorded in lowland and hill forests [11, 14, 23].

***Rhinolophus malayanus*** Bonhote, 1903

*Rhinolophus malayanus* Bonhote, 1903: 15; Biserat, Jalor, Patani, south THAILAND (Collector unknown: BM(NH) 1903.2.6.83) [164].

**Common English name:** Malayan Horseshoe Bat

**Barcode Index Number:** BOLD:AAA9603 (1 DNA barcode from Peninsular Malaysia; Fig 5)

**IUCN status:** Least Concern

**Recorded at: Perlis**: **Perak**: Bukit Jerneh Cave and Tumang Lembing Cave [30]; Wang Kelian State Park [50], Wang Tangga at Kaki Bukit [140]; **Kedah**: Kisap Forest Reserve in Pulau Langkawi [140].

*R*. *malayanus* roosts in limestone caves in colonies of hundreds of individuals [14].

***Rhinolophus marshalli*** Thonglongya, 1973

*Rhinolophus marshalli* Thonglongya, 1973: 590; foothills of Khao Soi Duo, Amphoe Pong Nam Ron, Chantthaburi, southeast THAILAND (Joe T. Marshall Jr. and Wandee Nong Ngok, collectors; TNRC 54–1669) [165].

**Common English name:** Marshall’s Horseshoe Bat

**Barcode Index Number:** DNA barcodes recorded as *R*. *marshalli* are associated with two BINs, BOLD:AAE7426; BOLD:ABZ6523, but there are no DNA barcodes from Peninsular Malaysia.

**IUCN status:** Least Concern

**Recorded at: Perlis**: Guar Jentik [166].

*R*. *marshalli* has been recorded in lowland and hill forests at elevation of 800 m, roosting in limestone caves [14].

***Rhinolophus pusillus*** Temminck, 1834

*Rhinolophus pusillus* Temminck, 1834: 29; Java, INDONESIA (Collector unknown; NMNL 35177, lectotype) [116].

**Common English name:** Least Horseshoe Bat

**Barcode Index Number:** DNA barcodes recorded as *R*. *pusillus* are associated with three BINs, (BOLD:AAA9397, BOLD:ABZ1016, and BOLD:ABZ2360), but there are no DNA barcodes from Peninsular Malaysia (S6 Fig).

**Remarks:** The BIN, BOLD:ABZ1016 contains DNA barcodes recorded as *R*. *pusillus* and *R*. *lepidus*. We suspect that the DNA barcodes recorded as *R*. *pusillus* (ABBSI244-10, ABBSI253-10, ABBSI263-10 and ABRVN310-06) are cases of mis-identification (see remarks on *R*. *lepidus*). Which BIN, if any, represents the valid *R*. *pusillus* remains to be determined.

**IUCN status:** Least Concern

**Recorded at: Kedah**: Ulu Muda Forest Reserve [57]; **Pahang**: Pulau Tioman (DWNP-M-08077, DWNP-M-08080, DWNP-M-08083); **Johor**: Gunung Ledang State Park (DWNP-M-08076, DWNP-M-08078, DWNP-M-08079, DWNP-M-08081, DWNP-M-08082); **Negeri Sembilan**: Berembun Forest Reserve [101].

*R*. *pusillus* roosts in caves, bamboo clumps and buildings and has been reported foraging in primary and secondary forests [14].

***Rhinolophus robinsoni*** Andersen, 1918

*Rhinolophus robinsoni* Andersen, 1918: 375; Khao Nawng, Bandon, THAILAND (Federated Malay States Museum, presenter; BM(NH) 1918.8.2.1) [117].

**Common English name:** Peninsular Horseshoe Bat

**Barcode Index Number:** There are no DNA barcodes recorded under this name on BOLD.

**Remarks:** *R*. *robinsoni* was previously considered to be conspecific with *R*. *megaphyllus* [9] but is now recognised as a distinct species [98]. Specimens recorded as *R*. *megaphyllus* from Peninsular Malaysia [46] should be updated to *R*. *robinsoni*.

**IUCN status:** Near Threatened

**Recorded at: Pahang**: Krau Wildlife Reserve [11], Fraser Hill [23, 140], Pulau Tioman [23], Kenong and Gunung Aais [100]; **Johor**: Pulau Aur and Pulau Pemanggil [23], Gunung Panti and Labis Forest Reserve [100]; **Perlis**: Wang Kelian State Park [50]; **Kelantan:** Gua Musang [62]; **Melaka**: Unspecified [68]; **Negeri Sembilan**: Gunung Angsi Forest Reserve [100]; **Perak**: Kledang Saiong Forest Reserve [100]; **Pulau Pinang**: Bukit Panchor [100].

Recorded as *R*. *megaphyllus* at **Perak**: Temengor Forest Reserve [46].

*R*. *robinsoni* inhabits forests primarily and has been recorded in lowland and hill forests roosting in rock crevices and palm leaves [11, 14].

***Rhinolophus sedulus*** Andersen, 1905

*Rhinolophus sedulus* Andersen, 1905: 247; Sarawak, MALAYSIA (A. R. Wallace, collector; Type was previously recorded as no.19 in Robert Fisher Tome’s private collection and is currently at BM(NH) as BM(NH) 7.1.1.292) [161].

**Common English name:** Lesser Woolly Horseshoe Bat

**Barcode Index Number:** DNA barcodes recorded as *R*. *sedulus* (BM141-03 and BM431-04) are not associated with any BIN due to their short sequence length (<500 bp). Both were collected in Peninsular Malaysia and share >99% similarity (Fig 5).

**IUCN status:** Near Threatened

**Recorded at: Pahang**: Krau Wildlife Reserve [11, 32], Kuala Tekah, [23], Kuala Atok, National Park [44], Bukit Ibam, Kemasul, Klau Besar and Gunung Aais [100]; **Negeri Sembilan**: Pasoh Forest Reserve, [45]; **Selangor**: Bukit Kutu Wildlife Reserve [51], Ulu Gombak [52, 54, 122], Air Hitam Forest Reserve [55], Semangkok Forest Reserve [101]; **Johor**: Endau-Kota Tinggi Forest Reserve [56], Gunung Panti [100]; **Kedah**: Ulu Muda Forest Reserve [57], Bukit Hijau [100]; **Perak**: Kledang Saiong Forest Reserve [100].

*R*. *sedulus* has been reported roosting in caves, fallen tree hollows, and bushes either individually or in pairs [11, 14, 23].

***Rhinolophus stheno*** Andersen, 1905

*Rhinolophus stheno* Andersen, 1905: 91, pl. 3; Selangor, MALAYSIA (H. N. Ridley, Esq., presenter; BM(NH) 98.3.13.1) [147].

**Common English name:** Lesser Brown Horseshoe Bat

**Barcode Index Number:** DNA barcodes recorded as *R*. *stheno* are associated with two BINs, BOLD:AAO1567 and BOLD:ABY7284, but there are no DNA barcodes from Peninsular Malaysia in these BINs. Two DNA barcodes recorded as *R*. *stheno* (BM504-04 and BM505-04) are from Peninsular Malaysia but are not placed in any BIN due to short sequence length (<500bp) Based on our NJ analysis, neither of the Peninsular Malaysia barcodes are associated with BOLD:AAO1567 or BOLD:ABY7284 (Fig 5).

**Remarks:** *R*. *microglobosus* was described as a subspecies of *R*. *stheno* based on its smaller skull and globular anterior median rostral swellings [167]. The taxa were later found to be morphometrically and acoustically distinct, and *R*. *microglobosus* was consequently raised as a distinct species with a distribution covering Thailand, Myanmar, Cambodia, Vietnam and Laos whereas *R*. *stheno* is restricted to southern Thailand, Peninsular Malaysia and central Vietnam [168]. Therefore, DNA barcodes recorded as *R*. *stheno* associated with the BIN, BOLD:AAO1567 may represent *R*. *microglobosus*, and DNA barcodes, BM504-04 and BM505-04 [4] may represent the *R*. *stheno* sensu stricto as they were collected at Kuala Lompat, Pahang, close to the type locality. The BIN, BOLD:ABY7284 which contains DNA barcodes recorded as *R*. *stheno* and *R*. *shameli* may be erroneous (Fig 5).

**IUCN status:** Least Concern

**Recorded at: Pahang**: Krau Wildlife Reserve [11, 42], Tasik Chini [43], Lata Bujang Forest Reserve [56], Cameron Highland [60], Bukit Ibam, Kemasul, Jengka, Pulau Tioman [79], Klau Besar, Kenong and Gunung Aais [100]; **Pulau Pinang**: Bukit Panchor [23, 100]; **Perak**: Temengor Forest Reserve [46, 47, 111], Royal Belum State Park [66], Kledang Saiong Forest Reserve [101]; **Perlis**: Wang Kelian State Park [50]; **Selangor**: Bukit Kutu Wildlife Reserve [51], Ulu Gombak [54, 101], Semangkok Forest Reserve [101]; **Kedah**: Ulu Muda Forest Reserve [57, 100], Bukit Hijau [100]; **Kelantan**: Air Panas-Gua Musang [61], Gua Musang [62], Gunung Stong State Park [100]; **Johor**: Gunung Panti and Labis Forest Reserve [100]; **Negeri Sembilan**: Gunung Angsi Forest Reserve [100, 101], Berembun Forest Reserve [101]; **Terengganu**: Gunung Tebu Forest Reserve [101].

*R*. *stheno* roosts in limestone caves and sometimes in smaller colonies in rock crevices and tree hollows [11, 14]. Individuals have been reported roosting with *R*. *lepidus* [11].

***Rhinolophus trifoliatus*** Temminck, 1834

*Rhinolophus trifoliatus* Temminck, 1834: 24, pl. 1 (and 1835: 27, pl. 31); Bantam, west Java, INDONESIA (Collector unknown; NMNL 35194) [116].

**Common English name:** Trefoil Horseshoe Bat

**Barcode Index Number:** BOLD:AAB6408 (10 DNA barcodes from Peninsular Malaysia; Fig 5)

**IUCN status:** Least Concern

**Recorded at: Pahang**: Krau Wildlife Reserve [11, 41, 42], Tasik Chini [43], Kuala Atok, National Park [44], Tasek Bera Forest Reserve, Lata Bujang Forest Reserve and Fraser Hill Forest Reserve [56], Gunung Tahan [92], Bukit Ibam, Kemasul, Jengka, Klau Besar and Gunung Aais [100]; **Selangor**: Ulu Gombak [40, 54, 101], Bangi Forest Reserve [41], Bukit Kutu Wildlife Reserve [51], Air Hitam Forest Reserve [55], Sungai Dusun Forest Reserve [56], Semangkok Forest Reserve [101]; **Negeri Sembilan**: Pasoh Forest Reserve [45], Gunung Angsi Forest Reserve [100, 101], Berembun Forest Reserve [101]; **Perak**: Temengor Forest Reserve [46, 47, 111], Royal Belum State Park [48, 66], Temenggor Lake [69], Kledang Saiong Forest Reserve [100, 101]; **Perlis**: Wang Kelian State Park [50]; **Johor**: Endau-Kluang Forest Reserve and Endau-Kota Tinggi Forest Reserve [56], Gunung Panti and Labis Forest Reserve [100]; **Kedah**: Ulu Muda Forest Reserve [57, 100]; **Kelantan**: Air Panas-Gua Musang [61]; **Melaka**: Sungai Udang Forest Reserve [68]; **Terengganu**: Tasik Kenyir [69], Gunung Tebu Forest Reserve [101]; **Pulau Pinang**: Bukit Panchor [100].

*R*. *trifoliatus* roosts solitarily under leaves of palms, rattan and small trees, and has been recorded in mangroves, and primary and secondary forests at all elevations [11, 14, 23].

### Family: Vespertilionidae (subfamily: Kerivoulinae)

***Kerivoula hardwickii*** [Hordfield, 1824]

*Vespertilio hardwickii* Horsfield, 1824: part 8; Java, INDONESIA (Collector unknown; Type: BM(NH) 79.11.29.181) [102].

*Kerivoula hardwickii* [8].

**Common English name:** Hardwicke's Woolly Bat

**Fig 6:**
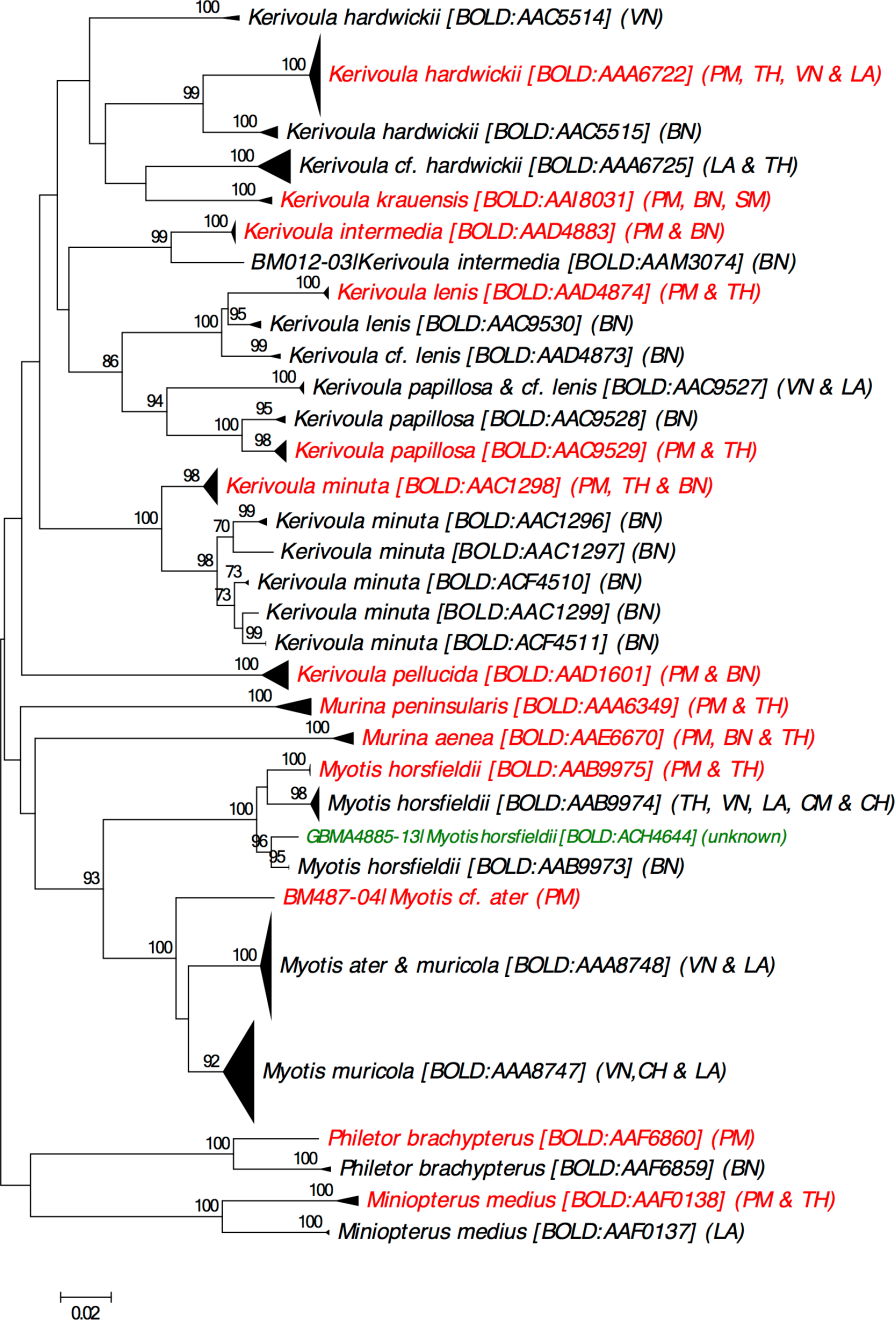
Neighbour-joining tree showing all available DNA barcodes for species in family Vespertilionidae reported from Peninsular Malaysia. The percentage of pseudoreplicate trees (≥70%) in which the DNA barcodes clustered together in the bootstrap test (500 pseudoreplicates) are shown above the branches. Abbreviation as follows: PM = Peninsular Malaysia, VN = Vietnam, BN = Borneo (including Sabah & Sarawak of East Malaysia, Brunei and Kalimantan Indonesia), TH = Thailand, LA = Laos, SM = Sumatera Indonesia, JV = Java Indonesia, CH = China, CM = Cambodia.

**Barcode Index Number:** BOLD:AAA6722 (5 DNA barcodes from Peninsular Malaysia; Fig 6)

**Remarks:** DNA barcodes recorded as *K*. *hardwickii* are associated with four BINs, BOLD:AAA6722, BOLD:AAA6725, BOLD:AAC5514 and BOLD:AAC5515. DNA barcodes from Peninsular Malaysia, Thailand, Vietnam and Laos formed a mainland group, whereas DNA barcodes from Malaysian Borneo and Kalimantan, Indonesia formed a Bornean group (Fig 6). Francis et al. [2] suggested that *K*. *hardwickii* is a species complex based on the analysis of COI mtDNA. However, Khan et al. [169] recognised only a single form across Malaysia (Peninsular and Borneo) and suggested that the Bornean form is a result of chromosomal polymorphism. Douangboubpha et al. [170] reported that specimens from Thailand referred as *K*. *hardwickii* can be divided into two morphotypes: “flat” skulls and “domed” skulls. Although the specimens with “flat” skulls did not show variation in size and morphology, they were closely clustered into two clades (*K*. *hardwickii* A and *K*. *hardwickii* B) 2.14% divergent in COI mtDNA; whereas specimens with “domed” skulls showed variation in size and morphology but were closely clustered together in a COI analysis (*K*. *hardwickii* C). “Domed” skulls were 16.37% and 20.02% divergent to *K*. *hardwickii* A and *K*. *hardwickii* B in COI mtDNA respectively [170].

There are no subspecies recognised currently [98] contradicting older literature. Ellerman and Morrison-Scott [138] recognised four subspecies: *hardwickii* (type locality: Java), *depressa* (type locality: southern Burma = Myanmar), *crypta* (type locality: southern India), and *malpasi* (type locality: Sri Lanka) whereas Hill [171] recognised five, including *engana* (type locality: southwest of Sumatra). The names *hardwickii* and *depressa* were suggested for the specimens from Thailand with “domed” and “flat” skulls respectively, but further research is required to assign Linnaean names conclusively [170]. Whether the four BINs in our NJ tree (Fig 6) represent the four subspecies remains to be determined. We tentatively retained the name *K*. *hardwickii* in this checklist pending further research.

**IUCN status:** Least Concern

**Recorded at: Kelantan**: Ulu Kelantan [23], Gunung Stong State Park [100], Air Panas-Gua Musang, [61], Gua Musang [62]; **Perak**: Temengor Forest Reserve [46, 47, 111], Royal Belum State Park [66], Kledang Saiong Forest Reserve [100, 101]; **Perlis**: Wang Kelian State Park [50]; **Johor**: Endau-Kluang Forest Reserve [56], Gunung Panti and Labis Forest Reserve [100]; **Melaka**: Sungai Udang Forest Reserve [68]; **Pahang**: Bukit Ibam, Klau Besar, and Gunung Aais [100]; **Pulau Pinang**: Bukit Panchor [100]; **Negeri Sembilan**: Gunung Angsi Forest Reserve [100, 101], Berembun Forest Reserve [101]; **Selangor**: Semangkok Forest Reserve and Ulu Gombak [101]; **Terengganu**: Gunung Tebu Forest Reserve [101].

*K*. *hardwickii* has been reported roosting in tree hollows, among clumps of dead leaves, and in dead and broken bamboo stems [14, 23].

***Kerivoula krauensis*** Francis, Kingston and Zubaid, 2007

*Kerivoula krauensis* Francis, Kingston and Zubaid, 2007: 3; Kuala Lompat, Krau Wildlife Reserve, Pahang, MALAYSIA, 3° 43’ N 102° 10’ E (Charles M. Francis, collector; BM(NH) 1999.294) [2].

**Common English name:** Krau Woolly Bat

**Barcode Index Number:** BOLD:AAI8031 (1 DNA barcode from Peninsular Malaysia; Fig 6)

**IUCN status:** Data Deficient. This species is vulnerable to the rapid loss of lowland rainforest [14].

**Recorded at: Pahang**: Krau Wildlife Reserve [2, 14]; **Terengganu:** Sekayu Recreational Forest [172].

*K*. *krauensis* has been recorded in primary lowland dipterocarp forest [2] and its roosting ecology remains unknown though it has not been recorded in caves [14].

***Kerivoula intermedia*** Hill and Fancis, 1984

*Kerivoula intermedia* Hill and Fancis, 1984: 323; Lumerau, Sabah, Borneo, MALAYSIA 5°12’N, 118°52’E (Charles M. Francis, collector; BM(NH) 1983.356) [173].

**Common English name:** Small Woolly Bat

**Barcode Index Number:** BOLD:AAD4883 (5 DNA barcodes from Peninsular Malaysia; Fig 6)

**Remarks:** DNA barcodes recorded as *K*. *intermedia* are associated with two BINs, BOLD:AAD4883, and BOLD:AAM3704. The BIN, BOLD:AAD4883 contains DNA barcodes from Peninsular Malaysia and Sarawak, Borneo while BIN, BOLD:AAM3074 contains only a single DNA barcode (BM012-03) from Sabah, Borneo (Fig 6). Whether the DNA barcode, BM012-03 represents a cryptic species, a case of mis-identification, or a case of high intraspecific variation remains to be determined.

**IUCN status:** Near Threatened

**Recorded at: Pahang**: Krau Wildlife Reserve [11, 32, 41], Tasik Bera Forest Reserve [56], Bukit Ibam, Kenong and Gunung Aais [100], Tekam Forest Reserve [173]; **Negeri Sembilan**: Pasoh Forest Reserve [45], Gunung Angsi Forest Reserve and Berembun Forest Reserve [101]; **Perak**: Royal Belum State Park [48, 66], Kledang Saiong Forest Reserve [100, 101], Temengor Forest Reserve [111]; **Perlis**: Wang Kelian State Park [50]; **Selangor**: Air Hitam Forest Reserve [55], Semangkok Forest Reserve [101]; **Johor**: Endau-Kota Tinggi Forest Reserve [56], Gunung Panti and Labis Forest Reserve [100]; **Kelantan**: Air Panas-Gua Musang [61], Gunung Stong State Park [100]; **Melaka**: Sungai Udang Forest Reserve [68]; **Terengganu**: Gunung Tebu Forest Reserve [101], Sungei Kelembang at Ulu Setiu in Besut [173].

The roosting ecology of *K*. *intermedia* remains unknown but the species has been recorded in the understory of lowland forest [11, 14].

***Kerivoula minuta*** Miller, 1898

*Kerivoula minuta* Miller, 1898: 321; Lay Song Hong, Trang, south THAILAND (Dr. W. L. Abbott, collector; USNM 83547) [174].

**Common English name:** Least Woolly Bat

**Barcode Index Number:** BOLD:AAC1298 (9 DNA barcodes from Peninsular Malaysia; Fig 6)

**Remarks:** DNA barcodes recorded as *K*. *minuta* are associated with six BINs, BOLD:AAC1296, BOLD:AAC1297, BOLD:AAC1298, BOLD:AAC1299, BOLD:ACF4510, and BOLD:ACF451 (Fig 6). Khan [175] reported 4.44% of divergence in cytochrome *b* mtDNA between *K*. *minuta* from Peninsular Malaysia and Malaysian Borneo (Sabah and Sarawak) with no shared haplotypes. Our NJ analysis also showed a divergence between *K*. *minuta* from Peninsular Malaysia and Borneo (Fig 6). The taxon occurring in Peninsular Malaysia represents *K*. *minuta* based on the type locality.

**IUCN status:** Near Threatened

**Recorded at: Johor**: Endau Rompin National Park (BM422-04 and ABRSS347-06 [4]), Gunung Panti and Labis Forest Reserve [100]; **Selangor**: Ulu Gombak and Hulu Langat [32]; **Pahang**: Krau Wildlife Reserve and Lakum [32], Kuala Atok, National Park [32, 44], Bukit Ibam, Kenong and Gunung Aais [100]; **Perak**: Maxwell Hill [32], Temengor Forest Reserve [46, 47], Royal Belum State Park [66]; **Kedah**: Bukit Hijau and Ulu Muda Forest Reserve [32, 57, 100]; **Kelantan**: Gua Musang [61, 62]; **Negeri Sembilan**: Gunung Angsi Forest Reserve [100].

*K*. *minuta* has been recorded in the understory of lowland forests and disturbed areas [14].

#### *Kerivoula papillosa* and *K*. *lenis*

*K*. *lenis* is closely associated with *K*. *papillosa* [9] but the former has a smaller skull and smaller teeth, shorter muzzle and narrower palate [169, 176]. The two species are 10.85% divergent in cytochrome *b* mtDNA and possess unique karyotypic characters: *K*. *papillosa* has a diploid number of chromosomes = 38 and fundamental number = 54 whereas *K*. *lenis* has a diploid number of chromosomes = 38 and fundamental number = 52 [32, 175]. Analyses of COI mtDNA by Francis et al. [4] suggested that there are at least four distinct clusters among specimens recorded as *K*. *papillosa* and *K*. *lenis*. Douangboubpha et al. [170] reported that specimens from Thailand referred as *K*. *papillosa* represent five morphological forms but only three distinct clusters based on COI mtDNA analyses.

***Kerivoula papillosa*** Temminck, 1840

*Kerivoula papillosa* Temminck, 1840: 220, PL. 55; Bantam, west Java (restricted by Tate 1940), INDONESIA (Collector unknown; Type unknown) [82].

*Kerivoula malayana* Chasen, 1940: 55; Ginting Bedai, Selangor-Pahang, MALAYSIA, 2300ft (Collector unknown; BM(NH) 1947.1483) [8].

*Kerivoula papillosa malayana* [23]

**Common English name:** Papillose Woolly Bat

**Barcode Index Number:** BOLD:AAC9529 (8 DNA barcodes from Peninsular Malaysia; Fig 6)

**Remarks:** *K*. *malayana* was described based on a specimen collected at the Selangor-Pahang border in Peninsular Malaysia [8]. Two forms of *K*. *papillosa* which are different in forearm length and acoustic characters were reported from Krau Wildlife Reserve, Peninsular Malaysia [177]. However, Douangboubpha et al. [170] reported five morphological forms within three distinct clusters based on COI mtDNA (*K*. *papillosa* A, B and C) in Thailand. *K*. *papillosa* A corresponds with *K*. *p*. *malayana* based on the size a larger skull and higher braincase and is 6.97% divergent from *K*. *papillosa* B which comprises two morphological forms. The smaller *K*. *papillosa* B (forearm length: 42.1–42.3 mm and length of skull: 17.0–17.1 mm) and the larger *K*. *papillosa* B (forearm: 39.4–40.2 mm and length of skull: 16.6–17.0 mm) are only 1.99% divergent and may or may not represent a further undescribed species. *K*. *papillosa* C is only 0.55% divergent from *K*. *lenis* collected in Peninsular Malaysia with morphological variation, and is 13.06% and 14.86% divergent from *K*. *papillosa* A and B respectively (see Fig 9 in [170]).

Our NJ analysis (Fig 6) revealed three clusters of DNA barcodes recorded as *K*. *papillosa* corresponding to three BINs, BOLD:AAC9527, BOLD:AAC9528 and BOLD:AAC9529. It is likely that BIN, BOLD:AAC9529 (as *K*. *papillosa* Small in [169] and as *K*. *papillosa* A [170]) with DNA barcodes recorded as *K*. *papillosa* and *K*. cf. *papillosa* represent *K*. *p*. *malayana* based on type locality. We conservatively retained *K*. *papillosa* in our checklist and propose further research be conducted to address the suggestion that “*malayana*” to recognised as a distinct species.

**IUCN status:** Least Concern

**Recorded at: Pahang**: Krau Wildlife Reserve [11, 32, 41], Kuala Atok, National Park [44], Tasik Bera Forest Reserve and Fraser Hill Forest Reserve [56], Bukit Ibam, Kemasul, Jengka, Klau Besar, Kenong and Gunung Aais [100]; **Selangor**: Bangi Forest Reserve [41], Bukit Kutu Wildlife Reserve [51], Air Hitam Forest Reserve [55], Sungai Dusun Forest Reserve [56], Semangkok Forest Reserve [101], Ulu Gombak [120]; **Negeri Sembilan**: Pasoh Forest Reserve [45], Gunung Angsi Forest Reserve [100, 101], Berembun Forest Reserve [101]; **Perak**: Temengor Forest Reserve [46, 47, 111], Royal Belum State Park [48, 66], Kledang Saiong Forest Reserve [100, 101]; **Perlis**: Wang Kelian State Park [50]; **Johor**: Endau-Kota Tinggi Forest Reserve [56], Gunung Panti and Labis Forest Reserve [100]; **Kedah**: Ulu Muda Forest Reserve [57, 100]; **Kelantan**: Gua Musang [62], Gunung Stong State Park [100]; **Melaka**: Sungai Udang Forest Reserve [68]; **Pulau Pinang**: Bukit Panchor [100]; **Terengganu**: Gunung Tebu Forest Reserve [101].

*K*. *papillosa* roosts in pairs or small groups, with males tending to roost solitarily [11, 23]. The species has been recorded roosting in dead or broken bamboo stems and cavities in live standing trees [11, 14, 23]. The range for *K*. *papillosa* may be very small based on the high recapture rate in Krau Wildlife Reserve [11].

***Kerivoula lenis*** Thomas, 1916

*Kerivoula lenis* Thomas, 1916: 416; Calcutta, Bengal, INDIA (J. T. Pearson, presenter; BM(NH) 1879.11.21.126) [178].

**Common English name:** Indian Woolly Bat

**Barcode Index Number:** BOLD:AAD4874 (3 DNA barcodes from Peninsular Malaysia; Fig 6)

**Remarks:** *K*. *lenis* was previously considered as a subspecies of *K*. *papillosa* [171] but was later raised as a distinct species [98,176]. Douangboubpha et al. [170] reported three distinct clusters of *K*. *papillosa* based on NJ analyses at COI mtDNA (*K*. *papillosa* A, B and C) in Thailand of which *K*. *lenis* from Peninsular Malaysia (BIN, BOLD:AAD4874) is clustered with *K*. *papillosa* C. *K*. *lenis* from Peninsular Malaysia has been reported to be 5.33% divergent from *K*. *lenis* from Borneo and >14% divergent from *K*. cf. *lenis* from Laos [4, 169, 170]. Whether the taxon occurring in Peninsular Malaysia represents *K*. *lenis* sensu stricto remains to be determined due to the lack of comparative materials from the type locality, India [170].

Our NJ analysis (Fig 6) revealed three clusters of DNA barcodes recorded as *K*. *lenis* and *K*. cf. *lenis* associated with three BINs, BOLD:AAC9530, BOLD:AAD4873 and BOLD:AAD4874.

**IUCN status:** Least Concern

**Recorded at: Pahang**: Krau Wildlife Reserve [32]; **Negeri Sembilan**: Pasoh Forest Reserve (specimen BM(NH) 1988.46).

*K*. *lenis* has been recorded in understory of forest [14].

***Kerivoula pellucida*** [Waterhouse, 1845]

*Vespertilio pellucidus* Waterhouse, 1845: 6; PHILLIPINES (H. Cuming, Esq.; Type unknown) [179].

*Kerivoula pellucida* [180].

**Common English name:** Clear-winged Woolly Bat

**Barcode Index Number:** BOLD:AAD1601 (8 DNA barcodes from Peninsular Malaysia; Fig 6)

**IUCN status:** Near Threatened

**Recorded at: Pahang**: Krau Wildlife Reserve [11], Kuala Atok, National Park [44], Tasik Bera Forest Reserve and Fraser Hill Forest Reserve [56], Bukit Ibam, Kemasul, Jengka, Kenong and Gunung Aais [100]; **Negeri Sembilan**: Pasoh Forest Reserve [45], Gunung Angsi Forest Reserve [100, 101], Berembun Forest Reserve [101]; **Perak**: Temengor Forest Reserve [46, 47, 48], Royal Belum State Park [66], Kledang Saiong Forest Reserve [100]; **Perlis**: Wang Kelian State Park [50]; **Johor**: Endau-Kluang Forest Reserve [56], Gunung Panti [100]; **Kelantan**: Air Panas-Gua Musang [61], Gunung Stong State Park [100]; **Kedah**: Bukit Hijau [100]; **Selangor**: Semangkok Forest Reserve and Ulu gombak [101]; **Terengganu**: Gunung Tebu Forest Reserve [101].

*K*. *pellucida* has been reported foraging in understory of tall forests with dense vegetation and roosting in clumps of dried leaves [11, 14, 23]. Captured individuals were reported roosting in tight clusters in harp traps, suggesting social bonds [11].

***Kerivoula picta*** [Pallas, 1767] (?)

*Vespertilio pictus* Pallas, 1767: 7; probably Ternate Island, north Moluccas, INDONESIA (Collector unknown; Type unknown) [181].

*Kerivoula picta* [133].

**Common English name:** Painted Woolly Bat

**Barcode Index Number:** DNA barcodes recorded as *K*. *picta* are associated with the BIN, BOLD:AAX0264, but there are no DNA barcodes from Peninsular Malaysia.

**IUCN status:** Least Concern

**Recorded at: Pulau Pinang**: Unspecified [133].

We could not find recent locality reports for *K*. *picta* in Peninsular Malaysia although the species is thought to occur in Peninsular Malaysia [1, 6, 9, 10, 14, 23]. This species has been recorded from Thailand [74] and may be restricted to northern Peninsular Malaysia. *K*. *picta* has been reported roosting among dead leaves of trees and bananas [14].

***Kerivoula whiteheadi*** Thomas, 1894 (?)

*Kerivoula whiteheadi* Thomas, 1894: 460; Molino, Isabella, northeast Luzaon Island, PHILLIPINES (J. Whitehead, collector; BM(NH) 1894.10.9.2) [182].

**Common English name:** Whitehead's Woolly Bat

**Barcode Index Number:** There are no DNA barcodes recorded under this name on BOLD.

**IUCN status:** Least Concern

**Remarks:** Chasen [8] listed *K*. *whiteheadi* as *Kerivoula bicolor* (now *Kerivoula whiteheadi bicolor*) in his “Handlist of Malaysian Mammals”.

**Recorded at:** The holotype of *K*. *whiteheadi bicolor* (BM(NH) 3.2.6.91) which was collected in Biserat, Jalor = Yala, Malay Peninsula, which is now the southern tip of Thailand, is the only record from the mainland [14, 74]. *K*. *whiteheadi* may be expected to occur in Peninsular Malaysia [6, 8, 9, 23] based on the type locality but is yet to be documented [169].

*K*. *whiteheadi* has been recorded in secondary forests, shrubs and open grasslands, and found roosting in small groups of twenty to thirty individuals among dead leaves by a river [14].

***Phoniscus atrox*** Miller, 1905

*Phoniscus atrox* Miller, 1905: 230; vicinity of the Kateman River, east Sumatra, INDONESIA (Dr. W. L. Abbott, collector; USNM 123141) [183].

**Common English name:** Lesser Groove-toothed Bat

**Barcode Index Number:** There are no DNA barcodes recorded under this name on BOLD.

**IUCN status:** Near Threatened

**Recorded at: Pahang**: Krau Wildlife Reserve [11, 41, 42], Bukit Ibam, Kemasul and Gunung Aais [100]; **Terengganu**: Pasir Raja-Dungun [15], Gunung Tebu Forest Reserve [101]; **Selangor**: Ulu Gombak [23, 52, 54]; **Negeri Sembilan**: Pasoh Forest Reserve [45]; **Perak**: Temengor Forest Reserve [46, 47, 111], Royal Belum State Park [66]; **Kelantan**: Air Panas-Gua Musang [61]; **Johor**: Labis Forest Reserve [100].

*P*. *atrox* has been recorded in primary lowland forests and disturbed areas near primary forest, and found roosting in abandoned hanging bird nests [11, 14].

***Phoniscus jagorii*** [Peters, 1866]

*Vespertilio (Kerivoula) jagorii* Peters, 1866: 399; Samar Island, PHILLIPINES (Collector unknown; Type unknown) [184].

*Phoniscus jagorii* [11].

**Common English name:** Greater Groove-toothed Bat

**Barcode Index Number:** DNA barcodes recorded as *P*. *jagorii* are associated with the BIN, BOLD:AAC4331, but there are no DNA barcodes from Peninsular Malaysia.

**IUCN status:** Least Concern

**Recorded at: Pahang**: Krau Wildlife Reserve [11].

*P*. *jagorii* is rare in understorey of forest and has been recoded in primary lowland forests [11, 14].

### Family: Vespertilionidae (subfamily: Miniopterinae)

***Miniopterus magnater*** Sanborn, 1931

*Miniopterus schreibersii magnater* Sanborn, 1931: 26; Marienburg, 40 miles up the Sepik River, PAPUA NEW GUINEA (Frank C. Wonder, collector; FMNH 31802) [185].

*Miniopterus magnater* [9].

**Common English name:** Large Bent-winged Bat

**Barcode Index Number:** DNA barcodes recorded as *M*. *magnater* are associated with the BIN, BOLD:AAA9957, but there are no DNA barcodes from Peninsular Malaysia.

**IUCN status:** Least Concern

**Recorded at: Terengganu**: Bukit Dendong [97]; **Pahang**: National Park (DWNP-M-07512).

*M*. *magnater* is a cave dweller and has been recorded near streams and small bodies of water [14].

***Miniopterus medius*** Thomas and Wroughton, 1909

*Miniopterus medius* Thomas and Wroughton, 1909: 382; Kalipoetjang, Tji-Tandoei River, west Java, INDONESIA (G. C. Shortridge, collector; BM(NH) 1909.1.5.464) [186].

**Common English name:** Medium Bent-winged Bat

**Barcode Index Number:** BOLD:AAF0138 (1 DNA barcode from Peninsular Malaysia; Fig 6)

**Remarks:** DNA barcodes recorded as *M*. *medius* are associated with two BINs, BOLD:AAF0137, and BOLD:AAF0138. The BIN, BOLD:AAF0138 contains the only DNA barcode from Peninsular Malaysia (ABBSI031-04) and unidentified DNA barcodes from Thailand. None of the DNA barcodes were collected near the type locality. We found an 8.1% divergence between the two BINs (Fig 6). No subspecies is described for the species at the moment.

**IUCN status:** Least Concern

**Recorded at: Pahang**: Krau Wildlife Reserve [11], Panching and Fraser Hill [23], Bukit Cheras [140]; **Perak**: Maxwell Hill and Gunong Pondok [23]; **Johor**: Kaban Island [23]; **Selangor**: Ulu Gombak [53]; **Terengganu**: Bukit Dendong [97].

*M*. *medius* roosts in caves and inhabits primary lowland, hill and montane forests [11, 14, 23].

***Miniopterus schreibersii*** [Kuhl, 1817]

*Vespertilio schreibersii* Kuhl, 1817: 185; ‘Columbäzar Höhle’, R Danube, ROMANIA (Collector unknown; Type unknown) [187]

*Miniopterus schreibersii blepotis* [9, 23].

*Miniopterus fuliginosus* [33].

**Common English name:** Common Bent-winged Bat

**Barcode Index Number:** DNA barcodes recorded as *M*. *schreibersii* are associated with four BINs, BOLD:AAC3658, BOLD:ACE8769, BOLD:AAX4032 and BOLD:AAA995, but there are no DNA barcodes from Peninsular Malaysia.

**Remarks:** Tian et al. [188] discovered a large divergence in cytochrome *b* mtDNA among specimens of *M*. *schreibersii* from Europe, Asia and Australia, congruent with previous studies [189, 190]. The taxonomy of *M*. *schreibersii* was then revised based on molecular and geographic characters resulting in *M*. *schreibersii* sensu stricto in Europe, *M*. *oceanensis* in Australia and *M*. *fuliginosus* in Asia [188]. However, Tian et al. [188] only included specimens from Japan and China to represent “Asia”. Consequently, we retained the name *M*. *schreibersii* in our checklist following Kingston et al. [11] and Francis [14] pending further research.

**IUCN status:** Near Threatened

**Recorded at: Pahang**: Krau Wildlife Reserve [11], Fraser Hill [23]; **Perlis**: Kaki Bukit [23]; **Perak**: Maxwell Hill [23], Temengor Forest Reserve [46, 47]; **Selangor**: Bukit Kutu Wildlife Reserve [51], Ulu Gombak [54]; **Kedah**: Ulu Muda Forest Reserve [57]; **Melaka**: Sungai Udang Forest Reserve [68].

*M*. *schreibersii* has been recorded in primary hill and montane forests [11], and roosts in caves in large colonies, sometimes with other *Miniopterus* bats [14, 23].

### Family: Vespertilionidae (subfamily: Murininae)

***Harpiocephalus harpia*** [Temminck, 1840]

*Vespertilio harpia* Temminck, 1840: 219, pls. 55; Southeast side of Mountain Gede, Java, INDONESIA (Collector unknown; Type unknown) [82].

*Harpiocephalus harpia* [191].

**Common English name:** Hairy-winged Bat

**Barcode Index Number:** DNA barcodes recorded as *H*. *harpia* are associated with BIN, BOLD:AAB5424, but there are no DNA barcodes from Peninsular Malaysia.

**Remarks:** *H*. *mordax* was once considered a subspecies of *H*. *harpia* [138] but was later recognised as a distinct species having “a more robust skull and larger teeth” compared to *H*. *harpia* [173]. Two male and three female specimens from Java recorded as *H*. *harpia* were later re-examined, and the degree of dimorphism observed among the specimens was small when compared to the differences observed in rostral and tooth size between *H*. *harpia* and *H*. *mordax* [9]. Matveev [192] noted that all specimens of *H*. *mordax* used in earlier studies are females and a molecular analysis of a male *“harpia”* and a female “*mordax”* from Cambodia indicated that the specimens were conspecific, consequently eliminating the occurrence of *H*. *mordax* in Cambodia. Two female specimens from Peninsular Malaysia (field ID.: CMF930806.7 and CMF930807.2) were identified as “*H*. *mordax*” based on their broader skull and large teeth by Francis [71]. Francis later stated that *H*. *harpia* is the only species that occurs in Southeast Asia with sexual dimorphism in size [14]. Following the current consensus, all records of *H*. *mordax* from Peninsular Malaysia should be updated to *H*. *harpia*.

**IUCN status:** Least Concern

**Recorded at: Pahang:** Fraser Hill Forest Reserve [56].

Previously recorded as *H*. *mordax* at: **Pahang**: Krau Wildlife Reserve [11], National Park [193]; **Perak**: Temengor Forest Reserve [46, 47, 71].

The roosting ecology of *H*. *harpia* remains unknown due to its rarity but the species has been recorded in forests with hilly terrains [11].

***Murina aenea*** Hill, 1964

*Murina aenea* Hill, 1964: 57, pls 54, 55; Ulu Chemperoh, near Janda Baik, Bentong District, Pahang, MALAYSIA, c. 3°18’N, 101°50’E, 2000 ft (Collector unknown; BM(NH) 1964.770) [194].

**Common English name:** Bronzed Tube-nosed Bat

**Barcode Index Number:** BOLD:AAE6670 (2 DNA barcodes from Peninsular Malaysia; Fig 6)

**IUCN status:** Vulnerable

**Recorded at: Pahang**: Krau Wildlife Reserve [11], Bentong [23], Bukit Ibam and Klau Besar [100], Ulu Chemperoh [192]; **Terengganu**: Pasir Raja, Dungun [15]; **Perak**: Temengor Forest Reserve [46, 47]; **Johor**: Gunung Panti [100]; **Kedah:** Bukit Hijau [100]; **Selangor**: Ulu Gombak [173].

The roosting ecology of *M*. *aenea* remains unknown but the species has been recorded in lowland and hill dipterocarp forests [11, 14, 23].

***Murina peninsularis*** Hill, 1964

*Murina cyclotis* peninsularis Hill, 1964: 55; Ulu Chemperoh, near Janda Baik, Bentong District, Pahang, MALAYSIA (Collector unknown; BM(NH) 1964.771) [194].

*Murina peninsularis* [34].

**Common English name:** Peninsular Tube-nosed Bat

**Barcode Index Number:** BOLD:AAA6349 (2 DNA barcodes from Peninsular Malaysia; Fig 6)

**Remarks:** Three subspecies were previously described under *M*. *cyclotis* based on their geographical distributions: *M*. *c*. *cyclotis* from northeast India to Vietnam, the slightly darker and duller *M*. *c*. *eileenae* from Sri Lanka, and *M*. *c*. *peninsularis* from Peninsular Thailand to Malaysia and Indonesia [9, 34]. However, the consistent medium-large body size and genetic distance in COI mtDNA support the recognition of the Sundaic *M*. *c*. *peninsularis* as a distinct species [4, 33, 34]. Therefore, all records of *M*. *cyclotis* from Peninsular Malaysia should be updated to *M*. *peninsularis* [34].

**IUCN status:** Not Evaluated but Least Concern as *M*. *cyclotis*.

**Recorded at: Perlis**: Wang Kelian State Park [51]; **Kelantan:** Loging Highlands [64]; **Pahang**: Ulu Chemperoh, near Janda Baik [194].

Recorded as *M*. *cyclotis* at: **Pahang**: Krau Wildlife Reserve [11, 42], Kuala Atok-National Park [44], Cameron Highland [60], Bukit Ibam, Klau Besar and Kenong [100]; **Negeri Sembilan**: Pasoh Forest Reserve [45], Gunung Angsi Forest Reserve and Berembun Forest Reserve [101]; **Perak**: Temengor Forest Reserve [46, 111], Royal Belum State Park [66], Kledang Saiong Forest Reserve [100, 101]; **Perlis**: Wang Kelian State Park [50]; **Selangor**: Bukit Kutu Wildlife Reserve [51]; **Kelantan**: Lojing Highlands [62], Gunung Stong State Park [67]; **Johor**: Labis Forest Reserve [100].

*M*. *peninsularis* has been recoded in wide variety of forest types [14] but its roosting ecology remains unknown [11].

***Murina huttoni*** [Peters, 1872]

*Harpyiocephalus huttonii* Peters, 1872: 257; Dehra Dun, Kumaon, northwest INDIA (Collector unknown; BM(NH) 1879.11.21.685) [195].

*Murina huttoni* [23].

**Common English name:** Hutton's Tube-nosed Bat

**Barcode Index Number:** DNA barcodes of *M*. *huttoni* are associated with three BINs, BOLD:AAC6107, BOLD:AAC6108 and BOLD:AAC6109, but there are no DNA barcodes from Peninsular Malaysia.

**IUCN status:** Least Concern

**Remarks:** Francis and Eger [33] commented that *M*. *huttoni* may be the only *Murina* species that occurs in both Peninsular Malaysia and Indo-Burma after *M*. *peninsularis* was separated from *M*. *cyclotis*. The large divergence among the DNA barcodes in our NJ tree suggest that *M*. *huttoni* is also a species complex (S7 Fig). Simmons [98] recognises two subspecies: *M*. *h*. *huttoni*. (type locality: India) and *M*. *h rubella* (type locality: Fokien, China). Whether the *M*. *huttoni* in Peninsular Malaysia represents either of these subspecies remains to be determined [33].

**Recorded at: Pahang**: Gunong Benom in Krau Wildlife Reserve [23]. The only specimens of *M*. *huttoni* from Peninsular Malaysia was trapped at 1400 m [23].

***Murina rozendaali*** Hill and Francis, 1984

*Murina rozendaali* Hill and Francis, 1984: 319; Gomantong, Sabah, Borneo, MALAYSIA 5°31’N, 118°4’E (Charles M. Francis, collector; BM(NH) 1983.360) [173].

**Common English name:** Rozendaal’s Tube-nosed Bat

**Barcode Index Number:** DNA barcodes of *M*. *rozendaali* are associated with BIN, BOLD:AAK8797 but there are no DNA barcodes from Peninsular Malaysia.

**Remarks:** Francis [196] noted that specimens from Peninsular Malaysia are smaller than specimens from Sabah in terms of weight and forearm lengtht, which may be due to ecological factors.

**IUCN status:** Vulnerable

**Recorded at: Pahang**: Krau Wildlife Reserve [11, 31; 196]; **Negeri Sembilan**: Pasoh [32]; **Selangor**: Semangkok Forest Reserve and Ulu Gombak [101]; **Terengganu**: Gunung Tebu Forest Reserve [101]; **Perak**: Kledang Saiong Forest Reserve [101], Temengor Forest Reserve [111].

All specimens of *M*. *rozendaali* from Peninsular Malaysia were collected in primary forests [11] although the species has been recorded in disturbed lowland forest in other regions [14].

***Murina suilla*** [Temminck, 1840]

*Vespertilio suillus* Temminck, 1840: 224, pl. 56; Tapos, Java, INDONESIA (Collector unknown; Type unknown) [82].

*Murina suilla* [163].

**Common English name:** Lesser Tube-nosed Bat

**Barcode Index Number:** DNA barcodes recorded as *M*. *suilla* are associated with four BINs, BOLD:AAE0000, BOLD:AAE0001, BOLD:AAE0003 and BOLD:ABX8091 but there are no DNA barcodes from Peninsular Malaysia.

**Remarks:** Simmons [98] recognises two subspecies: *M*. *s*. *suilla* (type locality: Java) and *M*. *s*. *canescens* (type locality: west Sumatra). Whether the two clusters suggested by our NJ analysis (S8 Fig) represent the two subspecies remains to be determined.

**IUCN status:** Least Concern

**Recorded at: Pahang**: Krau Wildlife Reserve [11, 42], Bentong [23], Kuala Atok, National Park [44], Tasik Bera Forest Reserve [56], Cameron Highland [60], Bukit Ibam, Kemasul, Jengka and Klau Besar [100]; **Perak**: Temengor Forest Reserve [46–48], Royal Belum State Park [66], Kledang Saiong Forest Reserve [101]; **Perlis:** Wang Kelian State Park [50]; **Selangor**: Bukit Kutu Wildlife Reserve [51], Ulu Gombak [53, 54]; **Kedah**: Ulu Muda Forest Reserve [57], Bukit Hijau [100]; **Kelantan**: Air Panas-Gua Musang [61], Gunung Stong State Park [100]; **Melaka**: Unspecified [68]; **Johor**: Gunung Panti [100]; **Negeri Sembilan**: Gunung Angsi Forest Reserve [100].

*M*. *suilla* has been recorded in lowland and hill forests [11, 14, 23].

### Family: Vespertilionidae (subfamily: Vespertilioninae)

#### *Arielulus circumdatus* and *A*. *societatis*

Heller and Volleth [53] reported that *Pipistrellus circumdatus* has a different structure of baculum and unique karyotypic characters (diploid number of chromosomes = 50, fundamental number = 48), in comparison with other *Pipistrellus* species which have a diploid number of chromosomes ranging from 26 to 44 and fundamental number = 50. They considered *P*. *circumdatus* to be conspecific with *P*. *societatis*, and transferred the taxon to the genus *Eptesicus* [53]. However, Hill and Francis [173] retained both *circumdatus* and *societatis* under *Pipistrellus* as two distinct species on the basis of the shorter palate, bony post-palate and toothrows which characterise *societatis*. Hill and Harrison [197] later examined the os penis of all the genera in Vepertilioninae and consequently established the subgenus *Arielulus* under *Pipistrellus*. Csorba and Lee [198] concluded that *Arielulus* is distinct from *Pipistrellus* based on the former’s distinctive coloration, short and wide rostrum, high and globular braincase, tricuspid upper incisor (I^1^), greatly reduced inner upper incisor (I^2^), small (often missing) first upper premolar (PM^2^), myotodont first and second lower molars (M_1_ and M_2_), very small Y-shaped baculum and the diploid number of chromosomes = 50 and consequently raised *Arielulus* as a genus.

***Arielulus circumdatus*** [Temminck, 1840]

*Vespertilio circumdatus* Temminck, 1840: 214; Tapos, Java, INDONESIA (Collector unknown; Type unknown) [82].

*Arielulus circumdatus* [98].

**Common English name:** Black Gilded Pipistrelle

**Barcode Index Number:** DNA barcodes recorded as *A*. *circumdatus* are associated with the BIN, BOLD:AAD8838 but there are no DNA barcodes from Peninsular Malaysia.

**Remarks:** Sing et al. [5] listed the species as *Eptesicus circumdatus* based on the nomenclature used by Heller and Volleth [53] (see remarks on the genus *Arielulus*).

**IUCN status:** Least Concern

**Recorded at: Selangor**: Ulu Gombak [53], **Pahang**: Unspecified [198].

*A*. *circumdatus* has been recorded in hill forest at an elevation of 1300–2000 m [14].

***Arielulus societatis*** [Hill, 1972]

*Pipistrellus societatis* Hill, 1972: 34; Base Camp, Gunong Benom, Pahang, MALAYSIA, 3°51’N, 102°11’E, 800ft (Boo-Liat Lim and Hoi-Sen Yong, collector; BM(NH) 1967.1605) [140].

*Arielulus societatis* [98].

**Common English name:** Benom Gilded Pipistrelle

**Barcode Index Number:** There are no DNA barcodes recorded under this name on BOLD.

**Remarks:** The species closely resembles *Arielulus circumdatus* Temminck, 1840 but has smaller forearms, post-palatal extension, toothrows and rostrum [140]. Heller and Volleth [53] considered *A*. *societatis* and *A*. *circumdatus* to be conspecific with the former being the lowland subspecies of the latter but this was refuted by Hill and Francis [173] on the basis of morphological characteristics (see remarks on the genus *Arielulus*). Both are recognised as distinct species by Simmons [98].

**IUCN status:** Vulnerable

**Recorded at: Selangor**: Ulu Gombak [53]; **Pahang**: Fraser Hill Forest Reserve [56], Gunong Benom [140].

*A*. *societatis* has been recorded in primary lowland and hill forests, and secondary forests, and found roosting in a hole of a tree trunk beside a forest stream [11, 14].

***Glischropus tylopus*** [Dobson, 1875]

*Vesperugo tylopus* Dobson, 1875: 473; Sabah, north Borneo, MALAYSIA (Collector unknown; BM(NH) 70.2.10.2) [199].

*Glischropus tylopus* [191].

**Common English name:** Thick-thumbed Pipistrelle

**Barcode Index Number:** Two DNA barcodes (RONP009-14 and RONP024-14) which are from Perak, Peninsular Malaysia are not assigned with any BIN due to their short sequence length (<500bp). However, other DNA barcodes recorded as *G*. *tylopus* which were collected in Vietnam and Laos are associated with the BIN, BOLD:AAC0085.

**Remarks:** All DNA barcodes (BIN, BOLD:AAC0085) recorded as *G*. *tylopus* from Vietnam and Laos (= Indochina) represent *G*. *bucephalus* which was recently described on the basis of longer forearms and distinctive cranial features [200]. Another species from Sumatra, Indonesia, *G*. *aquilus* is distinct from *G*. *tylopus* collected in Peninsular Malaysia on the basis of its darker colour and 12.4% divergence in cytochrome *b* mtDNA [201]. It is likely that the form occurring in Peninsular Malaysia represents *G*. *tylopus* sensu stricto based on the comparison of specimens from Peninsular Malaysia and Sabah = type locality (see Fig 6 in [201]). We did not perform any NJ analysis for *G*. *tylopus* as the DNA barcodes from Peninsular Malaysia are too short for comparison with other barcodes.

**IUCN status:** Least Concern

**Recorded at: Pahang**: Krau Wildlife Reserve [11], Tasik Chini [43], Tasik Bera Forest Reserve [56]; **Selangor**: Bukit Lanjan [40], Bukit Kutu Wildlife Reserve [51], Ulu Gombak [53, 54], Semangkok Forest Reserve [101]; **Kedah**: Ulu Muda Forest Reserve [57]; **Kelantan**: Air Panas-Gua Musang [61], Gua Musang [62]; **Perak**: Temengor Forest Reserve [111].

*G*. *tylopus* is a lowland forest inhabitant but has been recorded in hill forest [11, 14, 23]. Individuals have been found roosting in small groups in internodes of dead and broken bamboo, and sometimes in rock crevices and banana leaves, occasionally with *Tylonycteris* species [11, 14].

***Nyctalus noctula*** [Schreber, 1774] (?)

*Vespertilios noctula* Schreber, 1774: 166, pl. 52; FRANCE (Collector unknown; Type unknown) [202].

*Nyctalus noctula* [8].

**Common English name:** Eurasian Noctule

**Barcode Index Number:** DNA barcodes recorded as *N*. *noctula* are associated with BIN, BOLD:AAC7411, but there are no DNA barcodes from Peninsular Malaysia.

**Remarks:** Chasen [8] suggested that the species in Peninsular Malaysia may represents *N*. *n*. *labiate = labiatus* Hodgson, 1835 (type locality = Nepal) which also occurs in Pakistan and India [9]. *N*. *n*. *labiatus* is considered to be morphologically distinct from *N*. *noctula* and therefore, should be raised as a species [203]. The records of *N*. *noctula* from Peninsular Malaysia, if valid, may be referable to *“labiatus”* or *“plancyi”* [203].

**IUCN status:** Least Concern

**Recorded at:** There is only one record of *N*. *noctula* in this region which is an old skin dated 1838 purchased in Singapore [204] but its origin remains doubtful [23]. Based on the purchased skin, Dobson [204] included Peninsular Malaysia in the distribution range of *N*. *noctula*, which was followed by Corbet and Hill [9] and Medway [23]. We could not find recent records for the species although it is thought to occur in Peninsular Malaysia [1, 10]. We found two old records which reported two specimens of *Nyctalus* sp. in a National Park, **Pahang** [87] and Ulu Langat Forest Reserve, **Selangor** [88]. However, there are no specimens deposited in the DWNP collection.

*N*. *noctula* roosts in tree hollows and forages high above canopy [14, 23].

***Philetor brachypterus*** [Temmick, 1840]

*Vespertilio brachypterus* Temmick, 1840: 215, pl. 53; Padang district, Sumatra, INDONESIA (Collector unknown; Type unknown) [82].

*Philetor brachypterus* [23].

**Common English name:** Narrow-winged Brown Bat

**Barcode Index Number:** BOLD:AAF6860 (1 DNA barcode from Peninsular Malaysia; Fig 6)

**Remarks:** DNA barcodes recorded as *P*. *brachypterus* are associated with two BINs, BOLD: AAF6860 and BOLD:AAF6859. Hill and Francis [173] reported that specimens from Borneo and Peninsular Malaysia are similar in size, while Corbet and Hill [9] commented that size variation occurs within the species. Our NJ analysis suggested that the DNA barcode from Peninsular Malaysia (BM434-04) may represents a cryptic species (Fig 6), however, we retain the name *P*. *brachypterus* in our checklist pending further research.

**IUCN status:** Least Concern

**Recorded at: Johor**: Endau-Rompin National Park (BM434-04 was collected in year 2001 [4]); **Perak**: Unspecified [23]; **Selangor**: Unspecified [23], Ulu Gombak [53, 54].

*P*. *brachypterus* roosts in tree hollows and has been recorded in primary and secondary forests [14].

***Pipistrellus javanicus*** [Gray, 1838]

*Scotophilus javanicus* Gray, 1838: 498; Java, INDONESIA (Collector unknown; Type unknown) [205].

*Pipistrellus javanicus* [8].

**Common English name:** Javan Pipistrelle

**Barcode Index Number:** DNA barcodes recorded as *P*. *javanicus* are associated with two BINs, BOLD:AAC3383 and BOLD:AAL5777, but there are no DNA barcodes from Peninsular Malaysia.

**IUCN status:** Least Concern

**Recorded at: Perak**: Unspecified [23]; **Pulau Pinang**: Unspecified [23]; **Pahang**: Krau Wildlife Reserve [41], Gunung Benom, [140]; **Selangor**: Air Hitam Forest Reserve [55]; **Kedah**: Ulu Muda Forest Reserve [57].

*P*. *javanicus* has been recorded in wide variety of habitats including mangroves, lowland and hill forests, towns and rubber plantations, and found roosting in treeferns, fallen logs and caves [11, 14, 23].

***Pipistrellus stenopterus*** [Dobson, 1875]

*Vesperugo stenopterus* Dobson, 1875: 470; Sarawak, Borneo, MALAYSIA (Collector unknown; Type unknown) [199].

*Pipistrellus stenopterus* [52].

**Common English name:** Narrow-winged Pipistrelle

**Barcode Index Number:** There are no DNA barcodes recorded under this name on BOLD.

**IUCN status:** Least Concern

**Recorded at: Pahang**: Krau Wildlife Reserve [11, 32]; **Selangor**: Ulu Gombak [52]; **Kedah**: Ulu Muda Forest Reserve [57].

*P*. *stenopterus* has been recorded foraging in open areas and over rivers in forest and rubber plantations, and found roosting in tree hollows and under house roofs with *Scotophilus kuhlii* [11, 14].

***Pipistrellus tenuis*** [Temminck, 1840]

*Vespertilio tenuis* Temminck, 1840: 229; Sumatra, INDONESIA (Collector unknown; Type unknown) [82].

*Pipistrellus tenuis* [8].

**Common English name:** Least Pipistrelle

**Barcode Index Number:** DNA barcodes recorded as *P*. *tenuis* are associated with the BIN, BOLD:AAB2554, but there are no DNA barcodes from Peninsular Malaysia.

**IUCN status:** Least Concern

**Recorded at: Pahang**: Unspecified [23]; **Pulau Pinang**: Unspecied [23]; **Selangor**: Bukit Kutu Wildlife Reserve [51]; **Kedah**: Ulu Muda Forest Reserve [57]; **Melaka**: Sungai Udang Forest Reserve [68].

*P*. *tenuis* has been reported roosting in buildings in highly disturbed areas and in hollowed branches and among dead leaves in forests [14].

***Hesperoptenus blanfordi*** [Dobson, 1877]

*Vesperugo blanfordi* Dobson, 1877: 312; Tenasserim, east of Moulmein, south Burma = MYANMAR (Limborg, collector; Type unknown) [206].

*Hesperoptenus blanfordi* [8].

**Common English name:** Least False-serotine

**Barcode Index Number:** DNA barcodes recorded as *H*. *blanfordi* are associated with the BIN, BOLD:AAD5793, but there are no DNA barcodes from Peninsular Malaysia.

**IUCN status:** Least Concern

**Recorded at: Pahang**: Krau Wildlife Reserve [11, 41], Jengka in Temerloh [140]; **Selangor**: Ulu Gombak [53, 54].

*H*. *blanfordi* has been reported roosting in the entrances of limestone caves in small colonies and recorded foraging in open areas, in gaps created by fallen trees, and above rivers [11, 14].

***Hesperoptenus doriae*** [Peters, 1868]

*Vesperus (H*.*) doriae* Peters, 1868: 626; Sarawak, Borneo, MALAYSIA (Collector unknown; Type unknown) [207].

*Hesperoptenus doriae* [207].

**Common English name:** Doria's False-serotine

**Barcode Index Number:** There are no DNA barcodes recorded under this name on BOLD.

**IUCN status:** Data Deficient

**Recorded at: Selangor**: Air Hitam Forest Reserve [40], Ulu Gombak [54].

*H*. *doriae* has been reported roosting in a small colony of eight to ten individuals at overhanging rocks, near a stream [40], and in leaves of palm trees [14].

***Hesperoptenus tomesi*** Thomas, 1905

*Hesperoptenus tomesi* Thomas, 1905: 575; Malacca = Melaka, MALAYSIA (Collector unknown; Originally No. 190A in the collection of Mr. R. F. Tomes but currently as BM(NH) 1907.1.1.428) [208].

**Common English name:** Tome's False-serotine

**Barcode Index Number:** There are no DNA barcodes recorded under this name on BOLD.

**IUCN status:** Vulnerable

**Recorded at: Melaka**: Unspecified [208]; **Selangor**: Ulu Gombak [53, 54].

*H*. *tomesi* has been recorded in mature lowland forest [14].

***Hypsugo macrotis*** [Temminck, 1840]

*Vespertilio macrotis* Temminck, 1840: 218, pl. 54; Padang, Sumatra, INDONESIA (Collector unknown; Type unknown) [82].

*Pipistrellus imbricatus* [23]

*Pipistrellus macrotis* [209]

*Hypsugo macrotis* [98].

**Common English name:** Big-eared Pipistrelle

**Barcode Index Number:** There are no DNA barcodes recorded under this name on BOLD.

**Remarks:** *H*. *macrotis* was previously regarded as *Pipistrellus macrotis* [98]. The species was first reported from Peninsular Malaysia as *Pipistrellus imbricatus macrotis* [8] with only one locality record in the lowlands of Selangor [23]. Francis and Hill [209] later commented that the specimens recorded as *P*. *imbricatus macrotis* from Peninsular Malaysia [23] represent *P*. *macrotis* = *H*. *macrotis*.

**IUCN status:** Data Deficient

**Recorded at: Selangor**: lowlands of Selangor [23], Kuala Selangor [173]; **Negeri Sembilan:** Seremban [31]; **Kedah**: Ulu Muda Forest Reserve [57].

*H*. *macrotis* has been recorded in lowland forests and coastal lagoons near mangroves [14, 23] and recently in a school located in an urbanized habitat with small secondary forest fragments [31]. It is likely that the species may have adapted to human modified habitats [31], and the lack of its recent records may be due to surveys primarily target forest habitats.

***Scotophilus kuhlii*** Leach, 1821

*Scotophilus kuhlii* Leach, 1821: 72; INDIA (Collector unknown; Type unknown) [210].

*Scotophilus teminckii* [23].

*Scotophilus kuhlii teminckii* [9].

**Common English name:** Lesser Asian House Bat

**Barcode Index Number:** DNA barcodes recorded as *S*. *kulii* are associated with the BIN, BOLD:AAC0094, but there are no DNA barcodes from Peninsular Malaysia.

**Remarks:** Medway [23] reported *S*. *teminckii* from Peninsular Malaysia but Corbet and Hill [9] considered *S*. *teminckii* to be a synonym of *S*. *kuhlii*.

**IUCN status:** Least Concern

**Recorded at: Pahang**: Krau Wildlife Reserve [11]; **Selangor**: Bukit Kemandul [40], Bangi Forest Reserve [41], Ulu Gombak [54], Air Hitam Forest Reserve [55]; **Perak**: Selama [49]; **Kelantan:** Gunung Reng [62]; **Melaka**: Sungai Udang Forest Reserve [68].

*S*. *kuhlii* is associated with humans and often sighted hunting insects at lamp posts in urban areas [11]. The species roosts in large colonies, often under the roofs of buildings, under the fronds of palms, in hollowed dead trees in forests, and in hollowed old rubber trees in rubber plantations [11, 14, 23].

***Tylonycteris pachypus*** [Temminck, 1840]

*Vespertilio pachypus* Temminck, 1840: 217; Bantam, west Java, INDONESIA (Collector unknown; Type unknown) [82].

*Tylonycteris pachypus* [8].

**Common English name:** Lesser Bamboo Bat

**Barcode Index Number:** DNA barcodes recorded as *T*. *pachypus* are associated with two BINs, BOLD:AAC1209 and BOLD:AAC1210, but there are no DNA barcodes from Peninsular Malaysia.

**Remarks:** The BIN, BOLD:AAC1210 contains DNA barcodes recorded as *T*. *pachypus* and a single DNA barcode recorded as *T*. *robustula* (ABBSI217-10) (S9 Fig). We suspect the DNA barcode, ABBSI217-10 is a case of mis-identification as *T*. *pachypus* and *T*. *robustula* are differ in body size and coloration. Simmons [98] recognised five subspecies: *T*. *p*. *pachypus* (type locality: Java, Indonesia), *T*. *p*. *aurex* (type locality: India), *T*. *p*. *fulvidus* (type locality: Burma = Myanmar), *T*. *p*. *meyeri* (type locality: Philippines), and *T*. *p*. *bhakti* (type locality: Lombok Island, Indonesia).

**IUCN status:** Least Concern

**Recorded at: Pahang**: Krau Wildlife Reserve [11, 41], Tasik Bera Forest Reserve [56]; **Perak**: Temengor Forest Reserve [46, 47], Royal Belum State Park [66]; **Selangor**: Bukit Kutu Wildlife Reserve [51], Ulu Gombak [52, 53, 54], Air Hitam Forest Reserve [55]; **Kelantan**: Air Panas-Gua Musang [61], Gunung Reng and Gua Musang [62], Gunung Stong State Park [67]; **Johor**: Labis Forest Reserve [100].

*T*. *pachypus* roosts in small colonies in live standing bamboo stems, and enters the internodes through slits created by stem-boring beetle larvae [11, 14, 23].

***Tylonycteris robustula*** Thomas, 1915

*Tylonycteris robustula* Thomas, 1915: 227; Upper Sarawak, Borneo, MALAYSIA (Cecil J. Brooks, collector; BM(NH) 1911.1.18.8) [211].

*Tylonycteris malayana* Chasen, 1940: 52; Jor, Batang Padang Dist., Perak, MALAYSIA (Frederick N. Chasen, collector; BM(NH) 47.1433) [8].

*Tylonycteris robustula malayana* [98].

**Common English name:** Greater Bamboo Bat

**Barcode Index Number:** DNA barcodes recorded as *T*. *robustula* are associated with three BINs, BOLD:AAB3205, BOLD:AAB3206 and BOLD:AAC1210, but there are no DNA barcodes from Peninsular Malaysia (S9 Fig).

**IUCN status:** Least Concern

**Remarks:** The BIN, BOLD:AAC1210 contains a single DNA barcode of *T*. *robustula* (ABBSI217-10) and seven DNA barcodes of *T*. *pachypus*. We suspect the DNA barcode, ABBSI217-10 is a case of mis-identification as *T*. *pachypus* and *T*. *robustula* are differ in body size and coloration. Simmons [98] recognised two subspecies under *T*. *robustula*: *T*. *r*. *robustula* (type locality: Borneo) and *T*. *r*. *malayana* (type locality: Peninsular Malaysia).

**Recorded at: Pahang**: Krau Wildlife Reserve [11, 41], Tasik Chini [43], Tasik Bera Forest Reserve and Fraser Hill Forest Reserve [56]; **Selangor**: Ulu Gombak [23, 52, 53, 54], Bukit Lanjan [40], Bukit Kutu Wildlife Reserve [51], Air Hitam Forest Reserve [55], Semangkok Forest Reserve [101]; **Perak**: Temengor Forest Reserve [46, 47], Royal Belum State Park [66]; **Kedah**: Ulu Muda Forest Reserve [57]; **Kelantan**: Gunung Reng [62], Gunung Stong State Park, [67].

*T*. *robustula* roosts in internodes of large, often dead bamboo stems by entering the internodes through slits made by chrysomelid beetles and has been reported roosting in small harem groups, with one adult male and up to six females in one group [11, 14, 23]. Solitary males have also been reported [23].

### Family: Vespertilionidae (subfamily: Myotinae)

***Myotis adversus*** [Horsfield, 1824] (?)

*Vespertilio adversus* Horsfield, 1824: part 8; Java, INDONESIA (Collector unknown; Type unknown) [102].

*Myotis adversus* [8].

**Barcode Index Number:** There are no DNA barcodes recorded under this name on BOLD.

**IUCN status:** Least Concern

**Recorded at: Perak:** Unspecified [23].

***Myotis ater*** [Peters, 1866]

*Vespertilio ater* Peters, 1866: 18; Ternate Island, Moluccas, INDONESIA (Collector unknown; Type unknown) [212]

*Myotis ater* [9].

**Common English name:** Peters's Myotis

**Barcode Index Number:** A DNA barcode recorded as *M*. cf. *ater* (BM487-04) is from Peninsular Malaysia but is not associated with any BIN due to its short sequence length (<500 bp). Other DNA barcodes recorded as *M*. *ater* are associated with the BIN, BOLD:AAA8748.

**Remarks:** The only BIN (BOLD:AAA8748) that is associated with *M*. *ater* also contains DNA barcodes recorded as *M*. *muricola* (Fig 6). These taxa were previously considered to be conspecific [213] but Francis and Hill [214] recognised two species occuring sympatrically in Malaysia, and commented that specimens from Peninsular Malaysia recorded as *M*. *ater* are larger than *M*. *ater* from elsewhere. As *M*. *muricola* is putatively a species complex (refer to remarks on *M*. *muricola*), further studies of *M*. *ater* and *M*. *muricola* are required to resolve the relationship between these taxa.

**IUCN status:** Least Concern

**Recorded at: Pahang**: Krau Wildlife Reserve [11], Cameron Highland [60]; **Perak**: Bukit Jerneh Cave and Tumang Lembing Cave [30]; **Kedah**: Ulu Muda Forest Reserve [57].

*M*. *ater* has been reported roosting in caves, either solitarily or in small colonies and recorded foraging in open areas such as gaps created by fallen trees, midstorey openings and forest edge [11, 14].

***Myotis muricola*** [Gray, 1846]

*Vespertilio muricola* Gray, 1846: 4; NEPAL (Brian Houghton Hodgson, collector; Type unknown) [215].

*Myotis muricola* [8].

**Common English name:** Asian Whiskered Myotis

**Barcode Index Number:** DNA barcodes recorded as *M*. *muricola* are associated with two BINs, BOLD:AAA8747 and BOLD:AAA8748, but there are no DNA barcodes from Peninsular Malaysia. A DNA barcode of *M*. cf. *muricola* (RONP037-14) from Peninsular Malaysia but is not associated with any BIN due to its short sequence length (<500 bp).

**Remarks:** Francis and Hill [214] commented that *M*. *muricola* from localities across Southeast Asia exhibit moderate morphological variation. Wiantoro et al. [216] suggested *M*. *muricola* was a species complex with 31.5% divergence in cytochrome *b* mtDNA between (i) *M*. *muricola* Western (including DNA sequences from Krakatau, Bali, Lombok, Sumba, Sumbawa, Flores Lembata and Pantar) and (ii) *M*. *muricola* Eastern (including DNA sequences from Sumatra, Peninsular Malaysia, Phillipines and Asian mainland). *M*. *muricola* Eastern exhibited 9.5% divergence in cytochrome *b* mtDNA within the group whereas *M*. *muricola* Western exhibited 8% divergence within the group. *M*. *muricola* Western is further segregated into two subgroups with 7.2% divergence in cytochrome *b* mtDNA: (i) Sumatra-Asian subgroup (consists of DNA sequences from Sumatra, Peninsular Malaysia and Asian mainland) and (ii) Bornean subgroup (consists of DNA sequences from Sarawak, Sabah and Kalimantan).

*M*. *muricola* was previously considered to be a subspecies of *M*. *mystacinus* [23, 213], but Hill [217] reviewed the status of *M*. *mystacinus* and stated that the form occurring in Peninsular Malaysia represents *M*. *muricola muricola*. Similarly, Corbet and Hill [9] concluded that *M*. *mystacinus* was not present in Malaysia and the taxon occurring in Peninsular Malaysia represents *M*. *muricola*. Wiantoro et al. [216] reported that *M*. *mystacinus* and *M*. *muricola* Western are 17.1% divergent in cytochrome *b* mtDNA, while *M*. *mystacinus* and *M*. *muricola* Eastern showed 26% divergence. After the division of *M*. *muricola* and *M*. *mystacinus*, *M*. *mystacinus* was thought to only occur in Europe until Bates et al. [218] recorded the species in Myanmar. Further surveys are required to determine whether the reports of *M*. *mystacinus* in Peninsular Malaysia represents *M*. *muricola* or whether both species exist sympatrically in Peninsular Malaysia.

The BIN, BOLD:AAA8748 contains DNA barcodes recorded as *M*. *muricola* and *M*. *ater* (see remarks on *M*. *ater* and Fig 6). The species were previously considered to be conspecific [213] but were later recognised as distinct based on body size variation [217] which occurred sympatrically in Malaysia [214]. We did not include the DNA barcode recorded as *M*. cf. *muricola* (RONP037-14) from Peninsular Malaysia in our NJ analysis due to its short sequence length, but noted that the DNA barcode did not cluster with DNA barcodes of *M*. *muricola* on a taxon ID tree generated in BOLD (hence “cf. *muricola*”).

**IUCN status:** Least Concern

**Recorded at: Selangor**: Ulu Gombak [5, 53, 54], Bukit Kutu Wildlife Reserve [51], Air Hitam Forest Reserve [55]; **Pahang**: Krau Wildlife Reserve [11]; **Negeri Sembilan**: Pasoh Forest Reserve [45]; **Perak**: Temengor Forest Reserve [46, 47]; **Johor:** Endau-Kota Tinggi Forest Reserve [56]; **Kelantan**: Air Panas-Gua Musang [61], Gunung Stong State Park [67].

Recorded as *M*. *mystacinus* at: **Pulau Pinang**: Unspecified [23]; **Selangor**: Air Hitam Forest Reserve [40], Ulu Gombak [52], Batu Caves [219]; **Pahang**: Krau Wildlife Reserve [41], Tasik Chini [43].

*M*. *muricola* has been reported roosting in small colonies of up to ten individuals at vegetated cave entrances and in tightly rolled central leaves of banana plants, in both forested and agricultural areas [11, 14, 23].

***Myotis hasseltii*** [Temminck, 1840]

*Vespertilio hasseltii* Temminck, 1840: 225; Bantam, Java, INDONESIA (Collector unknown; Type unknown) [82].

*Myotis hasseltii* [8].

**Common English name:** Hasselt's Myotis

**Barcode Index Number:** DNA barcodes recorded as *M*. *hasseltii* are associated with the BIN, BOLD:AAC1504, but there are no DNA barcodes from Peninsular Malaysia.

**IUCN status:** Least Concern

**Recorded at: Kedah**: **Pahang**: Krau Wildlife Reserve [11]; Kuah in Pulau Langkawi [23, 140]; **Selangor**: Unspecified mangrove forest [23]; **Perlis**: Kangar [140]; **Perak**: Kuala Gula [140].

*M*. *hasseltii* has been reported roosting in limestone caves and rock crevices, and recorded foraging near coastal areas, mangroves and water bodies such as rivers, lakes and seashores [11, 14, 23]. The species is presumed to skim small fishes and insects from water surface [11].

***Myotis hermani*** Thomas, 1923

*Myotis hermani* Thomas, 1923: 252; Sabang, northwest Sumatra, INDONESIA (G. Herman, collector; BM(NH) 1923.1.2.13) [36].

**Common English name:** Herman's Myotis

**Barcode Index Number:** There are no DNA barcodes recorded under this name on BOLD.

**IUCN status:** Data Deficient

**Recorded at: Perak**: Temengor Forest Reserve [46, 47, 71].

*M*. *hermani* has been recorded in lowland forests [14].

***Myotis horsfieldii*** [Temminck, 1840]

*Vespertilio horsfieldii* Temminck, 1840: 226; Mount Gede, Java, INDONESIA (Collector unknown; Type unknown) [82].

*Myotis horsfieldii* [8].

**Common English name:** Horsfield’s Myotis

**Barcode Index Number:** BOLD:AAB9975 (1 DNA barcode from Peninsular Malaysia; Fig 6)

**Remarks:** DNA barcodes recorded as *M*. *horsfieldii* are associated with four BINs, BOLD:AAB9973, BOLD:AAB9974, BOLD:AAB9975, and BOLD:ACH4644 (Fig 6). Five subspecies have been described under *M*. *horsfieldii*: *M*. *h*. *horsfieldii* (type locality: Java), *M*. *h*. *dryas* (type locality: Andaman Islands), *M*. *h*. *peshwa* (type locality: India), *M*. *h*. *jeannei* (type locality: Philippines) and *M*. *h*. *deignani* (type locality: Thailand) [98]. The form occurring in Peninsular Malaysia represents the *M*. *h*. *horsfieldii* [9].

**IUCN status:** Least Concern

**Recorded at: Pahang**: Krau Wildlife Reserve [11], Merapoh [40], Cameron Highland [60]; **Pulau Pinang** [23]; **Kuala Lumpur**: Ampang [23, 140]; **Perak**: Temengor Forest Reserve [46, 47]; **Kelantan:** Gunung Reng [62]; **Terengganu**: Bukit Dendong [97].

*M*. *horsfieldii* has been recorded roosting in limestone caves, in crevices of rocks and boulders, and foraging near forest streams to presumably skim insects from water surface [11, 14, 23].

***Myotis federatus*** Thomas, 1916

*Myotis peytoni federatus* Thomas, 1916: 3; Semangko Paas, MALAYSIA, 2700 ft (Collector unknown; BM(NH) 1916.4.20.5) [178].

*Myotis montivagus federatus* [9].

*Myotis federatus* [35].

**Barcode Index Number:** There are no DNA barcodes recorded under this name on BOLD. DNA barcodes recorded as *M*. *montivagus* are associated with two BINs, BOLD:AAC5917 and BOLD:AAU0309, but there are no DNA barcodes from Peninsular Malaysia.

**Remarks:** *M*. *federatus* was previousy considered to be a subspecies of *M*. *montivagus* [9] based on dental characters [213]. Görföl et al. [35], however, noted that *M*. *federatus* has smaller forearms, a larger skull and smaller middle upper premolars (P3). The species have distinct geographical ranges: *M*. *federatus* is confined to Peninsular Malaysia whereas *M*. *montivagus* is distributed from south China to northern Myanmar [35]. Previous records of *M*. *montivagus* from Peninsular Malaysia [1, 5, 50, 111] should be updated to *M*. *federatus* [35].

**IUCN status:** Not Evaluated but Least Concern as *M*. *montivagus*.

**Recorded at:** Previously recorded as *M*. *montivagus* at: **Selangor**: Genting Semangkok [23], Batu Caves (HNHM 98.14.31 [35]), Ulu Gombak [54]; **Pahang**: Genting Highland [54]; **Perak**: Temengor Forest Reserve [46, 47]; **Perlis**: Wang Kelian State Park [50].

*M*. *federatus* has been recorded in primary and secondary forests with elevations up to 1,000 m [14].

***Myotis ridleyi*** [Thomas, 1898]

*Pipistrellus ridleyi* Thomas, 1898: 361; Selangor, MALAYSIA (H. N. Ridley, collector; BM(NH) 1898.3.13.5) [220].

*Myotis ridleyi* [140].

**Common English name:** Ridley's Myotis

**Barcode Index Number:** There are no DNA barcodes recorded under this name on BOLD.

**IUCN status:** Near Threatened

**Recorded at: Pahang**: Gunong Benom [140] in Krau Wildlife Reserve [11, 32]; **Perak**: Bukit Jerneh Cave and Tumang Lembing Cave [30], Temengor Forest Reserve [46, 47], Kledang Saiong Forest Reserve [101]; **Negeri Sembilan**: Pasoh Forest Reserve [45], Gunung Angsi Forest Reserve [100]; **Selangor**: Ulu Gombak [54]; **Johor**: Endau-Kluang Forest Reserve and Endau-Kota Tinggi Forest Reserve [56], Gunung Panti [100]; **Kedah**: Ulu Muda Forest Reserve [57].

*M*. *ridleyi* has been recorded only at understory of lowland forests, suggesting that the species is confined to forest interior. Individuals have been reported roosting in caves and under fallen logs and rocks [11, 14].

***Myotis siligorensis*** [Horsfield, 1855]

*Vespertilio siligorensis* Horsfield, 1855: 102; Siligori, NEPAL (Brian Houghton Hodgson, collector; Type unknown) [221].

*Myotis siligorensis* [23].

**Common English name:** Small-toothed Myotis

**Barcode Index Number:** DNA barcodes recorded as *M*. *siligorensis* are associated with five BINs, BOLD:AAA9718, BOLD:AAA9719, BOLD:AAA9720, BOLD:AAA9721, and BOLD:ACF1046, but there are no DNA barcodes from Peninsular Malaysia.

**Remarks:** Our NJ analysis suggested that *M*. *siligorensis* may be a species complex (S10 Fig). Simmons [98] recognised four subspecies: *M*. *s*. *siligorensis* (type locality: Nepal), *M*. *s*. *sowerbyi* (type locality: China), *M*. *s*. *alticraniatus* (type locality: Vietnam) and *M*. *s*. *thaianus* (type locality: Thailand). Whether the five BINs correspond to the described subspecies remains to be determined.

**IUCN status:** Least Concern

**Recorded at: Pahang**: Krau Wildlife Reserve [11, 41], Cheras Cave [23], Tasik Chini [43], Kuantan [140]; **Perlis**: Wang Kelian State Park [50]; **Kedah**: Ulu Muda Forest Reserve [57].

*M*. *siligorensis* has been recorded roosting in rock crevices and fissures in caves, often in small colonies at forest edges, in primary and secondary forests [14, 23]. Individuals have been recorded foraging near street lights at research station [11].

## Supporting information

S1 Fig NJ tree of DNA barcodes recorded as *Asellia stoliczkana* on BOLD. (PDF)

S2 Fig NJ tree of DNA barcodes recorded as *Coelops frithi* on BOLD. (PDF)

S3 Fig NJ tree of DNA barcodes recorded as *Hipposideros pomona* on BOLD. (PDF)

S4 Fig NJ tree of DNA barcodes recorded as *Rhinolophus acuminatus* on BOLD. (PDF)

S5 Fig NJ tree of DNA barcodes recorded as *Rhinolophus macrotis* on BOLD. (PDF)

S6 Fig NJ tree of DNA barcodes recorded as *Rhinolophus pusillus* on BOLD. (PDF)

S7 Fig NJ tree of DNA barcodes recorded as *Murina huttoni* on BOLD. (PDF)

S8 Fig NJ tree of DNA barcodes recorded as *Murina suilla* on BOLD. (PDF)

S9 Fig NJ tree of DNA barcodes recorded as *Tylonycteris pachypus* on BOLD. (PDF)

S10 Fig NJ tree of DNA barcodes recorded as *Myotis siligorensis* on BOLD. (PDF)

S1 File Checklist of bats of Peninsular Malaysia. (XLS)
