# Supplementary figures and images for: A checklist of the bats of Peninsular Malaysia and progress towards a DNA barcode reference library
Source: PLoS One. 2017 Jul 25;12(7):e0179555. doi: 10.1371/journal.pone.0179555 (PMC5526618; doi:10.1371/journal.pone.0179555)

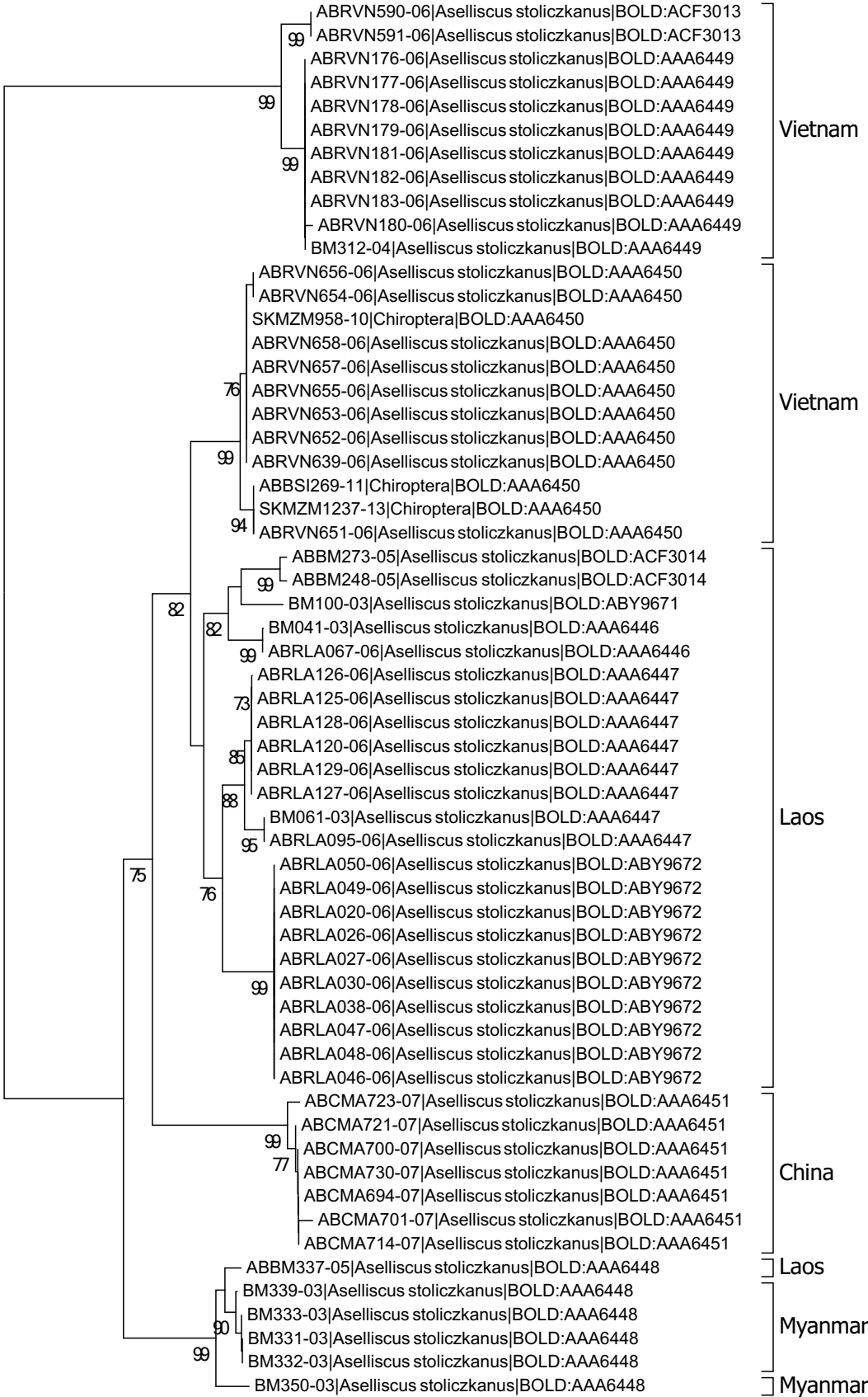

0.01

Supplement: S1 Fig — (PDF) [file pone.0179555.s001.pdf]

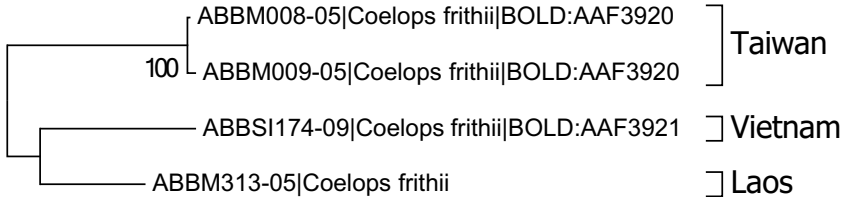

0.01

Supplement: S2 Fig — (PDF) [file pone.0179555.s002.pdf]

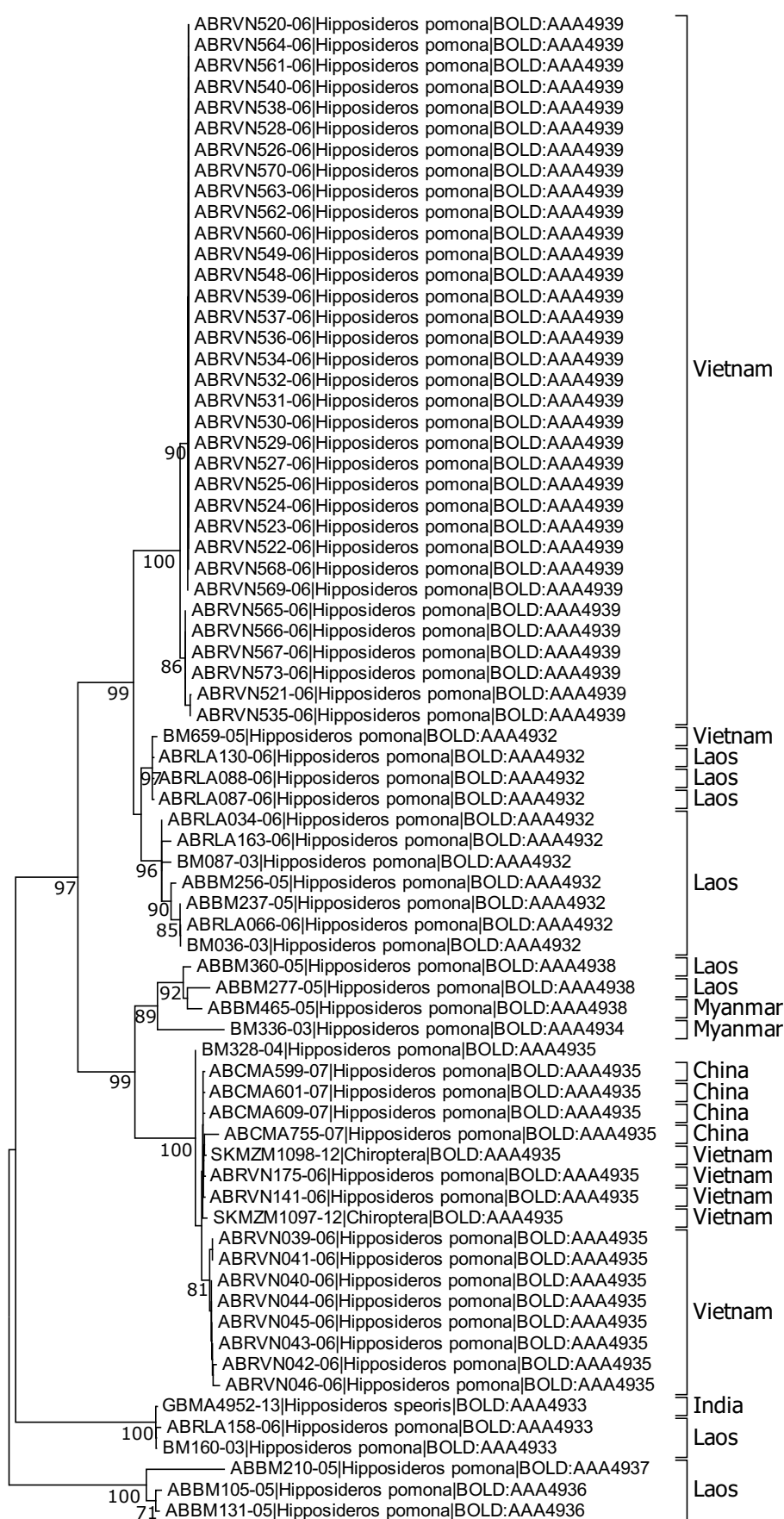

0.01

Supplement: S3 Fig — (PDF) [file pone.0179555.s003.pdf]

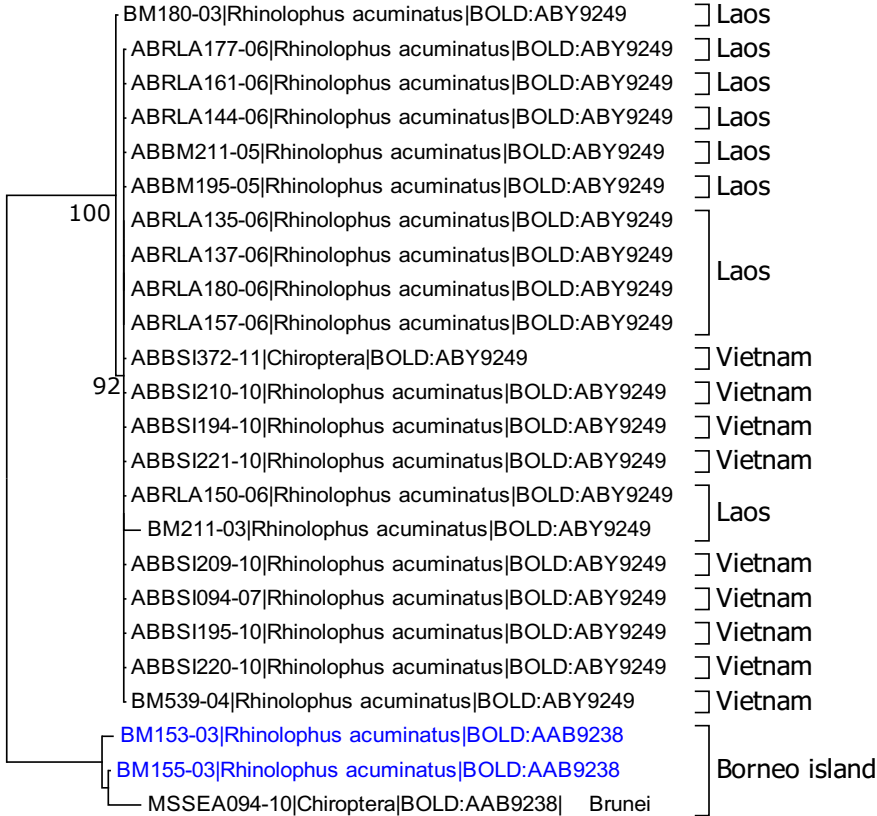

0.01

Supplement: S4 Fig — (PDF) [file pone.0179555.s004.pdf]

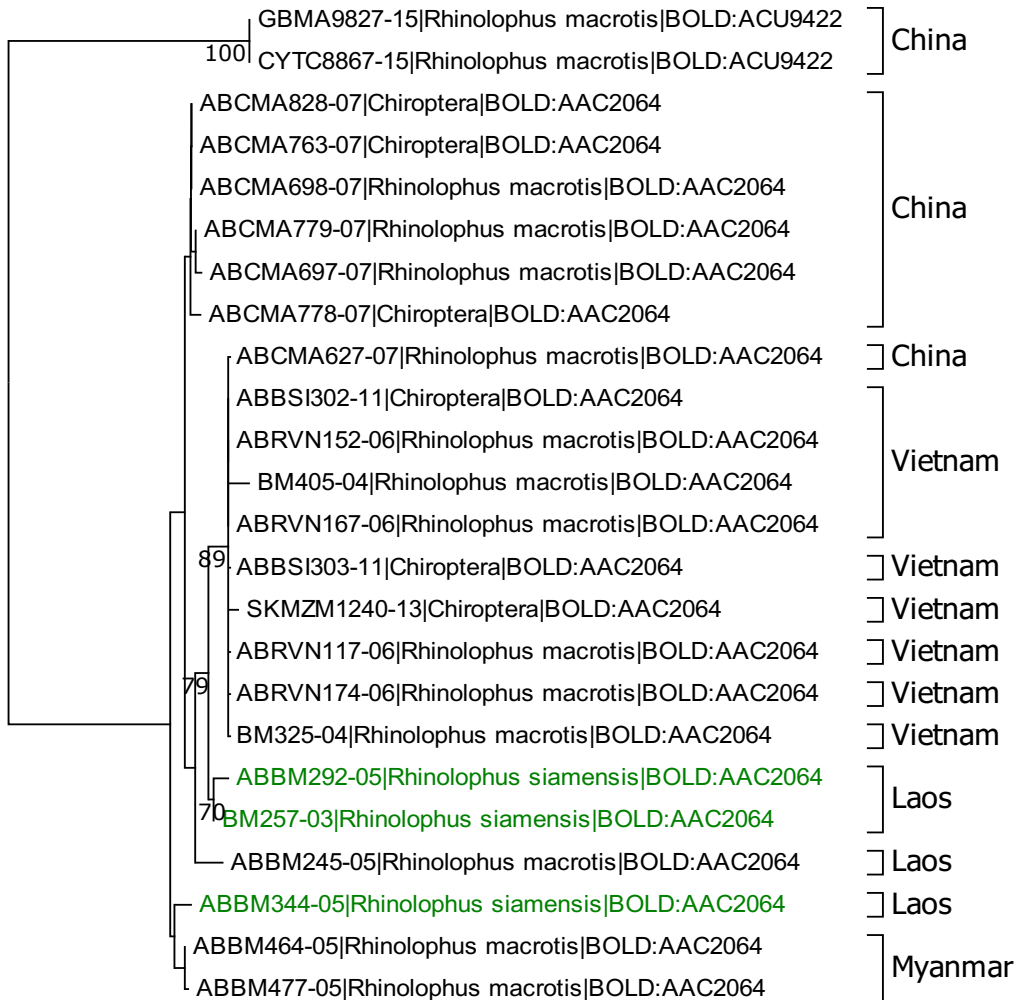

Supplement: S5 Fig — (PDF) [file pone.0179555.s005.pdf]

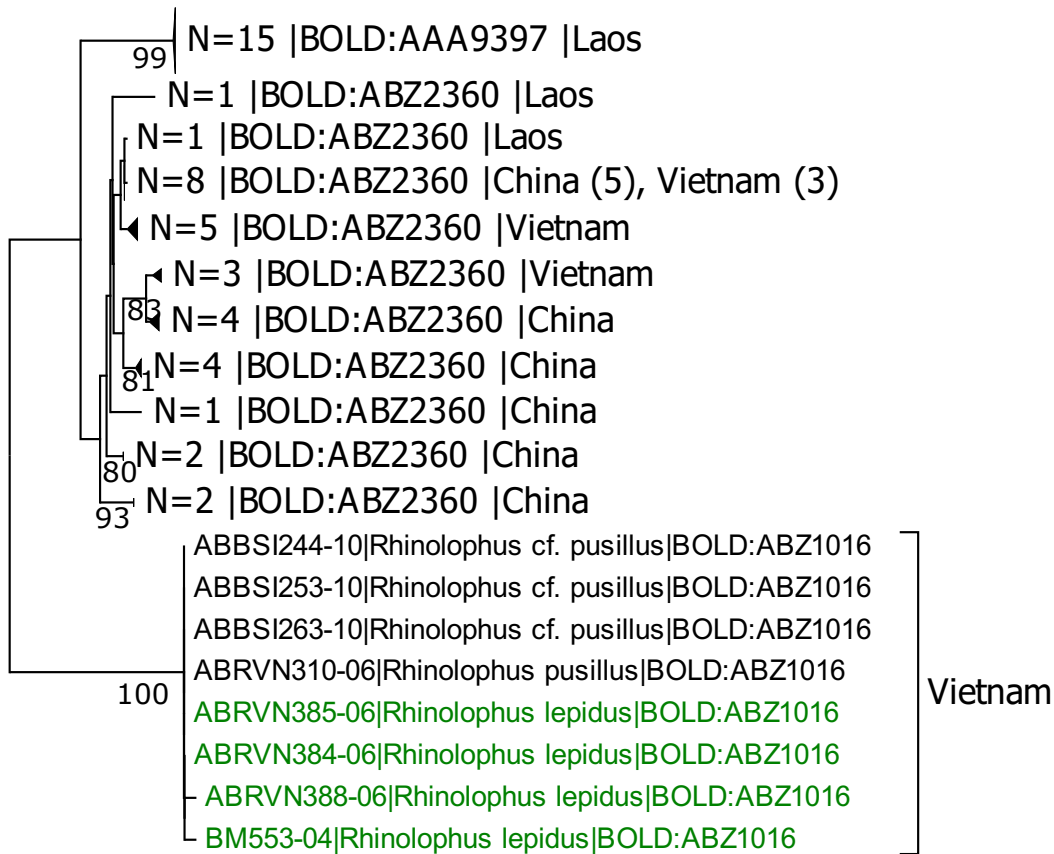

Supplement: S6 Fig — (PDF) [file pone.0179555.s006.pdf]

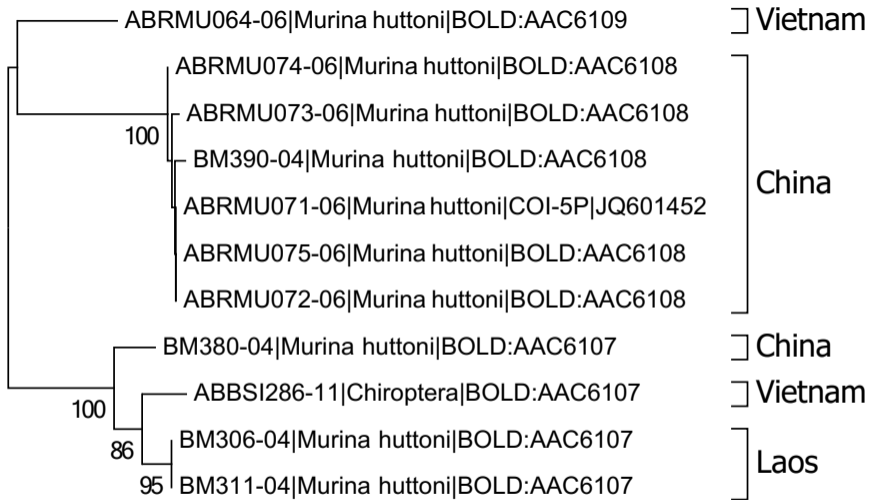

Supplement: S7 Fig — (PDF) [file pone.0179555.s007.pdf]

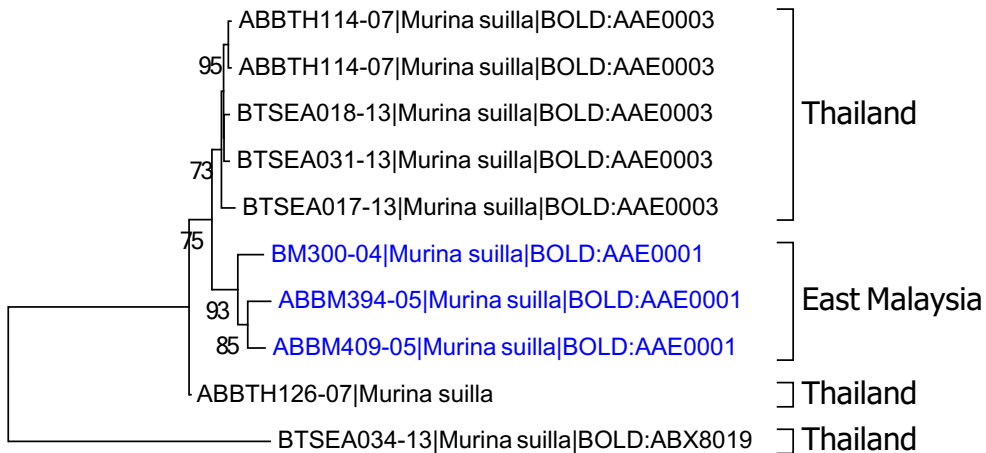

0.01

Supplement: S8 Fig — (PDF) [file pone.0179555.s008.pdf]

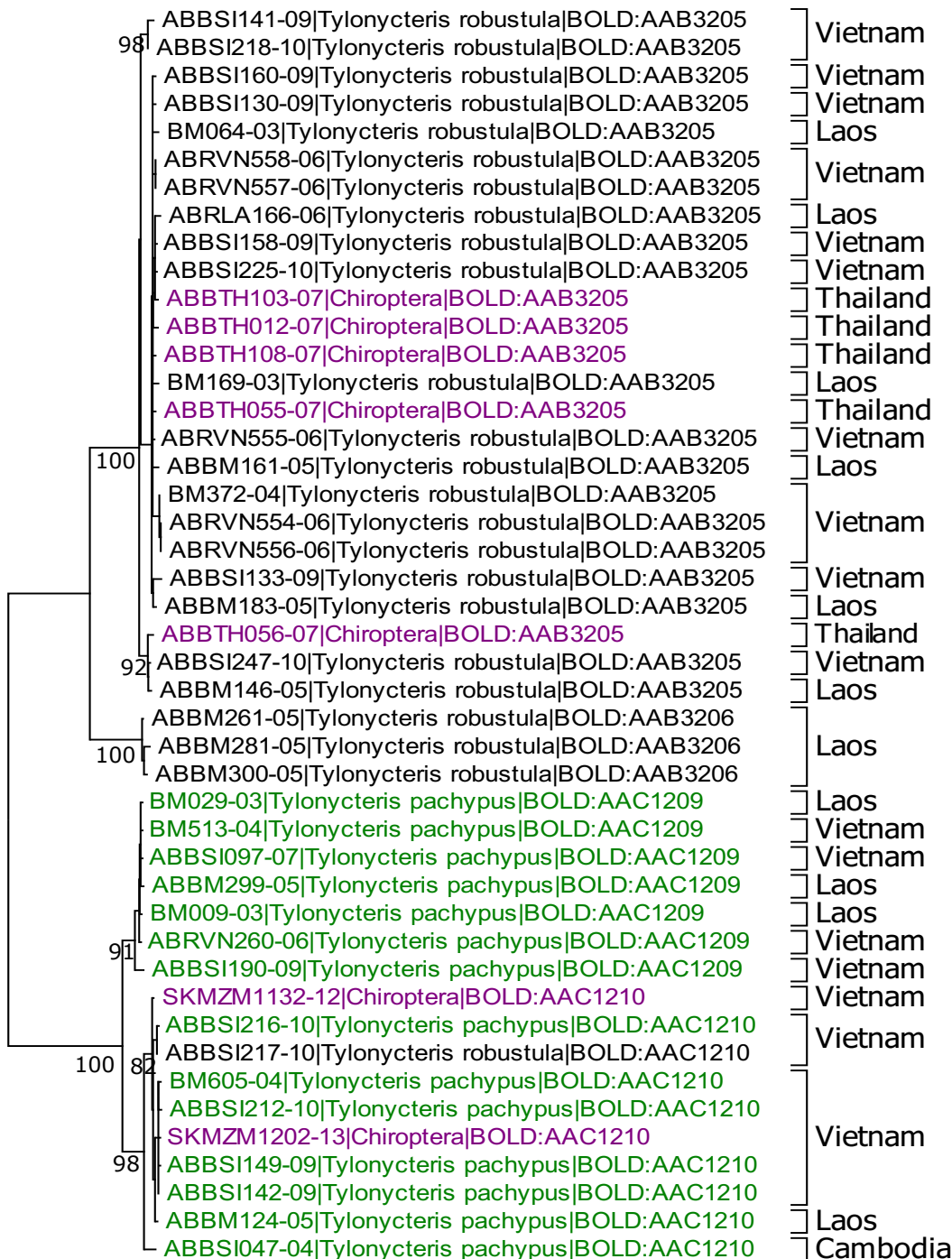

Supplement: S9 Fig — (PDF) [file pone.0179555.s009.pdf]

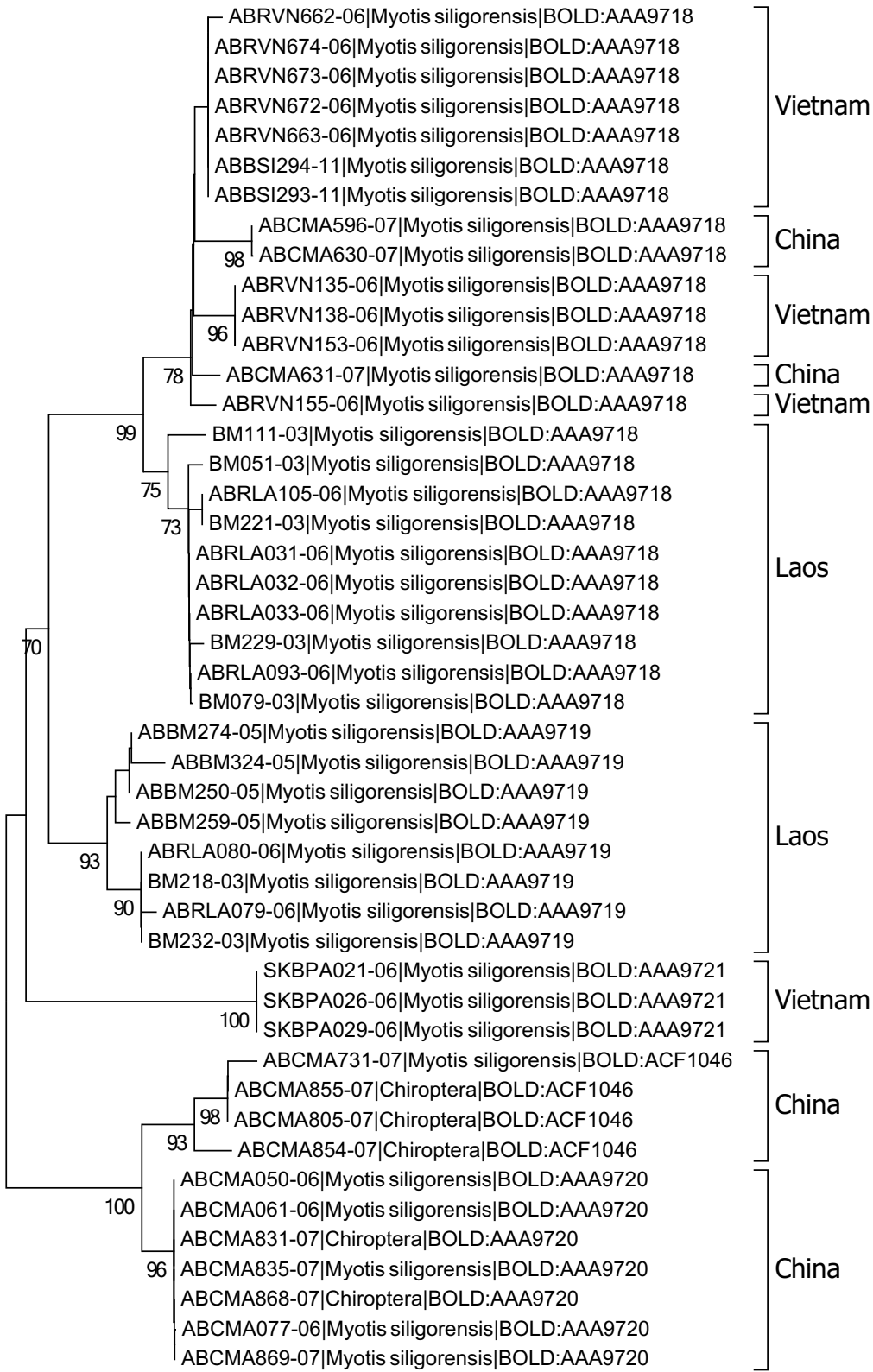

0.01

Supplement: S10 Fig — (PDF) [file pone.0179555.s010.pdf]
